# History of antibiotic adaptation influences microbial evolutionary dynamics during subsequent treatment

**DOI:** 10.1371/journal.pbio.2001586

**Published:** 2017-08-08

**Authors:** Phillip Yen, Jason A. Papin

**Affiliations:** Department of Biomedical Engineering, University of Virginia, Charlottesville, Virginia, United States of America; Massachusetts Institute of Technology, United States of America

## Abstract

Antibiotic regimens often include the sequential changing of drugs to limit the development and evolution of resistance of bacterial pathogens. It remains unclear how history of adaptation to one antibiotic can influence the resistance profiles when bacteria subsequently adapt to a different antibiotic. Here, we experimentally evolved *Pseudomonas aeruginosa* to six 2-drug sequences. We observed drug order–specific effects, whereby adaptation to the first drug can limit the rate of subsequent adaptation to the second drug, adaptation to the second drug can restore susceptibility to the first drug, or final resistance levels depend on the order of the 2-drug sequence. These findings demonstrate how resistance not only depends on the current drug regimen but also the history of past regimens. These order-specific effects may allow for rational forecasting of the evolutionary dynamics of bacteria given knowledge of past adaptations and provide support for the need to consider the history of past drug exposure when designing strategies to mitigate resistance and combat bacterial infections.

## Author summary

Bacteria readily adapt to their environments and can develop ways to survive and grow in the presence of antibiotics. While many studies have investigated how bacteria evolve to become resistant to single drugs, it is unclear how adaptation to other drugs and environments in the past affect the way bacteria adapt to new drugs and environments. In this study, we allowed bacteria in a laboratory setting to adapt to three different antibiotics. We first exposed wild-type susceptible bacteria to high concentrations of the three antibiotics individually and then exposed these populations to each of the other drugs. By tracking the levels of resistance to all three drugs in all of the treatments, we identified cases in which past adaptation to one treatment influenced subsequent evolutionary dynamics with regard to both phenotypes (levels of resistance) and genotypes (genes that became mutated). Additionally, by allowing bacterial isolates originating from human patients to adapt to the three drugs, we recapitulated a subset of the adaptation history-dependent evolutionary dynamics. Overall, this study sheds light on how adaptation history in bacteria can potentiate or constrain the rise of multidrug resistance, depending on the particular order of drugs used in a sequential therapy.

## Introduction

Antibiotic resistance is a growing healthcare concern whereby bacterial infections are increasingly difficult to eradicate due to their ability to survive antibiotic treatments [1]. There have been reported cases of resistance for nearly every antibiotic we have available [2]. Coupled with the fact that the antibiotic discovery pipeline has slowed over the past few decades [3], there is a dire need to find better treatment strategies using existing antibiotics that can slow or even reverse the development of resistance.

Adaptive laboratory evolution is a technique that can be used to study and test evolutionary principles in a highly controlled laboratory setting [4]. Microorganisms with short generation times such as bacteria are especially amenable to adaptive laboratory evolution and can be adapted to an environment through repeated cycles of growth in a specific media environment, dilution of the culture, and subsequent passaging into fresh media [5]. Multiple replicates for each condition can be evolved in parallel to investigate the reproducibility of evolutionary dynamics [6]. The evolutionary trajectories of the bacteria can be measured as they adapt to different nutrients and stressors over time [7]. Whole-genome resequencing on the evolved strains can subsequently be used to determine the mutations that occurred that may be associated with the observed phenotypes [8,9].

Adaptive laboratory evolution can be used to study the development of antibiotic resistance in bacterial pathogens [10]. Resistance to antibiotics is an evolutionary response of bacteria to withstand and survive the effects of the stressor. Deliberately evolving bacteria to withstand antibiotics through experimental evolution can yield insights into the evolutionary dynamics and trajectories of this adaptive process [11,12]. These evolution experiments can provide a longer-term perspective, which can yield information for the design of novel treatment strategies that can reduce the rate of resistance evolution or potentially even reverse the effects of resistance [13–15].

Recent studies have explored how adaptation to an antibiotic can cause bacteria to concurrently become more susceptible or more resistant to other drugs, an effect termed collateral sensitivity or collateral resistance [14,16,17]. Collateral sensitivities between drugs have been used to design drug cycling strategies and to explain the decreased rate of adaptation to certain antibiotics [12,14,18–23]. Drug deployment strategies that exploit such collateral sensitivities between pairs of antibiotics to minimize resistance evolution have been tested in vitro. A recent study determined the collateral sensitivity drug interaction network in *Escherichia coli* and demonstrated how an alternating sequential treatment of 2 reciprocal, collaterally sensitive antibiotics can slow down the rate of resistance evolution [14]. In this drug cycling strategy, the development of resistance to 1 drug concurrently increased the sensitivity to the second drug, and this allowed wild-type cells to outcompete the resistant cells when exposed to the second drug. In a different study, evolution experiments of alternating sequential therapies of pairs of antibiotics were performed on *Staphylococcus aureus*, and the study showed that the alternating treatments slowed the rate of resistance development compared to single-drug treatments [12]. Consistent with the *E*. *coli* study, this study found that collateral sensitivities could explain the evolutionary constraints in the cases in which alternating treatment resulted in decreased resistance development compared to the single-drug treatment.

Most of the prior studies that test the use of alternating antibiotic therapies to reduce the rate of resistance development employ an adaptive laboratory evolution scheme in which the drugs are switched at daily or subdaily intervals with the purpose of testing if rapidly changing antibiotic environments can diminish the rate of drug-resistance adaptation [12,19,23,24]. In this study, we expand on these prior works, but we are not focused on studying the evolutionary dynamics of bacteria adapted to rapidly changing drug environments. Rather, we explore the evolutionary dynamics of sustained, longer treatments of drugs and how the development of high levels of resistance to 1 drug influences the subsequent dynamics of sustained adaptation to a second drug. In clinical settings, when antibiotic cycling strategies are employed, they are used typically at the level of the hospital ward, and the cycling of antibiotics are often done at monthly intervals [25,26]. The rationale here is that if resistance to 1 drug arises after frequent use in a ward, switching to an antibiotic of a different class may allow resistance rates to the withdrawn drug to stabilize or even fall, enabling the first drug to be efficiently reintroduced again at a later time [27]. This practice of cycling drugs of different classes over the course of monthly intervals is done empirically, and it remains unclear how these regimens constrain the evolutionary dynamics of antibiotic resistance development. Here, we explore the evolutionary trajectories of bacteria as they evolve high levels of resistance to 1 antibiotic and the subsequent trajectories as the selection pressure from the first drug is withdrawn and replaced with the sustained pressure of a different drug. It remains unexplored how prior adaptation to 1 drug environment affects the evolutionary dynamics of a bacterial population during subsequent adaptation to a second drug in terms of the amount of resistance it can potentially develop and the resistance profile of the first drug. Collateral sensitivities and collateral resistances between 2 drugs have been studied in the context of adaptation to single drugs [21], i.e., as bacterial populations evolve and become resistant to 1 drug, do the cultures **concurrently** become more resistant or sensitive to other drugs? In this study, we focus not on if bacteria become concurrently more resistant or sensitive to other drugs, but rather, if adaptation to 1 drug constrains or potentiates the evolutionary dynamics to sustained adaptation to a second drug. How does the history of adaptation to 1 drug influence the subsequent adaptation to a second drug? If there are such historical dependencies, can we use this knowledge to design sequential therapies that slow down the evolution of resistance to the drugs used? What happens to the previously developed resistance once the drug pressure is taken away or switched to a different drug? Do compensatory adaptations sustain the high resistance, or do the bacteria revert and become susceptible again [28]? The answers to these questions are important for understanding how bacteria adapt to different antibiotic environments. Bacterial pathogens have complex evolutionary histories and elucidation of any historical dependencies of antibiotic resistance evolution would allow for rational forecasting of future resistance development and would aid in the design of strategies for mitigating antibiotic resistance.

## Results

### Adaptive evolution of *P*. *aeruginosa* to sequences of antibiotics

**Fig 1:**
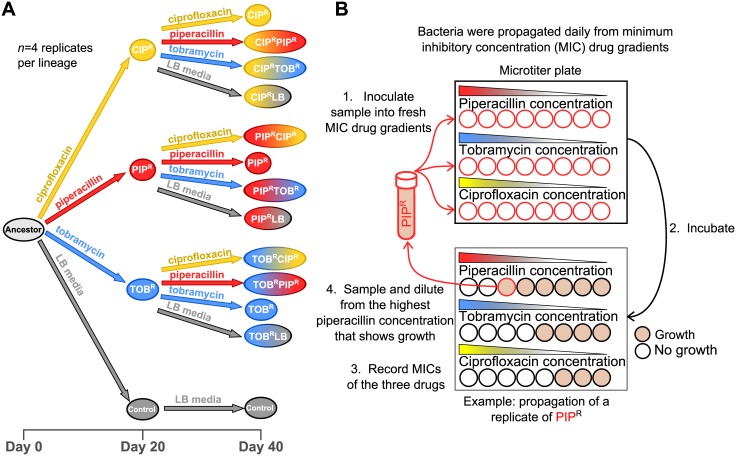
Adaptive evolution of *P*. *aeruginosa* to 3 antibiotics. (A) Ancestral *P*. *aeruginosa* PA14 was evolved daily for 20 days to piperacillin (PIP), tobramycin (TOB), ciprofloxacin (CIP), and lysogeny broth (LB) media. In the following 20 days, the 1-drug–resistant lineages were passaged further to the first drug, as well as subpassaged to the other 2 drugs, and to LB media. (B) Bacteria were taken from the highest concentration that allowed growth (defined as OD_600_ > 0.1), diluted in fresh LB, and inoculated into fresh minimum inhibitory concentration (MIC) gradients, corresponding to a daily dilution factor of 1/500. After overnight incubation, the process is then repeated.

To test how different antibiotic-resistance backgrounds affect the subsequent adaptation dynamics when evolved to a new antibiotic, we used a laboratory evolution approach to evolve *P*. *aeruginosa* to all 2-drug sequences of the 3 clinically relevant drugs piperacillin (PIP), tobramycin (TOB), and ciprofloxacin (CIP). In each of the experimental sequences, *P*. *aeruginosa* was subjected to 20 days of adaptation to each drug by serially passaging parallel replicate cultures to increasing concentrations of the drugs followed subsequently by 20 more days of adaptation to each of the 3 drugs or to lysogeny broth (LB) media without a drug (Fig 1A). Additional parallel replicates were adapted to LB media without a drug for 40 days as a control. For each drug treatment, changes in the resistance to the other 2 drugs were concurrently measured (Fig 1B). Minimum inhibitory concentration (MIC) gradients in microtiter plates were used to simultaneously measure the drug resistance level and to propagate the bacteria daily. To adapt the bacteria to a drug, a sample is taken from the population from the well of the highest drug concentration that allowed for growth (i.e., MIC/2) and then used to inoculate a new MIC gradient. This serial dilution cycle is done daily. More explicitly, 20 μl of culture is sampled from the well of the highest concentration that allowed for growth, then diluted in 5 ml of fresh LB media, and then this diluted culture is used to inoculate a new MIC gradient. This dilution protocol results in a daily dilution factor of the bacterial population of 1/500 (Materials and methods, Fig 1B). S1 Data provides the estimated number of generations per day for the evolved lineages based on the daily measurements of the OD_600_. For each lineage, the OD_600_ values are fairly consistent from day to day, and so with a dilution factor of 1/500, the cultures undergo approximately 9 generations of growth per daily dilution cycle (S1 Data).

We observed differences in final resistance levels to the different drugs depending on the history of past treatments (or lack of treatments), an effect we call drug-order–specific effects of adaptation. Our results show that a history of past drug adaptation can affect the rate at which resistance can potentially arise when subsequently adapted to a new antibiotic. Furthermore, in some cases, adaptation to a second drug or to LB can partially or fully restore sensitivity to the first drug. These observations suggest that in order to limit the rate of development of antibiotic resistance, it is important to consider which drugs a bacterial population may have been exposed to in the past when choosing which drugs to subsequently deploy.

The 3 drugs tested have different mechanisms of action and are clinically used to treat *P*. *aeruginosa* infections [29]. Piperacillin is a beta-lactam that inhibits cell wall synthesis [30]; tobramycin is an aminoglycoside that binds to the prokaryote ribosome and inhibits protein synthesis [31]; and ciprofloxacin is a fluoroquinolone that binds DNA gyrase and inhibits DNA synthesis [32]. We chose to study these 3 antibiotics because of their common use in the clinical setting to treat *P*. *aeruginosa* infections [29], their diverse mechanisms of action, and their well-studied resistance mechanisms [33]. Adaptive evolution for 20 days to these drugs individually resulted in 1-drug–resistant mutants denoted as PIP^R^, TOB^R^, and CIP^R^. The Day 20 PIP^R^, TOB^R^, and CIP^R^ populations had averages of 32-, 64-, and 64 times higher MICs to piperacillin, tobramycin, and ciprofloxacin, respectively, compared to their initial levels. To determine if the population MICs that were measured during the course of the adaptive laboratory evolution experiments were representative of individual colony MICs, we retrospectively measured the MICs of cultures grown from multiple revived colonies from the saved frozen stocks (S2 Data). Overall, 73% of the retrospectively measured MICs were within one 2-fold dilution step of the originally measured population MICs, which suggests that the reported MIC values for each of the evolved lineages are well representative of the bacterial populations (S1 Fig).

**Fig 2:**
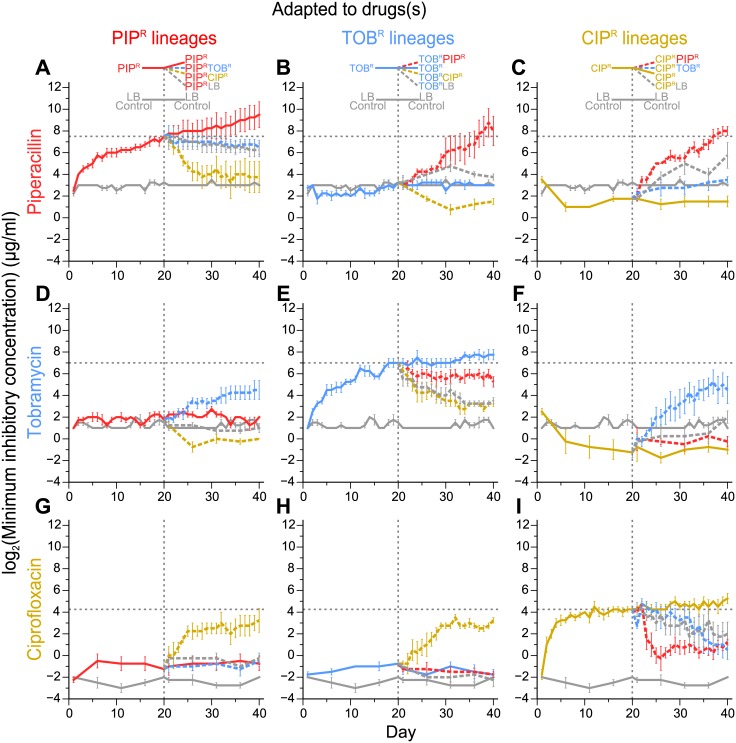
Minimum inhibitory concentration (MIC) time courses of adaptive evolution. Plots show the MICs of the treatments to the 3 drugs and lysogeny broth (LB) over time. The top (A, B, C), middle (D, E, F), and bottom (G, H, I) rows show the MICs to piperacillin (PIP), tobramycin (TOB), and ciprofloxacin (CIP), respectively. The first, second, and third columns show the MICs of the PIP^R^, TOB^R^, and CIP^R^ lineages, respectively. The dotted black lines mark the Day 20 MICs of the 3 drugs (i.e., MIC_PIP_ of Day 20 PIP^R^ in (A), MIC_TOB_ of Day 20 TOB^R^ in (E), and MIC_CIP_ of Day 20 CIP^R^ in (I)). Error bars show SEM of 4 replicates per treatment. See S3 Data for the raw data.

**Fig 3:**
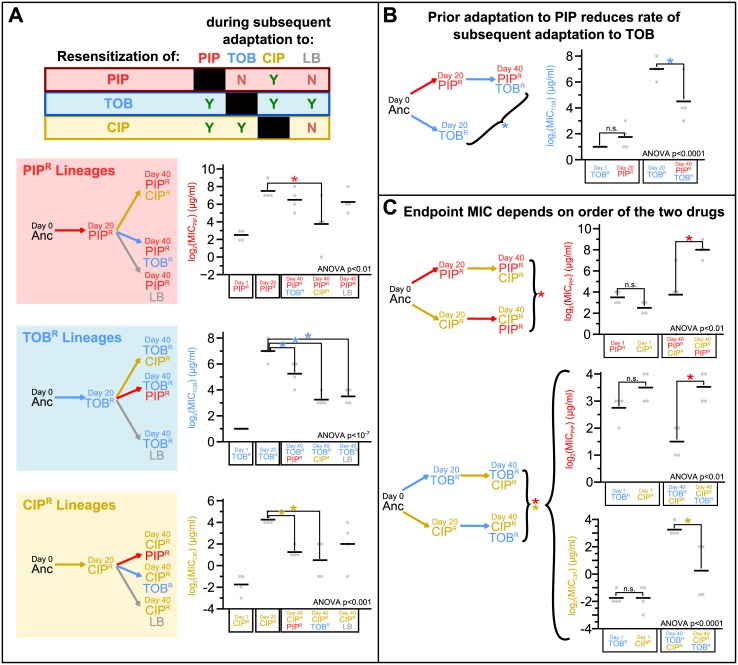
Summary of the drug-order–specific effects. (A) The Day 20 PIP^R^, TOB^R^, and CIP^R^ lineages were partially or fully resensitized to piperacillin (PIP), tobramycin (TOB), and ciprofloxacin (CIP), respectively, during subsequent adaptation to the other 2 drugs and/or lysogeny broth (LB). For example, in the top plot, a one-way ANOVA of the data shown yielded *p* < 0.01, suggesting that the mean MIC_PIP_ values of the 5 PIP^R^ lineages being compared are different. A post-hoc Tukey’s honest significant difference (HSD) test then shows that the MIC_PIP_ of Day 40 PIP^R^CIP^R^ is significantly less than that of Day 20 PIP^R^ (*p* < 0.05), indicating that resensitization to piperacillin occurred after subsequent ciprofloxacin adaptation but not after tobramycin (*p* = 0.90) or LB (*p* = 0.80) adaptation. Similar types of comparisons are done for the TOB^R^ and CIP^R^ lineages in the middle and bottom plots, and we observed that not every type of subsequent adaptation led to resensitization. The table above the plots summarizes which subsequent adaptations (columns) led to the resensitization of the 3 drugs in their respective lineages (rows). (B) The MIC_TOB_ of Day 40 PIP^R^TOB^R^ was less than that of Day 20 TOB^R^ (*p* < 0.05, Tukey’s HSD test), while the MIC_TOB_ of Day 1 TOB^R^ and Day 20 PIP^R^ were comparable. This suggests that the rate of tobramycin adaptation when there is a prior history of piperacillin adaptation is less than the rate of tobramycin adaptation when starting from the ancestor. (C) When bacteria are adapted to 2 drugs, the order of adaptation to those 2 drugs can lead to differences in the endpoint minimum inhibitory concentrations (MICs). For example, in the first plot, adaptation to ciprofloxacin followed by piperacillin led to a higher final MIC_PIP_ than the reverse order (MIC_PIP_ of Day 40 CIP^R^PIP^R^ versus Day 40 PIP^R^CIP^R^, *p* < 0.05) when they had initially comparable MIC values (MIC_PIP_ of Day 1 PIP^R^ versus Day 1 CIP^R^). The second and third plots show how the final levels of MIC_PIP_ and MIC_CIP_ are different when tobramycin adaptation is followed by ciprofloxacin adaptation or with the reverse order. For all 3 panels, the asterisks denote *p* < 0.05 (Tukey’s HSD test), not significant (n.s.) denotes *p* > 0.05, and the color of the asterisks denotes which drug MIC is being compared. In the plots, for each lineage being shown, the black bar denotes the mean of the 4 individual replicate values (gray dots). See S3 Data for the raw data and S2 Text for the calculations of the statistical tests.

By following how the resistance to each of the 3 drugs changes for each of the drug sequences (Fig 2; S2 and S3 Figs and S3 Data), we observed 3 types of drug-order–specific effects in the MIC profiles (Fig 3). Note that for now, we focus on summarizing the different drug-order–specific effects (as seen by the changes in drug MICs), and later, we discuss several hypotheses for the underlying mechanisms of the drug-order–specific effects based on analysis of the genomic mutations of the adapted lineages. In the first type of drug-order–specific effects, adaptation to a second drug or to LB restores the susceptibility to the first drug (Fig 3A). In these experiments, we were first interested to see if the increases in MICs of the 1-drug–resistant lineages (Day 20 PIP^R^, TOB^R^, and CIP^R^) were permanent or transient. By evolving them to LB and hence removing the selection pressure of the drug for 20 days, we observed that the high MIC_PIP_ was maintained in Day 40 PIP^R^LB (Fig 3A [top], *p* = 0.80; Fig 2A), while MIC_TOB_ declines (leading to partial resensitization) in Day 40 TOB^R^LB (Fig 3A [middle], *p* < 0.0001; Fig 2E), and MIC_CIP_ declines (although not significantly) in Day 40 CIP^R^LB (Fig 3A [bottom], *p* = 0.18; Fig 2I). Thus, for these 3 treatments, removal of the antibiotic pressure can maintain the high resistance or lead to resensitization in a drug-specific manner. Similar trends were seen in a recent adaptive evolution study whereby *P*. *aeruginosa* was evolved to tobramycin, ciprofloxacin, piperacillin/tazobactam, meropenem, and ceftazidime, followed by subsequent adaptation in the absence of the drug (growth medium only) to determine the effects of removing the drug selection pressure [34]. Similar to the patterns seen in our study, they observed that the tobramycin-resistant cultures partially resensitized, the ciprofloxacin-resistant cultures had a modest resensitization, and the 3 beta-lactam–evolved cultures maintained high levels of resistance.

Next, we were interested to see if evolving the 1-drug–resistant lineages to the other 2 drugs would show the same patterns seen as when evolved to LB. Interestingly, we saw unique outcomes for each of the 3 lineages. When Day 20 PIP^R^ was evolved to tobramycin, the MIC_PIP_ of Day 40 PIP^R^TOB^R^ remained high (*p* = 0.90), similar to how the MIC_PIP_ of Day 40 PIP^R^LB remained high (Fig 3A [top]). This result suggests that subsequent tobramycin adaptation has no role in altering the high piperacillin resistance. This specific order of drug treatments can then result in multidrug-resistant *P*. *aeruginosa* cultures that are resistant to both piperacillin and tobramycin. On the other hand, when Day 20 PIP^R^ was evolved to ciprofloxacin, the resulting cultures became resensitized to piperacillin (Fig 3A [top], *p* < 0.05), and the MIC_PIP_ declined to levels comparable to those of the initially susceptible cultures (MIC_PIP_ of Day 1 PIP^R^ versus Day 40 PIP^R^CIP^R^, *p* = 0.80), indicative of a full resensitization. Since resensitization did not occur after subsequent adaptation to tobramycin or LB, we suspect that the subsequent ciprofloxacin adaptation had an active role in the resensitization to piperacillin in such a way that tobramycin and LB did not. These results show that if a piperacillin-resistant culture (that is also sensitive to tobramycin and ciprofloxacin) is evolved to tobramycin, multidrug resistance can occur. However, if it is evolved to ciprofloxacin, despite the fact that ciprofloxacin resistance increases, the culture becomes susceptible to piperacillin again, making piperacillin a potentially rational choice for further treatment.

When Day 20 TOB^R^ was evolved to ciprofloxacin, partial resensitization occurred (MIC_TOB_ of Day 20 TOB^R^ versus Day 40 TOB^R^CIP^R^, *p* < 10^−5^), and the MIC_TOB_ of Day 40 TOB^R^-CIP^R^ fell to a comparable level as that of Day 40 TOB^R^LB (*p* = 0.98) (Fig 3A [middle]). This result suggests that the resensitization seen during the subsequent ciprofloxacin adaptation is not caused by the selection pressure of ciprofloxacin, but rather by the absence of the selection pressure of tobramycin. On the other hand, evolving Day 20 TOB^R^ to piperacillin also led to a partial resensitization (MIC_TOB_ of Day 20 TOB^R^ versus Day 40 TOB^R^PIP^R^, *p* < 0.05) but not as much as it did when Day 20 TOB^R^ was evolved to ciprofloxacin (MIC_TOB_ of Day 40 TOB^R^PIP^R^ versus Day 40 TOB^R^CIP^R^, *p* < 0.01) and LB (MIC_TOB_ of Day 40 TOB^R^PIP^R^ versus Day 40 TOB^R^LB, *p* < 0.05). Because of this difference, we suspect that the maintenance of the comparably high tobramycin resistance is a consequence of the piperacillin selection pressure, since we observed that adaptation in the absence of the drug pressure in LB led to substantially greater resensitization. This case highlights how the removal of **all** drug pressures may lead to the resensitization of the culture more than with the treatment of the culture to a new drug. In conjunction with the result that subsequent tobramycin adaptation of Day 20 PIP^R^ still maintained a high MIC_PIP_, this case then also shows how regardless of the order, sequential adaptation to piperacillin and tobramycin leads to multidrug resistance of the 2 drugs.

Lastly, when Day 20 CIP^R^ was evolved to piperacillin and tobramycin, both treatments lead to a partial resensitization to ciprofloxacin (Fig 3A [bottom]). During subsequent tobramycin adaptation, the decrease in the MIC_CIP_ from Day 20 CIP^R^ to Day 40 CIP^R^TOB^R^ (*p* < 0.01) was marginally more than the decrease in the MIC_CIP_ from Day 20 CIP^R^ to Day 40 CIP^R^PIP^R^ (*p* < 0.05) during subsequent piperacillin adaptation. As mentioned above, subsequent adaptation of Day 20 CIP^R^ to LB led to a decrease in MIC_CIP_ that was not statistically significant; however, we argue that the decrease is comparable to that seen when adapted to piperacillin and tobramycin as the final MIC_CIP_ of Day 40 CIP^R^LB was not significantly different than that of Day 40 CIP^R^PIP^R^ (*p* = 0.93) and that of Day 40 CIP^R^TOB^R^ (*p* = 0.53). Hence, in this case, evolution of a ciprofloxacin-resistant culture to either a different drug or to a no-drug condition led to a partial resensitization of ciprofloxacin. Interestingly, we also observed that the resensitization that occurred during subsequent piperacillin adaptation happened more quickly than the resensitization that occurred during subsequent tobramycin and LB adaptation (Fig 2I). After 5 days of subsequent piperacillin adaptation (Day 25 CIP^R^PIP^R^), the MIC_CIP_ was significantly different than that of Day 20 CIP^R^ (*p* < 0.001), while this was not the case after 5 days of subsequent tobramycin (*p* = 1.00) or LB (*p* = 0.57) adaptation. These cases in which partial or full resensitization to the first drug occurs after adaptation to a second drug or LB highlight opportunities in which resistance to 1 drug can potentially be reversed by treating with a second drug or by removing the drug pressure completely.

In the second type of drug-order–specific effects, prior adaptation to a first drug reduces the rate of subsequent adaptation to a second drug (such that the endpoint level of resistance to that second drug is lower compared to the amount of resistance developed when the Day 0 Ancestor is directly evolved to that second drug). We observed that evolution first to piperacillin reduces the rate of subsequent evolution to tobramycin (Fig 2D and 2E). That is, the MIC_TOB_ of Day 40 PIP^R^TOB^R^ was less than that of Day 20 TOB^R^ (Fig 3B, *p* < 0.05). This observation suggests that, in some cases, different bacterial populations may evolve resistance to a given antibiotic at different rates depending on the history of prior adaptations that the populations have experienced. Having knowledge of prior adaptations may then potentially be used to slow down the development of resistance to a drug if that drug is selected rationally. Interestingly, we observed no cases in which prior drug adaptation led to an enhancement in the rate of adaptation to a second drug.

The last type of drug-order–specific effects is when the final MIC of a drug is different after adaptation to a 2-drug sequence compared to after adaptation to the opposite order of the 2 drugs (Fig 3C). This third type of drug-order–specific effect exists as a consequence of a combination of the first type of effect (resensitization of the 1-drug–resistant lineages during subsequent adaptations to other drugs) and specific cases of collateral sensitivities during the adaptation of certain lineages. First, the MIC_PIP_ was higher when piperacillin was used after ciprofloxacin (Day 40 CIP^R^PIP^R^) compared to when piperacillin was used before ciprofloxacin (Day 40 PIP^R^CIP^R^) (Fig 3C [top], *p* < 0.05). In this case, adaptation to piperacillin first led to high levels of piperacillin resistance, and subsequent adaptation to ciprofloxacin led to the resensitization to piperacillin as discussed before (Fig 2A). On the other hand, even though adaptation to ciprofloxacin first led to a collateral sensitivity to piperacillin (S4A Fig [right], *p* < 0.01), subsequent adaptation to piperacillin resulted in a final MIC_PIP_ comparable to that of Day 20 PIP^R^ (Fig 2C).

Next, we observed that during the adaptation to tobramycin followed by ciprofloxacin and vice versa, the final MIC values of piperacillin and ciprofloxacin were different depending on the order of adaptation to the 2 drugs (Fig 3C [bottom and middle]). With regards to the difference seen in the final MIC_CIP_ (Fig 3C [bottom], *p* < 0.05), the partial resensitization to ciprofloxacin starting from Day 20 CIP^R^ during subsequent tobramycin adaptation (Fig 2I) resulted in the MIC_CIP_ to be less than adaptation to tobramycin first, followed by ciprofloxacin (Fig 2H). Finally, it was interesting that even though piperacillin was not the direct selection pressure, there was a difference in the final MIC_PIP_ level whether ciprofloxacin adaptation occurred after tobramycin adaptation or vice versa (Fig 3C [middle], *p* < 0.01). In this case, initial adaptation to tobramycin first did not affect the MIC_PIP_ (Fig 2B), but subsequent adaptation to ciprofloxacin resulted in a collateral sensitivity to piperacillin (S4C Fig, *p* < 0.01). On the other hand, as previously mentioned, adaptation to ciprofloxacin first initially resulted in the collateral sensitivity to piperacillin (S4A Fig [right], *p* < 0.01); however, the MIC_PIP_ returned to baseline values during subsequent adaptation to tobramycin (Fig 2C). Thus, regardless if ciprofloxacin adaptation occurred before or after tobramycin adaptation, ciprofloxacin adaptation led to piperacillin collateral sensitivity. However, in order to take advantage of this collateral sensitivity, ciprofloxacin adaptation should be used after tobramycin adaptation, rather than vice versa. In a contrasting example, we also found it interesting that while ciprofloxacin adaptation also led to collateral sensitivity of tobramycin, subsequent piperacillin adaptation did not cause the MIC_TOB_ to return to baseline levels (Fig 2F) in the manner in which subsequent tobramycin adaptation returned the MIC_PIP_ to baseline values (Fig 2C). Altogether, these cases highlight how treating an infection with a sequence of 2 drugs can result in different resistance profiles depending on the order used.

All the cases of collateral sensitivity that were observed occurred during ciprofloxacin treatment whereby ciprofloxacin adaptation resulted in a lower MIC of piperacillin or tobramycin compared to baseline levels (S4 Fig). First, adaptation to ciprofloxacin starting from the Day 0 Ancestor resulted in collateral sensitivity to both piperacillin (Fig 2C; S4A Fig [right], *p* < 0.01) and tobramycin (Fig 2F; S4A Fig [left], *p* < 0.0001). Next, adaptation to ciprofloxacin starting from both the 1-drug–evolved lineages Day 20 PIP^R^ (Fig 2D) and Day 20 TOB^R^ (Fig 2B) resulted in collateral sensitivity to tobramycin (S4B Fig, *p* < 0.01) and piperacillin (S4C Fig, *p* < 0.01), respectively. These results suggest that regardless of historical background, ciprofloxacin adaptation results in collateral sensitivity to the other 2 drugs. While we observed that collateral sensitivity of other drugs occurs only during ciprofloxacin adaptation, a recent study in which *P*. *aeruginosa* ATCC 27853 was evolved to different antibiotics reported that evolution to tobramycin resulted in collateral sensitivity to piperacillin-tazobactam and ciprofloxacin, whereas we did not observe this effect [34]. Also, this study did not observe that adaptation to ciprofloxacin resulted in collateral sensitivity to piperacillin and tobramycin, as we reported here. We suspect that these inconsistences may be due to strain-specific differences in the different *P*. *aeruginosa* strains used (strain PA14 was used in this study).

We were interested in measuring the fitnesses of the evolved lineages to see if the adaptations to the different treatments altered their growth dynamics. We measured the growth curves (OD_600_) of the 68 evolved replicate lineages as well as the Day 0 Ancestor in quadruplicate grown in LB for 24 hours (S5 Fig). The exponential growth rates were subsequently calculated from the growth curves (S6A Fig, S1 Text and S4 Data). While we observed many different growth rates across the different lineages, we did not observe any correlation between the growth rate and the change in MIC between the Day 20 1-drug–evolved lineages and the subsequent Day 40 lineages (i.e., altered growth rates could not explain the cases in which subsequent adaptation led to the maintenance of high resistance or resensitization to the first drug) (S6B Fig and S1 Text).

### Genomic mutations of adapted lineages

**Fig 4:**
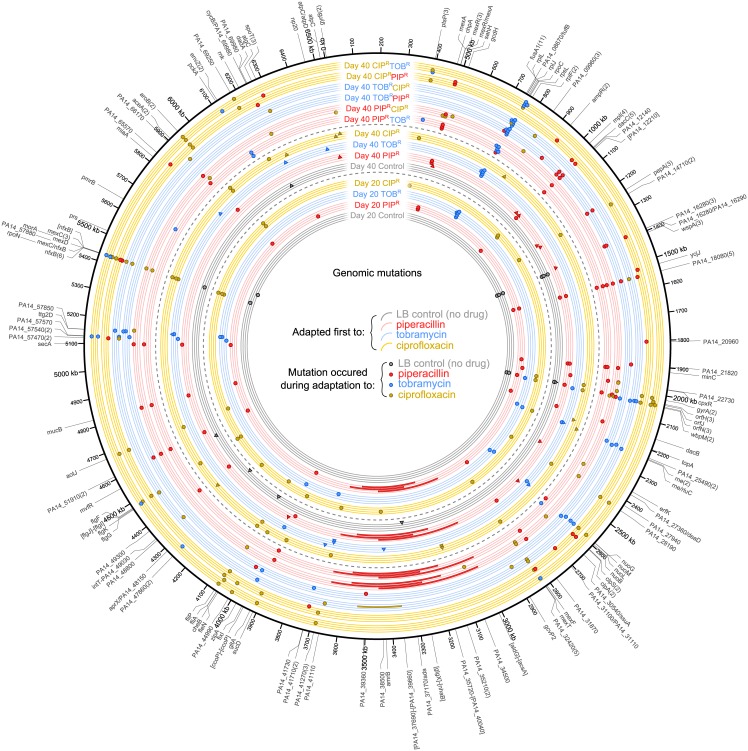
Genomic mutations of the evolved lineages. Mutations for the Day 20 and Day 40 mutants are plotted according to position on the chromosome. Each lineage is labeled and has 4 tracks for the 4 replicates per treatment. The inner set of tracks are the Day 20 1-drug–evolved lineages, the middle set of tracks are the Day 40 1-drug–evolved lineages, and the outer set of tracks are the Day 40 2-drug-evolved lineages. The color of the track denotes the treatment during the first 20 days. The color of the plotted mutation denotes during which treatment the mutation occurred. For example, a blue dot on a yellow track denotes a mutation in a Day 40 CIP^R^TOB^R^ replicate lineage that occurred during tobramycin adaptation (i.e., between Day 21 and Day 40). For the Day 40 1-drug–evolved lineages, circles denote mutations that occurred during the first set of 20 days, and triangles denote mutations that occurred during the second set of 20 days. Large rectangles denote large genomic deletions. Numbers in parentheses next to gene names indicate the number of unique mutations that occurred in that gene.

We hypothesized that genomic mutations acquired during adaptive evolution contributed to the drug-order–specific effects observed in the MIC profiles. We sequenced genomes of the Day 0 Ancestor, Day 20 PIP^R^, TOB^R^, CIP^R^, and LB Control lineages and the Day 40 1-drug–and 2-drug–evolved lineages, as well as the LB Control lineages. Genome sequencing of the Day 20 and Day 40 mutants revealed a total of 201 unique mutations across the 56 samples consisting of 77 SNPs, 31 insertions, and 93 deletions (Fig 4; S7 Fig, S1 and S2 Tables). The 77 SNPs were found within 49 genes. Two SNPs were synonymous, and 6 were intergenic. To test how representative the sequencing results were of the mutant populations, we used PCR and Sanger sequencing to test for the presence of specific mutations in multiple colonies of different lineages after reviving the lineages from the saved frozen samples. We used the primers from S3 Table to test for the presence of 1 mutation from 1 replicate of each lineage, with 4 colonies of each lineage. Overall, while there may be limited heterogeneity in the populations with respect to a few of the mutations, the large majority of the mutations were homogeneous in the populations and fixed within the lineages (S1 Text).

While some genes were mutated during evolution to all drugs, other mutations were drug-specific and were related to the drugs’ primary mechanisms of action as would be expected (S4 Table). Genes encoding transcriptional regulators for multidrug efflux pumps were commonly mutated during evolution to all 3 drugs (*mexC*, *mexR*, *mexS*, *nalC*, *nalD*, *nfxB*, *parS*) [35]. Ribosomal proteins (*rplJ*, *rplL*, *rpsL*, *rplF*) [36] and NADH dehydrogenase subunits (*nuoB*, *nuoG*, *nuoL*, *and nuoM*) [37,38] were frequently mutated during tobramycin evolution. The most commonly mutated gene was *fusA1*, which encodes elongation factor G and was mutated in 11 different replicate lineages adapted to tobramycin. *fusA1* has been observed to be mutated in clinical isolates of *P*. *aeruginosa* [39–41], as well as in adaptive evolution studies to aminoglycosides in *P*. *aeruginosa* [34] and *E*. *coli* [12,16,18]. Mutations in *fusA1* may also contribute to altered intracellular (p)ppGpp levels, which may modulate virulence in *P*. *aeruginosa* [41]. Mutations in *gyrA* and *gyrB* were observed during ciprofloxacin evolution, but none were observed in *parC* and *parE* (the other genes of the quinolone resistance-determining region [29]). Lastly, genes encoding peptidoglycan synthesis enzymes (*dacC*, *mpl*) and beta-lactamase regulators (*ampR*) were mutated during piperacillin treatment. Many of these genes have also been observed to be mutated during human host adaptation of *P*. *aeruginosa* [42], highlighting the importance of several of these clinical resistance determinants (S1 Text).

**Fig 5:**
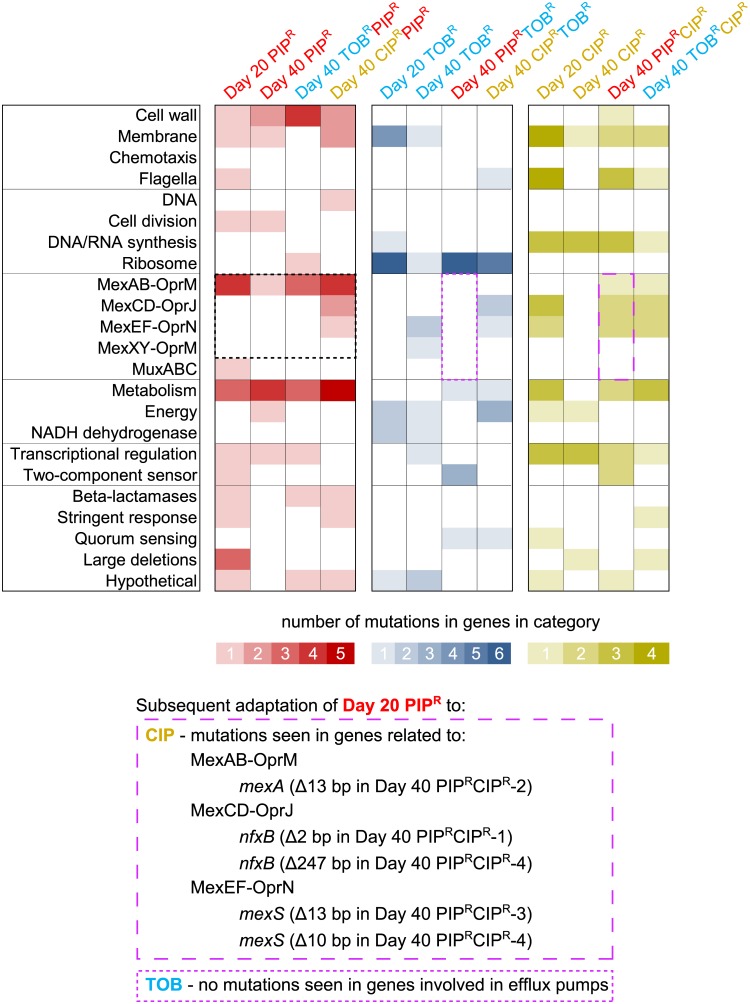
The frequency of mutated genes during piperacillin (PIP), tobramycin (TOB), and ciprofloxacin (CIP) adaptation depending on the historical background. The number of unique mutations observed in a gene in a functional class (rows) is shown based on the intensity of the color across all 4 biological replicates for each of the lineages (columns). The lineages are grouped according to the final (or only) drug that the lineage was adapted to in order to compare how historical context affects how often genes in the functional classes are mutated. For example, the first 4 columns (with red shading) correspond to the frequency of genes mutated in the lineages that were adapted to piperacillin only (Day 20 PIP^R^ and Day 40 PIP^R^) and piperacillin after prior adaptation to a first drug (Day 40 TOB^R^PIP^R^ and Day 40 CIP^R^PIP^R^). Note that the data in the Day 40 PIP^R^ column correspond to additional mutations that occurred (between Day 21 and 40) and do not double count the ones from Day 20 PIP^R^ column. As an example of how different genes are mutated during piperacillin adaptation under different historical contexts, the cells outlined by the dashed-black box show that regardless of historical context, all lineages that underwent piperacillin adaptation had mutations in genes related to the MexAB-OprM efflux pump. However, only the lineage that had prior ciprofloxacin adaptation (Day 40 CIP^R^PIP^R^) had mutations in genes related to the MexCD-OprJ and MexEF-OprN efflux pumps. Lastly, none of the piperacillin-adapted lineages had mutations in genes involved in the MexXY-OprM efflux pump. The cells outlined by the dashed-purple boxes show that while subsequent adaptation of Day 20 PIP^R^ to ciprofloxacin (Day 40 PIP^R^CIP^R^) resulted in several mutations in genes involved in efflux pumps, subsequent adaptation to tobramycin (Day 40 PIP^R^TOB^R^) resulted in no mutations in genes involved in efflux pumps. The corresponding mutations that occurred are explicitly listed at the bottom. See the main text for more details of how this difference may play a role in the resensitization to piperacillin during subsequent ciprofloxacin adaptation of Day 20 PIP^R^. See S1 Table for the complete list of mutations and S2 Table for descriptions of the mutated genes.

We next analyzed the genomic mutations to see how the historical context affects which mutations occur during adaptation to a drug. For example, how do the mutations that occur during adaptation to piperacillin only (Day 20 PIP^R^ and Day 40 PIP^R^) compare to the mutations that occur during piperacillin adaptation when there is a prior history of adaptation first to tobramycin (Day 40 TOB^R^PIP^R^) or ciprofloxacin (Day 40 CIP^R^PIP^R^)? To this end, we first categorized the genes in which mutations occurred into 23 broad categories based on the available literature and on the PseudoCAP functional classifications from the Pseudomonas Genome Database [43] (Table 1). Next, for each lineage, we tallied the number of times a gene in a functional category was mutated across the 4 biological replicates for each of the lineages (Fig 5). For a complete list of genes in each functional classification and descriptions of the genes, see S2 Table.

**Table 1:** Functional classifications of the mutated genes.

Cell wall	*dacC*, *mpl*
Membrane	*algC*, *aotJ*, *fixI*, *nppA1*, *secA*, *wbpM*, *ycjJ*, [PA14_12210], PA14_25490, PA14_30540/*ssuA*, PA14_34500, PA14_41710, PA14_48800, PA14_57880
Chemotaxis	*chpA*
Flagella	[*flgJ*]–[*flgI*], *cheB*, *fleN*, *flgF*, *flgG*, *flgK*, *fliA*, *fliP*, *morA*, *orfH*, *orfJ*, *orfN*, *wspA*
DNA	PA14_31100/PA14_31110
Cell division	*minC*, *zipA*
DNA/RNA synthesis	*gyrA*, *gyrB*, *rne*, *rpoC*, *rpoN*, *topA*, tRNA-Val
Ribosome	*fusA1*, *miaA*, *rne/rluC*, *rplF*, *rplJ*, *rplL*, *rpsL*, tRNA-Thr/*tufB*
MexAB-OprM	*mexA*, *mexR*, *mexR/mexA*, *nalC*, *nalD*, *nalC*/PA14_16290
MexCD-OprJ	[*nfxB*], *nfxB*, *mexC*, *mexC/nfxB*, *mexD*
MexEF-OprN	*parS*, *mexF*, *mexS*, *mexT*
MexXY-OprM	*amrB*
MuxABC	*muxA*
Metabolism	*aceA*, *aroB*, *clpA*, *clpS*, *dadA*, *gcdH*, *gcvP2*, *gltA*, *lhpE*, *pepA*, *prs*, *sahH*, PA14_20960, PA14_21820, PA14_27360/*deaD*, PA14_49300, PA14_57470, PA14_66170
Energy	[*ccoP*]-[*ccoP*], *atpC*, *atpC/atpD*, *cycB/pauR*, *pckA*, *sucD*, PA14_57540, PA14_57570
NADH dehydrogenase	*nuoB*, *nuoG*, *nuoL*, *nuoM*
Transcriptional regulation	*iscR*, *mucB*, *mvfR*, *np20*, *pauR*, *rnk*, PA14_09960, PA14_12140, PA14_35210, PA14_37170/*ada*, PA14_38500, PA14_39360
2-component sensor	*envZ*, *cpxR*, *pmrB*, PA14_22730, PA14_27940
Beta-lactamases	*ampR*, *dacB*
Stringent response	*spoT*
Quorum sensing	*ptsP*
Large deletions	[*aldG*]–[*acsA*], [*glgX*]–[*nhaB*], *intT*–PA14_49030, PA14_35720–[PA14_40040], [PA14_37690]–[PA14_39660]
Hypothetical	*aprX*/PA14_48150, *erfK*, *ttg2D*, PA14_41730, PA14_44990, PA14_51910, PA14_57850, PA14_65570, PA14_69250

Brackets (e.g., [*gene*]) denote deletion of more than a few base pairs within a gene. Forward slashes (e.g., *gene1*/*gene2*) denote mutations in the intergenic region between the 2 genes. Hyphens (e.g. *gene1*—*gene2*) denote deletions spanning multiple genes. For a complete list of genes in each functional classification and descriptions of the genes, see S2 Table.

We observed several general trends in the genes mutated during adaptation to the 3 drugs, depending on their historical context. In the lineages adapted to piperacillin, we saw history-dependent trends in the mutated genes that were related to multidrug efflux pumps (Fig 5, dashed-black box). While all the piperacillin-adapted lineages had mutations in genes related to the MexAB-OprM efflux pump (which is the primary efflux pump of piperacillin [44]) such as *nalD* and *mexR* (whose products repress the expression of *mexAB-oprM* [45]), the Day 40 CIP^R^PIP^R^ lineage had additional mutations in the structural subunit genes of the other efflux pumps MexCD-OprJ (*mexC*) and MexEF-OprN (*mexF*). Lastly, no mutations in genes related to the MexXY-OprM pump were observed in any of the piperacillin-adapted lineages. With regard to adaptation to piperacillin only, most of the mutations that occurred in genes related to MexAB-OprM occurred within the first 20 days, with only a few additional mutations occurring between Day 21 and 40. Regardless of historical context, metabolic and cell wall genes tended to be frequently mutated in piperacillin-adapted lineages, whereas metabolic and cell wall genes did not seem to be consistently mutated across the tobramycin-adapted and ciprofloxacin-adapted lineages. This result is perhaps due to the fact that the primary target of piperacillin is cell wall (peptidoglycan) synthesis, which is largely a metabolic process. Interestingly, we also observed that the lineages adapted only to piperacillin (Day 20 PIP^R^) sustained large chromosomal deletions that were not seen in the lineages in which there was prior tobramycin or ciprofloxacin adaptation (Day 40 TOB^R^PIP^R^ and Day 40 CIP^R^PIP^R^). We discuss and explore the potential implications of these large deletions below.

The tobramycin-adapted lineages consistently had mutations occur in ribosomal subunit genes and other ribosomal machinery genes, regardless of historical context. In the lineages adapted only to tobramycin, mutations in genes related to the ribosome, membrane, energy, and NADH dehydrogenase tended to occur by Day 20, followed by mutations in efflux pump–related genes by Day 40. The mutations in genes related to membrane, NADH dehydrogenase, and energy likely reflect the unique requirement of the proton-motive force for the uptake of aminoglycoside antibiotics [46], and the mutations occurring during tobramycin adaptation may contribute to the resistance by reducing the proton-motive force [16]. While we observed mutations in the NADH dehydrogenase genes in the lineages adapted only to tobramycin, we saw no such mutations in the lineages in which prior piperacillin or ciprofloxacin adaptation occurred (Day 40 PIP^R^TOB^R^ and Day 40 CIP^R^TOB^R^). Also, while efflux pump–related genes were mutated in the Day 40 TOB^R^ and Day 40 CIP^R^TOB^R^ lineages, no such mutations were seen in the Day 40 PIP^R^TOB^R^ lineages in which prior adaptation to piperacillin occurred (Fig 5, dashed-purple boxes).

The mutations in the ciprofloxacin-adapted lineages were fairly consistently distributed regardless of historical context. For all ciprofloxacin-adapted lineages, mutations were seen in genes related to DNA/RNA synthesis as expected, as well as in genes related to membrane, flagella, efflux pumps, metabolism, and transcriptional regulators. Mutations related to the MexAB-OprM, MexCD-OprJ, and MexEF-OprN efflux pumps (mostly in genes encoding negative regulators of the pumps) are seen in the ciprofloxacin-adapted lineages, reflecting the ability of these different pumps to extrude ciprofloxacin; however, no mutations were seen in genes related to MexXY-OprM, even though this pump is also known to contribute to fluoroquinolone resistance [44]. Further experiments in measuring the gene expression of the different efflux pumps may help elucidate the roles that these pumps play in contributing to the different drug-order–specific effects.

Next, we sought to determine if the patterns in mutated genes could explain the mechanisms of some of the drug-order–specific effects that were observed in the MIC time courses. We first discuss the cases of resensitization or maintenance of high resistance in which the 1-drug–evolved lineages were subsequently adapted to the other 2 drugs or to LB (Fig 3A). While subsequent adaptation of Day 20 PIP^R^ to LB and tobramycin maintained high piperacillin resistance, subsequent adaptation to ciprofloxacin led to full resensitization to piperacillin (Fig 3A [top]). We hypothesize that these differences stem from the different efflux pump-related genes that were mutated in these lineages (Fig 5, dashed-purple boxes). Evolution of the Day 0 Ancestor to piperacillin resulted in 2 different SNPs in *nalD*, and 1 SNP in *mexR* across the 4 biological replicates of Day 20 PIP^R^, likely leading to the overexpression of the MexAB-OprM efflux pump [45]. We suspect that MIC_PIP_ remained high during subsequent adaptation to LB and tobramycin due to continued overexpression of MexAB-OprM.

However, when Day 20 PIP^R^ was adapted to ciprofloxacin, several mutations occurred in genes related to other efflux pumps, including 1 in *mexA*, 2 in *nfxB*, and 2 in *mexS* (Fig 5, dashed-purple boxes). In particular, *mexS* encodes a negative regulator of the expression of MexEF-OprN, and mutations in this gene likely lead to the overexpression of the efflux pump [47]. Interestingly, expression of MexEF-OprN has been observed to correlate inversely with the expression of MexAB-OprM [47,48]. Hence, we suspect that the resensitization to piperacillin when Day 20 PIP^R^ was subsequently adapted to ciprofloxacin may be have been due to a concurrent decrease in MexAB-OprM expression (leading to reduced piperacillin efflux) as MexEF-OprN expression increased. That is, it is possible that the mutations that occurred during ciprofloxacin adaptation that led to the overexpression of MexEF-OprN negated the effects of the mutations that occurred during prior piperacillin adaptation that led to overexpression of MexAB-OprM. Furthermore, we observed no mutations in efflux pump–related genes in Day 40 PIP^R^TOB^R^ (Fig 5, dashed-purple boxes), which supports the notion that because no mutations occurred, which would have negatively correlated with the expression of MexAB-OprM, expression of this efflux pump was maintained throughout the subsequent adaptation to tobramycin, and hence the MIC_PIP_ remained high.

We observed that subsequent adaptation of Day 20 TOB^R^ to LB and ciprofloxacin resulted in a partial resensitization to tobramycin, and that while subsequent adaptation to piperacillin also led to a significantly lower MIC_TOB_, it was not as low as that of Day 40 TOB^R^LB and TOB^R^CIP^R^ (Fig 3A [middle]). In this case, the partial resensitization during subsequent adaptation to LB may be attributable to adaptive resistance of aminoglycosides in *P*. *aeruginosa*. Adaptive resistance is a phenomenon in which resistance to a drug is transiently induced in the presence of the drug, and resistance recedes upon the removal of the drug [49]. In contrast to acquired resistance, which is mediated through genetic mutations, adaptive resistance is explained by phenotypic alterations that allow for temporary increases in resistance. *P*. *aeruginosa* is known to exhibit adaptive resistance to aminoglycosides [50,51], and it is primarily mediated through up-regulation of MexXY-OprM during drug exposure and subsequent down-regulation after the removal of the drug [52]. We suspect that the partial resensitization during subsequent ciprofloxacin adaptation is also a consequence of adaptive resistance once the tobramycin selection pressure is removed. We further speculate that during the initial adaptation to tobramycin, the increase in tobramycin resistance was a combination of adaptive resistance and acquired resistance from accumulation of the mutations as seen in Day 20 TOB^R^. Thus, the resensitization during subsequent LB and ciprofloxacin adaptation was not a full resensitization but rather a partial one, perhaps reflecting the remaining contribution of the acquired resistance. Lastly, with regards to Day 40 TOB^R^PIP^R^, it is unclear how subsequent piperacillin adaptation seemingly resulted in the maintenance of high MIC_TOB_ compared to that of Day 40 TOB^R^LB and TOB^R^CIP^R^. We hypothesize that the subsequent piperacillin adaptation somehow counteracted the resensitization effects of adaptive resistance, even when the tobramycin selection pressure was removed.

The mechanism of ciprofloxacin resensitization when Day 20 CIP^R^ was subsequently adapted to LB, piperacillin, and tobramycin remains unclear (Fig 3A [bottom]). While reversion of aminoglycoside sensitivity has been the most characterized case of adaptive resistance in *P*. *aeruginosa*, other studies have suggested that adaptive resistance may be prevalent in other classes of antibiotic classes as well, and that it may be mediated by epigenetic processes such as methylation and stochastic gene expression [53], particularly affecting the expression of efflux pumps [54]. It could be possible that adaptive resistance partially explains the resensitization to ciprofloxacin. We also note that qualitatively, there was much more variability in the MIC time courses between the individual replicates of the CIP^R^ lineages, as seen by the larger error bars in Fig 2I, compared to that of the PIP^R^ (Fig 2A) and TOB^R^ (Fig 2E) lineages. Taken together, further investigation of the partial ciprofloxacin resensitization is needed.

While we observed clear cases of collateral sensitivity develop to piperacillin and tobramycin during the course of ciprofloxacin adaptation (S4 Fig), other adaptive evolution studies of *P*. *aeruginosa* evolved to ciprofloxacin showed mixed results. In one study, the adaptation of *P*. *aeruginosa* ATCC 27853 to ciprofloxacin showed no change in the MIC of 3 different beta-lactams (including piperacillin-tazobactam), nor of tobramycin [34]. In another study, while no statistical significances were assigned, adaptation of *P*. *aeruginosa* PAO1 to ciprofloxacin appeared to result in slight collateral sensitivities to piperacillin-tazobactam and tobramycin in some of their replicates. Nevertheless, in our study, we hypothesize that the collateral sensitivity to piperacillin and tobramycin during ciprofloxacin adaptation is attributable to the mutations seen in *nfxB* (which encodes a transcriptional repressor that regulates MexCD-OprJ [55]) in the Day 20 CIP^R^ lineages. Three of the Day 20 CIP^R^ replicates had deletions in *nfxB* (15, 13, and 16 base pairs), likely resulting in the inactivation of NfxB and concomitant up-regulation of MexCD-OprJ and increased ciprofloxacin resistance [56]. In fact, *nfxB* mutants have been reported to be hypersusceptible to certain beta-lactams and aminoglycosides [57,58].

Lastly, with regards to the decreased rate of tobramycin adaptation given a history of prior piperacillin adaptation (Fig 3B), we attribute this effect to the large chromosomal deletions that were sustained in 3 of the 4 Day 20 PIP^R^ replicates. The consequences of these deletions are discussed in more detail in the subsequent sections of the manuscript. In summary, based on the genomic mutations, we have presented our interpretations of potential mechanisms that contribute to the drug-order–specific effects. These include how historical context can influence the frequency of mutations in certain genes, the varying contributions of adaptive and acquired resistance to total resistance, and specific cases of inverse correlation of the expression of different efflux pumps. While mutations are likely not the sole determinants of the differences [34,59], many of the observed genomic mutations can partially explain the drug-order–specific effects.

### Drug history dependence of pyomelanin hyperproduction

One striking mutation we observed was that 3 of the 4 replicates of Day 20 PIP^R^ (Day 20 PIP^R^-1, -2, and -3) had large, approximately 400 kbp deletions (corresponding to approximately 6% of the genome) in a conserved region of the chromosome (Fig 4 [large red rectangles]; S5 Data), suggestive of selective genome reduction [60–63], and have been associated with directed repeats [64] and inverted repeats [65] at the boundaries of the deletions. These large deletions were also fixed in the corresponding Day 40 PIP^R^TOB^R^, Day 40 PIP^R^CIP^R^, and Day 40 PIP^R^LB lineages. Interestingly, the 3 PIP^R^ lineages with these large deletions hyperproduced the brown pigment pyomelanin during piperacillin evolution, and this visually observable phenotype also persisted when evolved to tobramycin (PIP^R^TOB^R^), ciprofloxacin (PIP^R^CIP^R^), and LB (PIP^R^LB). The loss of *hmgA* as part of the large chromosomal deletions correlates exactly with the pyomelanin phenotype of these lineages. Indeed, *hmgA* mutants of *P*. *aeruginosa* hyperproduce pyomelanin [66]. This observation shows that evolving to piperacillin results in a high probability of sustaining large deletions spanning *hmgA*, which results in the pyomelanogenic phenotype. However, when we evolved the Day 20 TOB^R^ and CIP^R^ lineages to piperacillin to yield the Day 40 TOB^R^PIP^R^ and Day 40 CIP^R^PIP^R^ lineages (4 replicates each), none of them became pyomelanogenic, suggesting that prior history of tobramycin or ciprofloxacin adaptation leads to a lower propensity of becoming pyomelanogenic when subsequently evolved to piperacillin. Interestingly, 1 of the Day 20 TOB^R^ replicates became pyomelanogenic when subsequently evolved to ciprofloxacin (Day 40 TOB^R^CIP^R^-2). Hence in this study, pyomelanin hyperproduction is a consequence of piperacillin and ciprofloxacin evolution, yet the likelihood to evolve this visually striking and observable phenotype depends on the history of prior drug adaptation.

**Fig 6:**
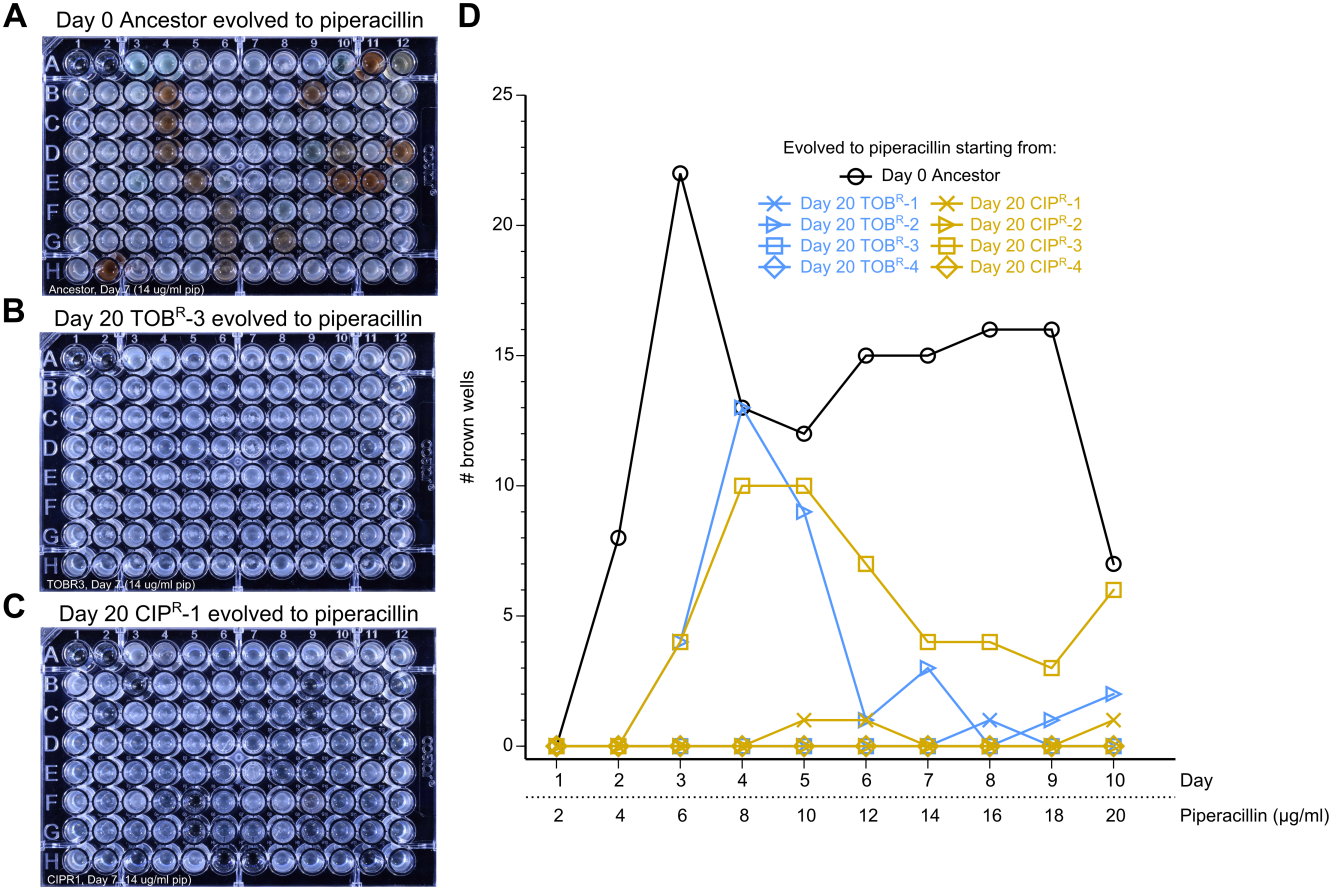
Wild-type *P*. *aeruginosa* has a higher propensity to become pyomelanogenic when evolved to piperacillin compared to tobramycin-resistant (TOB^R^) and ciprofloxacin-resistant (CIP^R^) lineages. We tested how common it was for piperacillin adaptation to lead to pyomelanin hyperproduction under different historical backgrounds. Ninety-two replicates of (A) Day 0 Ancestor, (B) Day 20 TOB^R^-3, and (C) Day 20 CIP^R^-1 were passaged daily to low, increasing concentrations of piperacillin for 10 days. Photographs of Day 7 of passaging show how the Ancestor had a higher propensity of evolving the pyomelanin phenotype during piperacillin treatment compared to evolution of Day 20 TOB^R^-3 and Day 20 CIP^R^-1. The complete set of photographs for all lineages tested is shown in S8–S10 Figs. (D) The number of visibly brown wells was tracked daily over the course of the 10 days of piperacillin evolution. Overall, Day 0 Ancestor had the highest propensity to become pyomelanogenic during piperacillin evolution, followed by Day 20 CIP^R^-3 and Day 20 TOB^R^-2. Interestingly, the number of brown wells for these lineages did not increase monotonically over time, suggesting heterogeneity in these populations, and that nonpyomelanogenic subpopulations outcompeted the pyomelanogenic ones in the wells that transiently turned brown.

While the 3 PIP^R^ lineages that produced pyomelanin were not significantly more resistant to piperacillin than the nonpyomelanogenic PIP^R^ lineage, pyomelanin-producing strains have been observed clinically [60] and have been shown to be more persistent in chronic lung infection models [66]. We tested the reproducibility of this example of a phenotypic dependence on the history of drug adaptation with a higher throughput approach. Starting with clonal populations of Day 0 Ancestor, Day 20 TOB^R^, and Day 20 CIP^R^, we seeded 92 replicate populations of each lineage into microplates, and we used a 96-pin replicating tool to serially propagate these populations and evolve them to increasing concentrations of piperacillin daily. The lineages that started from Day 0 Ancestor had the highest propensity to become pyomelanogenic (Fig 6A) compared to lineages starting from Day 20 TOB^R^ (Fig 6B) or Day 20 CIP^R^ (Fig 6C). Still, certain lineages starting from Day 20 TOB^R^ and Day 20 CIP^R^ did also produce pyomelanin, albeit with less propensity than starting from Day 0 Ancestor (Fig 6D; S8–S10 Figs).

### Drug-order–specific effects in clinical isolates

**Fig 7:**
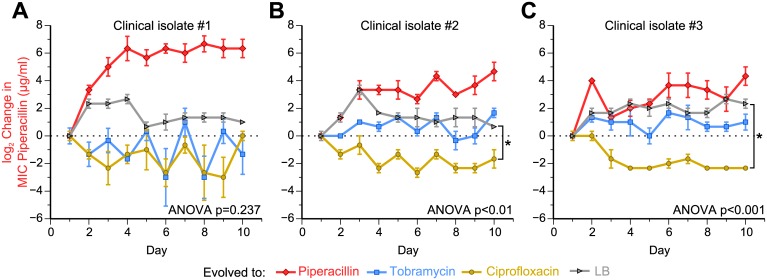
Clinical isolates with high piperacillin resistance become resensitized to piperacillin following adaptation to ciprofloxacin. To see if we could recapitulate the adaptation dynamics of the minimum inhibitory concentration of piperacillin (MIC_PIP_) when Day 20 PIP^R^ was evolved to tobramycin and ciprofloxacin, we evolved 3 piperacillin-resistant clinical isolates of *P*. *aeruginosa* to piperacillin, tobramycin, ciprofloxacin, and lysogeny broth (LB). (A) While the first isolate did not show restoration of piperacillin sensitivity during ciprofloxacin evolution as anticipated, (B and C) the other 2 isolates recapitulated this effect. In clinical isolates #2 and #3, the relative changes in the MIC_PIP_ when the isolates were evolved to ciprofloxacin were significantly different from the relative changes when evolved to LB at Day 10 (*p* < 0.05 and *p* < 0.001, respectively). For each of the 3 isolates, a one-way ANOVA was first performed on the Day 10 MIC_PIP_ values of the lineages evolved to LB, tobramycin, and ciprofloxacin. Error bars show SEM of 3 replicates per treatment. See S2 Text for the calculations of the statistical tests and S11 Fig for the original, prenormalized data.

To explore the relevance of our laboratory evolution results clinically, we tested for the drug-order–specific MIC evolutionary dynamics in clinical isolates. We first tested the evolutionary dynamics of clinical isolates that were resistant to piperacillin but susceptible to tobramycin and ciprofloxacin. We evolved 3 piperacillin-resistant clinical isolates of *P*. *aeruginosa* to piperacillin, tobramycin, and ciprofloxacin for 10 days and tracked how the piperacillin resistance changed in these lineages. If the results from the adaptive evolution experiment applied to these piperacillin-resistant clinical isolates, then we would expect that evolving to tobramycin would not affect the high piperacillin resistance, but evolving to ciprofloxacin would restore susceptibility to piperacillin. As mentioned previously, evolving Day 20 PIP^R^ to LB did not result in a reduction of MIC_PIP_, which suggests that the resensitization to piperacillin when Day 20 PIP^R^ was evolved to ciprofloxacin was a consequence of the switch to the ciprofloxacin drug pressure. Of the 3 isolates we tested, the evolutionary dynamics of 2 of these isolates matched these expectations (Fig 7; S11 Fig and S3 Data). After normalizing to Day 1 MIC values, the MIC_PIP_ after 10 days of ciprofloxacin adaptation was significantly less than the MIC_PIP_ after 10 days of LB adaptation in isolate #2 (Fig 7B, *p* < 0.05) and in isolate #3 (Fig 7C, *p* < 0.001), indicating resensitization to piperacillin during ciprofloxacin adaptation. This observation suggests that the MIC evolutionary dynamics we observed are not limited to laboratory strains of *P*. *aeruginosa* and may be observed in diverse strains of *P*. *aeruginosa*, including those originating from human patients. Note that these 3 clinical isolates were isolated from different patients, and their phylogenetic relatedness between each other and to the laboratory PA14 strain used in our study is untested. In isolate #1, there was no significant difference in the normalized MIC_PIP_ values after 10 days of adaptation to tobramycin, ciprofloxacin, and LB (Fig 7A, *p* = 0.237, one-way ANOVA). Interestingly, this isolate evolved higher levels of piperacillin and ciprofloxacin resistance than the other 2 isolates (S11 Fig and S3 Data), which suggests the possibility that adaptation to ciprofloxacin in these higher piperacillin-resistant cultures could still result in a restoration of piperacillin susceptibility.

**Fig 8:**
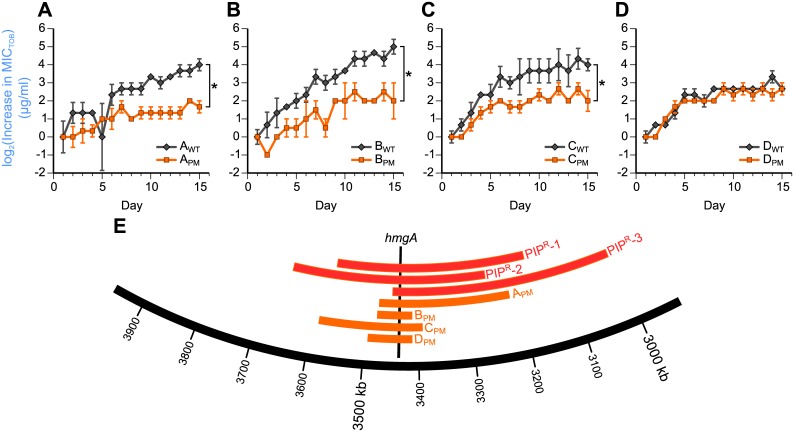
Clinical isolates with large chromosomal deletions have lower rates of tobramycin (TOB) resistance evolution. To see if large chromosomal deletions played a role in reducing the rate of tobramycin resistance evolution, 4 pairs of clinical isolates were evolved to tobramycin. Each pair consisted of a pyomelanogenic (PM) isolate with a large deletion, and its corresponding nonpyomelanogenic parental isolate that does not have a large deletion (“wild-type” [WT]) [64]. As anticipated, we observed that (A) A_PM_, (B) B_PM_, and (C) C_PM_ had lower relative increases in the minimum inhibitory concentration of tobramycin (MIC_TOB_) compared to A_WT_, B_WT_, and C_WT_, respectively. However, (D) D_WT_ and D_PM_ had comparable relative increases in MIC_TOB_. Asterisks denote *p* < 0.05 of a two-sample *t* test after the raw MIC values were normalized by subtracting the average Day 1 MIC_TOB_ for each evolved lineage. See S2 Text for the calculations of the statistical tests. Error bars show SEM of 3 replicates per treatment (except B_PM_-2, which had 2 replicates). (E) The large deletions of the 4 “PM” isolates are located in the same region as the deletions of Day 20 PIP^R^-1, -2, and -3, and all of the deletions encompass *hmgA*, whose loss causes the hyperproduction of pyomelanin. See S13 Fig for the original, prenormalized data.

In the next set of evolution experiments, we investigated the role that the large chromosomal deletions play in a drug-order–specific effect. We had observed that compared to the Day 20 PIP^R^ replicate that did not have a large deletion, the 3 Day 20 PIP^R^ replicates with the large deletions, when subsequently evolved to tobramycin, developed less tobramycin resistance (S3 Data and S12 Fig). This observation suggests that the large deletions were involved in reducing the subsequent rate of tobramycin resistance evolution given a prior history of piperacillin adaptation. A recent study isolated 4 pairs of clinical isolates of *P*. *aeruginosa*, in which each pair consisted of a pyomelanogenic (PM) isolate and a “parental wild-type (WT)” nonpyomelanogenic isolate [64]. In each of the 4 pairs, the only genomic difference between the pyomelanogenic (denoted A_PM_, B_PM_, C_PM_, and D_PM_) and its corresponding parental wild-type isolate (denoted A_WT_, B_WT_, C_WT_, and D_WT_) was the presence of large chromosomal deletions that overlap with parts of the deletions seen in Day 20 PIP^R^-1, -2, and -3 (Fig 8E; S5 Data). Indeed, all of the large deletions encompass *hmgA*, whose loss accounts for the pyomelanin phenotype. We used these 4 pairs of clinical isolates to test the hypothesis that the large deletions play a role in lowering the rate of tobramycin resistance evolution. We evolved the 4 pairs of isolates to tobramycin using the same daily serial passaging technique used throughout this study and tracked the MICs of tobramycin, piperacillin, and ciprofloxacin over the course of 15 days (Fig 8; S3 Data and S13 Fig). At the end of the 15 days, we saw that A_PM_, B_PM_, and C_PM_ had lower relative increases in MIC_TOB_, compared to A_WT_ (*p* < 0.01), B_WT_ (*p* < 0.05), and C_WT_ (*p* < 0.05), respectively (Fig 8A–8C). These data provide support for the idea that the large chromosomal deletions do indeed play a role in reducing the rate of tobramycin adaptation, and potentially even in limiting the maximum level of tobramycin resistance that can be developed comparatively. In the case of the fourth pair, we saw that D_WT_ and D_PM_ had comparable increases in MIC_TOB_ over the course of the tobramycin adaptation (Fig 8D, *p* = 1.00). It can be speculated that some combination of the presence or loss of specific genes in D_PM_ led to this evolutionary trajectory that is different from the other 3 pyomelanogenic isolates. We would also like to point out that within each pair, the “WT” and “PM” isolates vary in initial Day 1 MIC_TOB_. The B_PM_ and B_WT_ pair was the most disparate pair, as B_PM_ had a much lower MIC_TOB_ than B_WT_ (S13 Fig).

Interestingly, a recent study also observed large genomic deletions spanning *hmgA* when *P*. *aeruginosa* PAO1 was evolved to meropenem, which is another beta-lactam antibiotic [65]. These mutants were also pyomelanogenic. The large deletions in both our study as well as this recent study also span *mexX* and *mexY*, which encode portions of the efflux pump that is a significant determinant of aminoglycoside resistance [67]. The loss of these genes in the 3 PIP^R^ replicates may partially explain why subsequent tobramycin adaptation is limited compared to the replicate that did not sustain the large deletion.

## Discussion

This study presents evidence of how the evolutionary history of bacterial adaptation to antibiotics can complicate strategies for treating infections and for limiting the further development of multidrug resistance. Exposing bacteria to fluctuating environments has been shown to be a potentially good strategy for slowing down the development of resistance [12,19,68]. More broadly, mechanisms of memory and history dependence in bacterial systems are being uncovered to better understand the dynamics of bacterial survival and adaptation in changing environments [69–71]. For example, a recent study showed that the survival of *Caulobacter crescentus* in response to a high concentration of sodium chloride stress depended on the duration and timing of an earlier treatment of a moderate concentration of sodium chloride and that this effect was linked to delays in cell division, which led to cell-cycle synchronization [72]. Another study described what they call response memory, which is when a gene regulatory network continues to persist after the removal of its external inducer. The study showed that in *E*. *coli*, *lac* induction transiently continued when the environment was switched from lactose to glucose, which may be beneficial when the environment fluctuates over short timescales [73]. The results of those studies as well as the results from this study challenge the notion that bacteria respond solely to their present environment. Bacteria can encounter different stressors over time such as osmotic, oxidative, and acidic stress, and other studies have looked at how adaptation to 1 stressor protects the bacteria against other stressors [7,74]. Another example of bacteria adapting to changing environments is how *P*. *aeruginosa*, which can be found in the natural environment in the soil and water, can readily adapt to a human host under the right conditions and consequently become pathogenic [75].

There are several factors involved in the emergence of antibiotic resistance that are clinically important that are not considered in this study. We have not taken into account any pathogen/host interactions, such as the role of the immune system. We also do not take into consideration the pharmacokinetics of the drug and the time-dependent fluctuation of drug concentration as experienced by the bacteria in a human-host environment. Furthermore, the dosages of clinical regimens are typically much higher than the wild-type MIC, and the evolutionary dynamics of the bacteria under these conditions may be different from those seen in our study, in which the drug pressure is slowly increased over time. We neglect to consider the role of horizontal gene transfer, which is a common mechanism of antibiotic resistance transfer, and focus rather on the role of de novo mutations acquired during adaptation. Because of the nature of the serial passaging method, we may be selecting for fast growers that may not necessarily have mutations that confer the most amount of resistance in terms of the MIC. We used a strong selection pressure in this study by propagating from the highest concentration of drug that showed growth, but it has been shown that weak antibiotic selection pressure can greatly affect the adaptive landscape [76,77]. Lastly, these bacteria were evolved to 1 antibiotic at a time, and we do not know how different mutant lineages would adapt if competed against each other. It would be interesting in the future to conduct competition experiments to measure the fitness of the different lineages with respect to each other.

While adaptive evolution of clinical isolates suggests that the drug-order–specific effects are clinically relevant, actual clinical studies must be performed to test the true clinical applicability of these effects. A major challenge that still needs to be addressed is how to translate the results of in vitro adaptive evolution experiments to effective therapies that can be used in a clinical setting [78]. For example, while we observed that piperacillin adaptation often led to the large chromosomal deletions and concomitant pyomelanin hyperproduction, the clinical isolates that had the large deletions (Fig 8) were not necessarily resistant to piperacillin. On the other hand, the other set of clinical isolates, which did have resistance to piperacillin, did not have the large deletions (Fig 7). Disparities between the phenotypic and genotypic adaptations such as this will need to be studied further in terms of strain-specific differences, actual history of antibiotic exposure, and other factors that are beyond the scope of this study.

Despite these caveats, there are several key factors of this study that provide confidence in the claims made. We saw consistency in the parallel replicates for the treatment lineages. An interesting exception is Day 40 PIP^R^TOB^R^-4, which had a higher final tobramycin resistance compared to Day 40 PIP^R^TOB^R^-1, -2 and -3, which we believe is attributed to the large genomic deletions seen in the first 3 replicates but not in the fourth replicate. We observed parallel evolution in which several genes were mutated independently across multiple lineages, and overall, there were less than 15 mutations per 20 days of evolution, which suggests positive selection. Furthermore, many of the mutated genes are also observed in clinical isolates of *P*. *aeruginosa*, further giving credence to the clinical relevance of these mutations.

As mentioned previously, studies that have looked at alternating treatments of antibiotics have primarily looked at the effects of rapid switching, typically at daily or subdaily intervals. One of such recent studies evaluated how *E*. *coli* responded to 136 different sequential treatments of subinhibitory concentrations of doxycycline and erythromycin, with each treatment consisting of 8 “seasons” of 12-hour-long adaptation periods to 1 of the drugs [19]. Using final optical density as an endpoint metric, the study found that 5 of the sequential treatments could clear the bacteria at the end of the eighth season. Interestingly, one of those 5 successful treatments consisted of 4 seasons of erythromycin, followed subsequently by 4 seasons of doxycycline. On the other hand, the treatment consisting of 4 seasons of doxycycline followed by 4 seasons of erythromycin did not manage to clear the bacteria at the end of 8 seasons. While the experimental setup is much different compared to that of this present study in terms of organism, antibiotics used, duration of treatment, and endpoint metric, these 2 treatments (4 seasons of erythromycin then 4 seasons of doxycycline and vice versa) are quite analogous to the types of treatments tested in our present study. The fact that these authors found a difference in the outcomes of this pair of opposite sequential treatments may suggest that drug-order–specific effects similar to those presented in our study may play a role in the evolutionary dynamics of their experiments.

Cycling between 2 drugs that exhibit collateral sensitivity to one another has been proposed and tested as a strategy to slow down the rate of resistance development [14]. Studies that have systematically tested for collateral sensitivities across a variety of antibiotics in *E*. *coli* have consistently discovered that when *E*. *coli* is adapted to drugs of the aminoglycoside class, it develops collateral sensitivity to several other drugs of different classes including beta-lactams, DNA synthesis inhibitors, and protein synthesis inhibitors [14,16,77]. In our present study, we tested 1 aminoglycoside (tobramycin), and we did not observe any collateral sensitivity arise to piperacillin or ciprofloxacin during adaptation to tobramycin. Instead, we saw collateral sensitivity to piperacillin and tobramycin arise as *P*. *aeruginosa* was adapted to ciprofloxacin, which is a DNA synthesis inhibitor. While we only tested 1 drug in each of 3 drug classes, the dissimilarity of collateral sensitivity profiles between those studies and this present study may highlight how collateral sensitivity profiles may be organism-specific and drug-specific. Further supporting this idea, these prior studies also showed that while adaptation to drugs of the aminoglycoside class as a whole tended to lead to collateral sensitivity to other drug classes, not every aminoglycoside drug that was tested induced the same collateral sensitivity profiles.

While we did observe cases of collateral sensitivity, the main focus of our study was not to look at how resistance profiles to other drugs **concurrently** change during adaptation to 1 drug, but rather to see how adaptation to 1 drug influences the **future** evolutionary dynamics as the resistant population adapts to a new drug. Additionally, we wanted to see how adaptation to the second drug affected the resistance profile of the drug that the bacteria originally developed resistance to. Our sustained drug adaptation scheme can be thought of as being more akin to month-long antibiotic cycling at the level of the hospital ward or the environments that bacteria in persistent chronic infections are exposed to. The history-dependent evolutionary dynamics presented in this study highlight the complexity of bacterial adaptation to multidrug therapies, serve as a framework for forecasting evolutionary trajectories based on genetic and phenotypic signatures of past adaptation, and ultimately help elucidate our fundamental understandings of the evolutionary forces that drive resistance adaptation.

Asymmetrical evolutionary responses in changing environments have been studied in terms of collateral sensitivity/resistance [14,16], temperature [79], other abiotic stresses [7], and in cancer treatments [80]. Here, we present the concept of drug-history–specific effects in multidrug resistance adaptation, whereby the history of adaptation to 1 antibiotic environment can influence the evolutionary dynamics during subsequent adaptation to another antibiotic environment. These history-specific effects have direct clinical implications on optimizing antibiotic treatment strategies to slow and prevent the emergence of dangerous multidrug-resistant bacterial pathogens.

## Materials and methods

### Ethics statement

The set of *P*. *aeruginosa* clinical isolates collected from the University of Virginia Health System (presented in Fig 7) were deidentified, did not require Institutional Review Board approval for their use, and were anonymized. The Hocquet *P*. *aeruginosa* clinical isolates (which were originally collected by the authors of the Hocquet study [64]; presented in Fig 8) also did not require Institutional Review Board approval for their use and were anonymized.

### Experimental study design

We evolved, in parallel, 4 independent replicates for each lineage in the primary adaptive evolution experiment and 3 independent replicates for each of the clinical isolates to balance the statistical power of the conclusions with the technical feasibilities of the daily serial propagations. In the primary adaptive evolution experiment, we concluded the 1-drug evolution at the end of 20 days because the resistance levels of the evolved lineages to their respective drugs were saturated or close to saturated at that point. The clinical isolates from Fig 7 and from Fig 8 were evolved for 10 and 15 days, respectively, because the similarities and differences of the drug-specific effects to those of the primary adaptive evolution experiment were readily apparent at that point.

### Media, growth conditions, and antibiotics

MIC plates were made daily using the broth microdilution method with the standard 2-fold dilution series [81]. LB was used as the growth medium for all experiments (1% tryptone, 0.5% yeast extract, 1% NaCl). Antibiotics tested include piperacillin sodium, tobramycin, and ciprofloxacin HCl (Sigma). Aliquots of 1 mg/ml and 10 mg/ml antibiotic stocks were made by diluting the antibiotic powders in LB and were stored at −20°C. New frozen drug aliquots were used on a daily basis.

### Adaptive laboratory evolution

A frozen stock of *P*. *aeruginosa* PA14 was streaked on an LB agar plate, and a single colony was inoculated into 4 ml of LB, which was then grown overnight at 37°C, shaking at 125 RPM. This antibiotic-susceptible culture, denoted as the Day 0 Ancestor, was diluted to an OD_600_ of 0.001 (approximately 10^6^ CFU/ml) and then inoculated into 3 identical MIC plates consisting of concentration gradients of piperacillin and tobramycin. A sample of the ancestor was saved in 25% glycerol and stored at −80°C. The 3 MIC plates were used to serially propagate cultures evolved to LB media, piperacillin, and tobramycin, with 4 biological replicates per condition. Wells for growth control (media + culture) and sterility control (media) were included in each MIC plate. For adaptation to LB media, bacteria were sampled from the growth control well. MIC plates were placed in a plastic container (to prevent evaporation) and incubated at 37°C with shaking at 125 RPM (Thermo Scientific MaxQ 4000). MIC plates were incubated daily for approximately 23 hours.

At the end of incubation, growth in the MIC plates was determined using a plate reader (Tecan Infinite M200 Pro). Growth was defined as OD_600_ > 0.1 after background subtraction. We recorded the MIC of each lineage for each drug, which was defined as the lowest antibiotic concentration tested that did not show growth (S1 and S3 Data). To propagate, cultures were passaged from the highest concentration that showed growth (i.e., MIC/2) from the corresponding MIC drug gradient. For adaptation to LB, cultures were passaged from the growth control well that contained only LB without any drug. For each culture to be passaged, the culture was first diluted by a factor of 1/250 in fresh LB (e.g., 20 μl of the culture was diluted in 5 ml of LB), which was then inoculated in fresh piperacillin and tobramycin drug gradients in the new day’s MIC plate. Wells of the MIC plate thus contained 100 μl of double the final concentration of the antibiotic and 100 μl of the diluted culture. Hence, the cultures were diluted by a total factor of 1/500 daily. Daily samples were saved in 25% glycerol and stored at −80°C. For Day 21, the piperacillin and tobramycin evolved cultures were subcultured in additional MIC plates such that they could subsequently be evolved to tobramycin and piperacillin, respectively.

A similar protocol was used to establish the ciprofloxacin-evolved lineages (CIP^R^). Starting with a clonal population of the Day 0 Ancestor, 4 replicates were established and propagated daily under ciprofloxacin treatment for 20 days. CIP^R^ was then subpassaged to piperacillin and tobramycin to establish the CIP^R^PIP^R^ and CIP^R^TOB^R^ lineages in addition to continued ciprofloxacin evolution.

To establish the PIP^R^CIP^R^ and TOB^R^CIP^R^ lineages, bacteria from the frozen stocks of Day 20 PIP^R^ and TOB^R^ were revived on LB agar plates, and clonal populations were evolved to ciprofloxacin to establish these lineages. Similarly, to establish the PIP^R^LB, TOB^R^LB, and CIP^R^LB lineages, bacteria from the frozen stocks of Day 20 PIP^R^, TOB^R^, and CIP^R^ were revived on LB agar plates, and clonal populations were evolved to LB.

Lastly, the MIC to ciprofloxacin was retrospectively measured for the Control, PIP^R^, TOB^R^, PIP^R^TOB^R^, and TOB^R^PIP^R^ lineages. Frozen stocks were revived and plated on LB agar plates. The notation for the day numbering is such that Day X PIP^R^ means X days exposure to piperacillin. For consistency, stocks were revived from Days 0 (Ancestor), 5, 10, 15, 19, 20, 25, 30, 35, and 39 for Control, PIP^R^, and TOB^R^. One day of exposure to ciprofloxacin would yield Days 1, 6, 11, 16, 20, 21, 26, 31, 36, and 40 MICs to ciprofloxacin. For PIP^R^TOB^R^ and TOB^R^PIP^R^, stocks were similarly revived from Days 20, 25, 30, 35, and 39 and exposed to ciprofloxacin to measure Days 21, 26, 31, 36, and 40 MICs to ciprofloxacin. S3 Data shows the MICs to piperacillin, tobramycin, and ciprofloxacin, respectively, for all the lineages. Note that not all drug MICs were measured on a daily basis for all lineages.

During analysis of the mutations, we deduced that there were some cross-contaminations between replicates in a few lineages. Namely, we saw sets of mutations that were identical in 2 replicates. We believed that the most likely explanation was that the following 7 lines were cross-contaminated sometime between Day 21 and Day 40: CIP^R^PIP^R^-3, CIP^R^PIP^R^-4, TOB^R^-1 CIP^R^TOB^R^-1, CIP^R^TOB^R^-2, CIP^R^TOB^R^-4, and CIP^R^-3, where the number denotes the replicate. To redo these lineages, the corresponding Day 20 replicate frozen stocks were revived on LB agar plates. Then clonal populations were used to redo the propagation as described before. For example, CIP^R^-3 was evolved to piperacillin for 20 days to redo CIP^R^PIP^R^-3. We performed Sanger sequencing of replicate-specific mutations (S3 Table) on the Day 40 mutants to confirm successful propagation of the cultures.

### Whole-genome sequencing

Frozen samples of Day 0 Ancestor, Day 20 Control, PIP^R^, TOB^R^, CIP^R^, Day 40 Control, PIP^R^, TOB^R^, CIP^R^, PIP^R^TOB^R^, PIP^R^CIP^R^, TOB^R^PIP^R^, TOB^R^CIP^R^, CIP^R^PIP^R^, and CIP^R^TOB^R^ were streaked on LB agar plates and incubated at 37°C. Agar plates were submitted to Genewiz Incorporation for sequencing service. A single colony from each plate was chosen for DNA extraction, library preparation, multiplexing, and sequencing using 101-bp paired-end reads with the Illumina HiSeq 2500 platform. Reads were aligned to the reference *P*. *aeruginosa* PA14 genome (NC_008463.1) with coverage ranging from 113X to 759X. This large range is due to the fact that we submitted samples for sequencing in 3 batches and had different numbers of samples for each batch but had relatively the same number of reads per batch. Nevertheless, the coverage was more than sufficient to identify the SNPs, insertions, and deletions in the genomes. The sequencing reads for Day 0 Ancestor and the 56 drug-evolved lineages are available via the NCBI SRA database (www.ncbi.nlm.nih.gov/sra), accession number SRP100674, BioProject number PRJNA376615.

Reads were aligned and mutations were called using the breseq pipeline [82] using default settings. All reported mutations were visually inspected by viewing the read alignments in IGV and the breseq output files, and mutations with less than 80% frequencies were not counted. The full list of mutations is presented in S1 and S2 Tables. The circos software package [83] was used to plot the mutations by genomic position for Fig 4 and the positions of the large chromosomal deletions in Fig 8.

We confirmed some of the mutations using Sanger sequencing. For each of the Day 20 PIP^R^, TOB^R^, and CIP^R^ replicates, we chose 1 mutation each to confirm (S3 Table). We also used these to confirm that replicates were not contaminated before submitting them for whole-genome sequencing. These mutations were also confirmed in each of the Day 40 lineages that were derived from the Day 20 PIP^R^, TOB^R^, and CIP^R^ replicates.

### Reproducing drug-history dependence in the pyomelanin phenotype during piperacillin evolution

Clonal populations of Day 0 Ancestor, Day 20 TOB^R^-1, -2, -3 and -4, and Day 20 CIP^R^-1, -2, -3, and -4 were grown in LB starting from the frozen samples. These cultures were diluted in LB to OD_600_ of 0.001. On Day 1, in 96-well plates, 100 μl of the diluted cultures were inoculated with 100 μl of 4 μg/ml piperacillin (to yield a final concentration of 2 μg/ml piperacillin). Ninety-two wells were used to establish independent replicate populations exposed to piperacillin. Cultures were incubated at 37°C with shaking at 125 RPM. On Day 2, replicate populations were passaged using a 96-pin replicator tool (V&P Scientific, VP246, 100–150 μl per pin) into 200 μl of 4 μg/ml piperacillin. This protocol was continued until Day 10 with a final concentration of 20 μg/ml piperacillin. For each plate, 2 wells were used as sterility controls (only LB), and 2 wells were used as growth controls (LB with bacteria, without drug). Photographs were taken daily (S8–S10 Figs), and the number of visibly brown wells was recorded.

### Testing for drug-order–specific evolutionary dynamics in clinical isolates

Three clinical isolates of *P*. *aeruginosa* with high piperacillin resistance and low tobramycin and ciprofloxacin resistance were obtained from the University of Virginia Health System and were evolved to the 3 drugs in the same manner as the main adaptive evolution experiment starting from frozen samples. These isolates were first confirmed to actually be *P*. *aeruginosa* with PCR by using primers that specifically amplify the 16S rRNA region of *P*. *aeruginosa* [84]. Three replicates of each isolate were evolved to each of the 3 drugs for 10 days, and their MICs to the 3 drugs were measured as before. In separate subsequent experiments, the 3 clinical isolates were evolved to LB with 3 replicates each. The MIC_PIP_ was measured for 10 days (S3 Data). This measurement was done by inoculating into piperacillin concentration gradients to measure the MIC_PIP_ but sampling and passaging from the “growth control” well (LB with bacteria, without drug) to adapt to LB.

The 4 pairs of clinical isolates of *P*. *aeruginosa* from the Hocquet study [64] were evolved to tobramycin for 15 days with 3 parallel replicates each, with the exception of B_PM_, which had 2 replicates due to cross-contamination in the third replicate. The MICs for piperacillin and ciprofloxacin were also measured every 5 days (S3 Data). At the end of the 15 days of evolution, primers amplifying part of the *hmgA* gene were used to check for the presence of the gene in the “WT” isolates and the absence of the gene in the “PM” isolates (S3 Table).

### Statistical significance of drug-order–specific effects in MIC profiles

All statistical comparisons of MIC values were performed on the log_2_ transformed values. Unless noted otherwise, one-way ANOVAs were performed on the MICs of the relevant lineages. If the *p*-value from the ANOVA was less than 0.05, a post-hoc Tukey’s honest significant difference (HSD) multiple comparisons test was then performed to determine which pairs of treatments were significantly different from each other. The Tukey’s HSD tests report 95% confidence intervals for the true mean difference for each pairwise comparison. If the confidence interval does not contain 0, then the 2 groups being compared have significantly different means at the *p* = 0.05 level. To also assess the comparisons using nonparametric statistic tests, Kruskwal-Wallis tests followed by post-hoc Dunn’s multiple comparisons tests were also performed. All of the Kruskal-Wallis tests yielded comparable results to the one-way ANOVA at the alpha = 0.05 significance level, and the conclusions are the same for the key comparisons that drive the results highlighted in the manuscript. For a complete set of calculations, see S2 Text.

For the comparisons presented in Fig 3, treatments being compared consist of those listed on the *x*-axis of each graph in the figure. For the comparisons presented in Fig 7, the raw MIC values for each lineage were first normalized by subtracting the average Day 1 MIC of each of their respective lineages. For each of the 3 clinical isolates, a one-way ANOVA and a Kruskal-Wallis test were performed on the Day 10 MIC_PIP_ values of the lineages evolved to LB, tobramycin, and ciprofloxacin (piperacillin-adapted lineages were excluded in the comparisons). The Tukey’s HSD test and Dunn’s test were then performed to see if the Day 10 MIC_PIP_ values of the lineages evolved to tobramycin and ciprofloxacin were significantly different from the lineages evolved to LB. For the comparisons presented in Fig 8, the raw MIC values for each lineage were first normalized by subtracting the average Day 1 MIC of each of their respective lineages. A two-sample *t* test and a Wilcoxon rank sum test were performed for the Day 15 MIC_TOB_ values of the “WT” and “PM” lineages evolved to tobramycin in each of the 4 pairs of isolates. Calculations were done in MATLAB R2016b, using the functions “anova1” for one-way ANOVA, “multcompare” for Tukey’s HSD test, “ttest2” for two-sample *t* test, and “ranksum” for the Wilcoxon rank sum test. The Kruskal-Wallis test was done with the “kruskal.test” command in R, and the Dunn’s test was done with the “posthoc.kruskal.dunn.test” command with the PMCMR R package [85].

## Supporting information

S1 Text Supplementary text. This file contains supplementary text on the analysis of growth rates, confirmation of mutations in evolved lineages, and extended analysis of mutations.
(PDF)

S2 Text Statistical tests. This file contains the calculations for all statistical tests performed in this study.
(PDF)

S1 Fig Distribution of retrospective MIC measurements. Minimum inhibitory concentration (MIC) measurements were performed using 4 colonies per lineage replicate to test the MICs of the frozen permanent stocks. S2 Data shows which MICs were measured retrospectively for the different lineage replicates and the values of the original and retrospectively measured MICs. The histogram shows the distribution of the difference between the retrospective and original MICs for the 320 retrospectively measured MICs. Overall, 234 of the 320 (73%) retrospectively measured MICs were within one 2-fold dilution step of the originally measured MIC on either direction (−1, 0, and 1 on the *x*-axis).
(TIF)

S2 Fig Summary of the minimum inhibitory concentration (MIC) time courses. This figure summarizes the data presented in Fig 2 of the main text. The Day 1, Day 20, and Day 40 log_2_ MIC values (μg/ml) of piperacillin (PIP), tobramycin (TOB), and ciprofloxacin (CIP) are shown for all the evolved lineages of the main adaptive evolution experiment. The values are the average of 4 replicates per lineage (S3 Data). For each lineage, the left, middle, and right boxes denote the MIC_PIP_, MIC_TOB_, and MIC_CIP_, respectively. The color intensity is normalized by the minimum and maximum MIC of each drug across all the lineages. For example, for log_2_ MIC_PIP_, the lowest value is 1.5, which is seen in Day 40 CIP^R^, and the highest log_2_ MIC_PIP_ is 9.5, which is seen in Day 40 PIP^R^. The color of the arrow denotes the treatment.
(TIF)

S3 Fig Visualization of drug-order–specific effects and quantification of the changes in minimum inhibitory concentration (MICs). All values shown are the averages of 4 replicates (S3 Data). (A) The MICs of the 3 drugs for Days 1, 20, and 40 for all treatments are plotted in 3D MIC space to show how the MIC profiles change over the course of adaptation. Day 1 MICs are denoted by the triangles. A “nonright angle” indicates a change in resistance to 1 (or more) of the other drug(s). The color/style of the line indicates the treatment and is labeled as such. (B to D) 2D projections of (A). Labels for the lines carry over from (A). (E) Changes in average MICs for all drugs for all treatments are plotted on a single axis to better facilitate quantitative comparison. Here, red, blue, and yellow lines denote MICs to piperacillin (PIP), tobramycin (TOB), and ciprofloxacin (CIP), respectively.
(TIF)

S4 Fig Collateral sensitivity of piperacillin (PIP) and tobramycin (TOB) during ciprofloxacin (CIP) adaptation. (A) Collateral sensitivities to tobramycin (left) and piperacillin (right) were observed during the evolution starting from Day 0 Ancestor to ciprofloxacin. While there were no statistically significant changes in MIC_TOB_ and MIC_PIP_ after 20 days of evolution to LB in the Control, there were significant decreases after 20 days of evolution to ciprofloxacin. Similarly, (B) there was a significant decrease in MIC_TOB_ when Day 20 PIP^R^ was subsequently adapted to ciprofloxacin, (C) and in MIC_PIP_ when Day 20 TOB^R^ was subsequently adapted to ciprofloxacin. For all 3 panels, the asterisks denote *p* < 0.05 (Tukey’s HSD test), n.s. denotes *p* > 0.05, and the color of the asterisks denotes which drug minimum inhibitory concentration (MIC) is being compared. In the plots, for each lineage being shown, the black bar denotes the mean of the 4 individual replicate values (gray dots). See S2 Text for the calculations of the statistical tests and S3 Data for the raw MIC data.
(TIF)

S5 Fig Growth curves of evolved lineages. The OD_600_ was measured over the course of 24 hours for the 68 evolved replicate lineages (17 lineages of 4 biological replicates each) as well as the Day 0 Ancestor in quadruplicates grown in lysogeny broth (LB). Note that because of the pyomelanin hyperproduction, replicates 1, 2, and 3 of the PIP^R^ and PIP^R^-derived lineages reach higher final OD_600_’s than the replicate 4 lineages as is apparent in the curves above (as discussed in the main text).
(TIF)

S6 Fig Analysis of growth rates of the evolved lineages. (A) The growth rates were calculated from the growth curves presented in S5 Fig. The means and standard deviations of the 4 replicates for each of the replicate lineages are shown. (B) The correlation between the growth rates of the Day 40 lineages (*x*-axis) and the change in minimum inhibitory concentration (MIC) of the corresponding Day 20 lineages (*y*-axis) was calculated. The data suggest no correlation between growth rate and the degree to which the MIC changes from Day 20 to Day 40. For example, the 4 red crosses show the growth rates of Day 40 PIP^R^TOB^R^-1, -2, -3, and -4 on the *x*-axis plotted against the log_2_ MIC_PIP_ of Day 20 PIP^R^-1, -2, -3, and -4 minus the log_2_ MIC_PIP_ of Day 40 PIP^R^TOB^R^-1, -2, -3, and -4, respectively on the *y*-axis. See S4 Data for the growth rate data.
(TIF)

S7 Fig Distribution of mutations. Histogram of the number of mutations shows that overall, lineages that were evolved to ciprofloxacin accumulated the most mutations and had comparably more deletion mutations. See S1 Table for the complete list of mutations.
(TIF)

S8 Fig Reproducibility of pyomelanin phenotype during piperacillin evolution starting from Day 0 Ancestor. Ninety-two replicates of Day 0 Ancestor were serially passaged with a replicator tool for 10 days to increasing concentrations of piperacillin.
(TIF)

S9 Fig Reproducibility of pyomelanin phenotype during piperacillin (PIP) evolution starting from Day 20 TOB^R^. Ninety-two replicates of Day 20 TOB^R^-1, -2, -3, and -4 were serially passaged with a replicator tool for 10 days to increasing concentrations of piperacillin.
(TIF)

S10 Fig Reproducibility of pyomelanin phenotype during piperacillin (PIP) evolution starting from Day 20 CIP^R^. Ninety-two replicates of Day 20 CIP^R^-1, -2, -3, and -4 were serially passaged with a replicator tool for 10 days to increasing concentrations of piperacillin.
(TIF)

S11 Fig Evolutionary dynamics in clinical isolates with high piperacillin (PIP) resistance. Three clinical isolates of *P*. *aeruginosa* with high piperacillin resistance were evolved to piperacillin, tobramycin, and ciprofloxacin to test if we could recapitulate the evolutionary dynamics seen in MIC_PIP_ of PIP^R^, whereby evolution to ciprofloxacin would cause MIC_PIP_ to decrease, while evolution to tobramycin would not. We were able to see this result recapitulated in isolate #2 and isolate #3, but not in isolate #1 (see main text). Interestingly, isolate #1 was able to be evolved to higher levels of piperacillin resistance and ciprofloxacin resistance compared to the other 2. Thin lines show the individual time courses of 3 replicates per treatment, and bold lines show their averages. The dotted line in the first row shows the mean MIC_PIP_ of Day 1 Control to emphasize that the clinical isolates are resistant to piperacillin at Day 1. Error bars show SEM for the 3 replicates for each lineage. See S3 Data for the raw numerical data.
(TIF)

S12 Fig Drug history-dependence in MIC_TOB_ and large deletions in PIP^R^. The resistance levels to tobramycin for individual replicates are plotted for Day 20 PIP^R^ and Day 40 PIP^R^TOB^R^. The replicates denoted with the filled-in circles have large deletions in their genome, while the replicate denoted by the open circle does not. We see that the replicates of Day 20 PIP^R^ with the large chromosomal deletions develop less resistance to tobramycin than the replicate that does not have the deletion. See S3 Data for the raw numerical data.
(TIF)

S13 Fig Evolutionary dynamics in clinical isolates with large chromosomal deletions. Four pairs of clinical isolates of *P*. *aeruginosa* were evolved to tobramycin. Each pair of isolates (columns) consists of a pyomelanogenic isolate (PM) that has a large deletion, and a parental isolate from which the PM isolate is derived (wild-type [WT]). In each pair, the only genetic difference is the presence of a large chromosomal deletion in the PM isolate. The top, middle, and bottom rows show the minimum inhibitory concentrations (MICs) of the isolates to tobramycin, piperacillin, and ciprofloxacin, respectively, as they adapt to tobramycin. Thin lines show the individual time courses of 3 replicates per treatment (with the exception of B_PM_, which has 2 replicates), and bold lines show their averages. Error bars show SEM for the 3 replicates (2 for B_PM_) for each lineage. See S3 Data for the raw numerical data.
(TIF)

S1 Data Number of generations of growth in evolved lineages. This file shows the OD_600_ measurements of the wells from which bacteria were passaged for each of the evolution experiments and the calculations for the estimated number of doublings.
(XLSX)

S2 Data Retrospective minimum inhibitory concentration (MIC) measurements. This file contains the retrospectively measured MICs of a subset of the replicate lineages from the main adaptive evolution experiment, and the differences between the retrospective and the original MICs.
(XLSX)

S3 Data Raw data of MIC_PIP_, MIC_TOB_, and MIC_CIP_. This file contains the data used in Figs 2, 3, 7 and 8. Note that not all lineages were tested for resistance to each drug at a daily resolution. Also, note that the ciprofloxacin resistance was measured retrospectively for the Control, PIP^R^, TOB^R^, PIP^R^TOB^R^, and TOB^R^PIP^R^ lineages.
(XLSX)

S4 Data Growth curves and growth rates analysis. This file contains the growth rates of the evolved lineages and the data for the subsequent analysis of the growth rates.
(XLSX)

S5 Data Genes in large deletions. This file lists the genes and their relevant information of the large chromosomal deletions of PIP^R^-1, PIP^R^-2, PIP^R^-3, A_PM_, B_PM_, C_PM_, and D_PM_.
(XLSX)

S1 Table Complete list of mutations. 1’s and 0’s denote the presence and absence of mutations, respectively. The 2 mutations highlighted in green denote synonymous SNPs.
(DOCX)

S2 Table Descriptions of mutated genes. (DOCX)

S3 Table Mutations confirmed by Sanger sequencing. (DOCX)

S4 Table Frequently mutated genes. Values denote the number of different lineages that had mutations in the specified gene for the given treatment. Values are not double counted if passed on from Day 20 to Day 40, e.g., a mutation that occurred in Day 20 PIP^R^ that carried over to Day 40 PIP^R^, PIP^R^TOB^R^, and PIP^R^CIP^R^ is counted as 1 lineage.
(DOCX)

## Abbreviations

CIP: ciprofloxacin
HSD: honest significant difference
LB: lysogeny broth
MIC: Minimum inhibitory concentration
PIP: Piperacillin
PM: pyomelanogenic
TOB: tobramycin
WT: wild-type
