# Supplementary material for: History of antibiotic adaptation influences microbial evolutionary dynamics during subsequent treatment
Source: PLoS Biol. 2017 Aug 8;15(8):e2001586. doi: 10.1371/journal.pbio.2001586 (PMC5549691; doi:10.1371/journal.pbio.2001586)
Supplement: S1 Text — This file contains supplementary text on the analysis of growth rates, confirmation of mutations in evolved lineages, and extended analysis of mutations. (PDF) [file pbio.2001586.s001.pdf]

Supplementary Text (S1 Text) for

History of Antibiotic Adaptation Influences Microbial Evolutionary Dynamics During Subsequent Treatment

Phillip Yen and Jason Papin*

*Corresponding author. E-mail: papin@virginia.edu

Analysis of growth rates. We measured the growth curves (OD_{600}) of the 68 evolved replicate lineages (17 lineages of 4 biological replicates each) as well as the Day 0 Ancestor in quadruplicates grown in LB broth for 24 hours (S5 Fig). Subsequently, the growth rates were calculated from the growth curves using the Curve Fitter analysis software (N. F. Delaney, CJM, unpublished; http://www.evolvedmicrobe.com/Software.html), which was used in [1]. S6A Fig shows the growth rates for the 68 evolved lineages and the Day 0 Ancestor. While we observed many different growth rates across the different lineages, we did not find evidence to support the hypothesis that slower growers led to the drug resensitization during subsequent evolution of the Day 20 one-drug-evolved lineages to subsequent treatments. Specifically, S6B Fig shows that there is no correlation between the growth rate of the Day 40 lineages and the changes in MIC from the corresponding Day 20 lineages from which they were derived from. For example, while both Day 40 TOB^{R}LB and TOB^{R}CIPR became partially resensitized to tobramycin from Day 20 TOBR (Fig 3A, middle), this occurred independently of growth rate, as Day 40 TOB^{R}LB (blue diamonds in S6B Fig) had a higher growth rate than Day 40 $TOB^{R}CIP^{R}$ (blue circles in S6B Fig). Furthermore, Day 40 $TOB^{R}PIP^{R}$ (blue crosses in S6B Fig) maintained relatively high tobramycin resistance, yet the growth rates of those four replicates spanned within the ranges of those of Day 40 $TOB^{R}CIP^{R}$ and TOB^{R}LB. We would also like to emphasize that the daily propagation protocol resulted in approximately nine generations of growth in all of the evolved lineages (S1 Data). Thus, independently of growth rate, we consistently propagated bacteria from the MIC concentration gradients from populations that have undergone ~9 generations of growth in ~24 hours.

Confirmation of mutations in evolved lineages. To test how representative the sequencing results were of the mutant populations, we used PCR and Sanger sequencing to test for the presence of specific mutations in multiple colonies of different lineages. We used the primers from S3 Table to test for the presence of one mutation from one replicate of each lineage, with four colonies of each lineage. For example, for the PIP^{R} lineages, we used the P1_dacC primers to check for the presence of the *dacC* C→T mutation in four colonies each of Day 20 PIP^{R}-1, Day 40 PIP^{R}-1, Day 40 $PIP^{R}TOB^{R}$-1, Day 40 $PIP^{R}CIP^{R}$-1, and Day 40 PIP^{R}LB-1 using colony PCR followed by Sanger sequencing. The *dacC* mutation was observed in all four colonies of each of these tested lineages. Similarly, for the CIP^{R} lineages, we used the F1_aotJ primers to check for the presence of the *aotJ* Δ1 bp mutation in four colonies each of Day 20 CIP^{R}-1, Day 40 CIP^{R}-1, Day 40 $CIP^{R}PIP^{R}$-1, Day 40 $CIP^{R}TOB^{R}$-1, and Day 40 CIP^{R}LB-1. The *aotJ* mutation was observed in all four colonies of each of these tested lineages. Lastly, for the TOB^{R} lineages, we used the T1_25490 primers to check for the presence of the PA14_25490 T→C mutation in four colonies each of Day 20 TOB^{R}-1, Day 40 TOB^{R}-1, Day 40 $TOB^{R}PIP^{R}$-1, Day 40 $TOB^{R}CIP^{R}$-1, and Day 40 TOB^{R}LB-1. The PA14_25490 mutation was observed in all four

colonies of each of these tested lineages, except for Day 20 TOB^R-1, where 1 of the 4 colonies had the mutation and 3 of the 4 colonies had the wild-type base pair. To investigate the potential heterogeneity of Day 20 TOB^R-1, we checked for the presence of a different mutation (the *fusA1* T→C SNP) using the T1_fusA1 primers in addition to the PA14_25490 T→C SNP in 8 colonies of Day 20 TOB^R-1. Here, we found that all 8 eight colonies had the *fusA1* SNP and 2 of the 8 colonies had the PA14_25490 SNP. These experiments suggest that while there may be limited heterogeneity in the populations with respect to a few of the mutations, the large majority of the mutations are homogeneous in the populations and fixed within the lineages.

Extended analysis of mutations. Several of the *P. aeruginosa* multidrug efflux pumps (MexAB-OprM, MexCD-OprJ, MexEF-OprN, MexXY-OprM [2]) and their associated transcription factors were also common targets for mutations during evolution to all three drugs. The *nfxB* gene was the second most mutated gene. There were seven deletion mutations (all resulting in frameshifts) in seven separate ciprofloxacin-evolved lineages and one insertion mutation in the intergenic region between *nfxB* and *mexC* in a tobramycin-evolved sample. *nfxB* codes for a negative transcriptional regulator of the MexCD-OprJ efflux pump, and *nfxB*-type mutants overexpress the normally repressed MexCD-OprJ system [2]. *nfxB* has been observed to be mutated frequently during adaptive evolution to ciprofloxacin [3]. Inactivation of *nfxB* results in de-repression of the transcription of the MexCD-OprJ efflux pump, which contributes resistance to fluoroquinolones, macrolides, tetracycline, and some beta-lactams [2]. The one intergenic insertion occurred in the binding site of *nfxB* [4] of Day 40 CIP^{R}TOB^R-4 during the tobramycin evolution. This sample also acquired a 16 base pair deletion during the earlier ciprofloxacin evolution (Day 20 CIP^R-4). Thus, it seems that this sample interestingly has a non-functional NfxB protein and most likely non-functional NfxB binding site as well. Also interestingly, overexpression of the MexCD-OprJ pump has been reported to result in hypersusceptibility to beta-lactams and aminoglycosides [5,6].

Other mutations involving the multidrug efflux pumps and their regulators include: *nalD* (PA14_18080), *mexS* (PA14_32420), *mexC* (PA14_60850), *mexR* (PA14_05520), *nalC* (PA14_16280), *parS* (PA14_41270), *amrB* (aka *mexY*, PA14_38410), *mexA* (PA14_05530), *mexD* (PA14_60830), *mexF* (PA14_32390), *mexT* (PA14_32410), and *muxA* (PA14_31870). We even saw a SNP located exactly at the predicted transcription site of PA3720-*armR* in *P. aeruginosa* PAO1 [7] (corresponding to PA14_16290-PA14_16300 in PA14), which is a possible region of a NalC binding site. NalC is a repressor of MexAB-OprM expression.

There were a few genes that were repeatedly mutated that are not very well characterized in the literature. Three frameshift mutations occurred in PA14_09960 in three different ciprofloxacin treatments (Day 40 CIP^R-2, Day 40 PIP^{R}CIP^R-4, and Day 40 TOB^{R}CIP^R-3). This hypothetical protein has a Pfam description of being an Rrf2-like transcription regulator. Two frameshift mutations occurred in PA14_35210 in two different ciprofloxacin treatments (Day 40 CIP^R-1, and Day 40 CIP^R-2), and it is annotated as being a TetR family transcriptional regulator. Lastly, two SNPs occurred in PA14_51910 in two different piperacillin treatments (Day 20 PIP^R-3 and all progeny lineages, and Day 40 TOB^{R}PIP^R-2), suggesting that the hypothetical protein plays a role in piperacillin resistance.

Large deletions of the genome (>100 kbp) were observed in multiple lineages. Three of the lineages evolved to piperacillin (Day 20 PIP^R-1, -2, and -3) sustained ~400 kbp deletions (encoding ~350 genes), which subsequently fixed in their respective Day 40 PIP^R, PIP^{R}TOB^R, and PIP^{R}CIP^R lineages. These three deletions all occurred within a conserved region of the

chromosome, and they overlap each other by ~190 kbp (encoding ~160 genes) (S5 Data). We also observed a ~176 kbp deletion occur in this same region during adaptation to ciprofloxacin after prior adaptation to tobramycin ($TOB^R CIP^R$-2), which suggests that this deletion is not specific to piperacillin adaptation in this study, but occurs during ciprofloxacin adaptation as well when the historical genomic context is suitable. When all four large deletions are compared, the overlap region is ~95 kbp (encoding ~77 genes). Bacteria are known to shed large portions of their genome as they adapt to a niche environment, suggesting that they streamline their DNA and get rid of non-essential genes that do not contribute to an enhanced fitness in the environment [8]. In pathogens such as *P. aeruginosa*, selective genome reduction has been seen in clinical isolates as bacteria adapt to the niche environment of the host [9]. It is interesting that we were able to also recapitulate similar genomic deletions through experimental evolution.

During the adaptive evolution, a visually observable phenotype was observed for all the lineages that had the large chromosomal deletion. These lineages produced the brown secreted pigment pyomelanin. The hyperproduction of pyomelanin observed here is attributed to the inactivation of the *hmgA* gene in the homogentisate pathway, which is part of the larger tyrosine catabolism pathway. *hmgA* codes for homogentisate-1,2-dioxygenase, which converts homogentisate to 4-maleylacetoacetate. When *hmgA* is non-functional, homogentisate gets secreted, auto-oxidizes, and self-polymerizes to form pyomelanin [10,11]. Indeed, in all the lineages that had the large chromosomal deletion, *hmgA* was one of the genes in the deletion. Because *hmgA* is only one of many genes lost in the large deletion, it is unclear if there is an actual selective advantage for the pyomelanin phenotype, or if the pyomelanin phenotype is a "side-effect" of losing one or more genes in the deletion that actually does confer a selective advantage. There have been some studies that suggest that pyomelanin production by *P. aeruginosa* protects the bacteria against oxidative stress and contributes to increased persistence in a mouse model of chronic lung infection [10]. Clinical isolates of *P. aeruginosa* that produce pyomelanin have been well documented in the literature [12], but to the best of the authors' knowledge, there has only been two studies that attribute the pyomelanin production in clinical isolates to loss of *hmgA* as part of a large chromosomal deletion, similar to those seen in this experimental evolution study [13,14]. This result demonstrates how this experimental evolution study has recapitulated genotypes and phenotypes encountered clinically.

References

1. Leiby N, Marx CJ. Metabolic Erosion Primarily Through Mutation Accumulation, and Not Tradeoffs, Drives Limited Evolution of Substrate Specificity in Escherichia coli. Moran NA, editor. PLoS Biol. Public Library of Science; 2014;12: e1001789. doi:10.1371/journal.pbio.1001789

2. Morita Y, Murata T, Mima T, Shiota S, Kuroda T, Mizushima T, et al. Induction of mexCD-oprJ operon for a multidrug efflux pump by disinfectants in wild-type Pseudomonas aeruginosa PAO1. J Antimicrob Chemother. 2003;51: 991–994. doi:10.1093/jac/dkg173

3. Wong A, Rodrigue N, Kassen R. Genomics of Adaptation during Experimental Evolution of the Opportunistic Pathogen Pseudomonas aeruginosa. PLoS Genet. 2012;8: e1002928. doi:10.1371/journal.pgen.1002928

4. Purssell A, Poole K. Functional characterization of the NfxB repressor of the mexCD-oprJ multidrug efflux operon of Pseudomonas aeruginosa. Microbiology. 2013;159: 2058–2073. doi:10.1099/mic.0.069286-0

5. Hirai K, Suzue S, Irikura T, Iyobe S, Mitsuhashi S. Mutations producing resistance to norfloxacin in Pseudomonas aeruginosa. Antimicrob Agents Chemother. 1987;31: 582–586. doi:10.1128/AAC.31.4.582

6. Poole K, Gotoh N, Tsujimoto H, Zhao Q, Wada A, Yamasaki T, et al. Overexpression of the mexC-mexD-oprJ efflux operon in nfxB-type multidrug-resistant strains of Pseudomonas aeruginosa. Mol Microbiol. 1996;21: 713–724. doi:Doi 10.1046/J.1365-2958.1996.281397.X

7. Starr LM, Fruci M, Poole K. Pentachlorophenol Induction of the Pseudomonas aeruginosa mexAB-oprM Efflux Operon: Involvement of Repressors NalC and MexR and the Antirepressor ArmR. PLoS One. 2012;7: 1–9. doi:10.1371/journal.pone.0032684

8. Moran NA. Microbial Minimalism: Genome Reduction in Bacterial Pathogens. Cell. 2002;108: 583–586. doi:10.1016/S0092-8674(02)00665-7

9. Rau MH, Marvig RL, Ehrlich GD, Molin S, Jelsbak L. Deletion and acquisition of genomic content during early stage adaptation of Pseudomonas aeruginosa to a human host environment. Environ Microbiol. 2012;14: 2200–2211. doi:10.1111/j.1462-2920.2012.02795.x

10. Rodríguez-Rojas A, Mena A, Martín S, Borrell N, Oliver A, Blázquez J. Inactivation of the hmgA gene of Pseudomonas aeruginosa leads to pyomelanin hyperproduction, stress resistance and increased persistence in chronic lung infection. Microbiology. 2009;155: 1050–1057. doi:10.1099/mic.0.024745-0

11. Ketelboeter LM, Potharla VY, Bardy SL. NTBC Treatment of the Pyomelanogenic Pseudomonas aeruginosa Clinical Isolate PA1111 Inhibits Pigment Production and Increases Sensitivity to Oxidative Stress. Curr Microbiol. 2014;69: 343–348. doi:10.1007/s00284-014-0593-9

12. Yabuuchi E, Ohyama A. Characterization of “Pyomelanin” -Producing Strains of Pseudomonas aeruginosa. Int J Syst Bacteriol. 1972;22: 53–64. doi:10.1099/00207713-22-2-53

13. Ernst RK, D’Argenio DA, Ichikawa JK, Bangera MG, Selgrade S, Burns JL, et al. Genome mosaicism is conserved but not unique in Pseudomonas aeruginosa isolates from the airways of young children with cystic fibrosis. Environ Microbiol. 2003;5: 1341–1349. doi:10.1111/j.1462-2920.2003.00518.x

14. Hocquet D, Petitjean M, Rohmer L, Valot B, Kulasekara HD, Bedel E, et al. Pyomelanin-producing Pseudomonas aeruginosa selected during chronic infections have a large chromosomal deletion which confers resistance to pyocins. Environ Microbiol. 2016;18: 3482–3493. doi:10.1111/1462-2920.13336
