# Supplementary material for: History of antibiotic adaptation influences microbial evolutionary dynamics during subsequent treatment
Source: PLoS Biol. 2017 Aug 8;15(8):e2001586. doi: 10.1371/journal.pbio.2001586 (PMC5549691; doi:10.1371/journal.pbio.2001586)
Supplement: S2 Text — This file contains the calculations for all statistical tests performed in this study. (PDF) [file pbio.2001586.s002.pdf]

Supplementary Materials (S2 Text) for

History of Antibiotic Adaptation Influences Microbial Evolutionary Dynamics During Subsequent Treatment

Phillip Yen and Jason Papin*

*Corresponding author. E-mail: papin@virginia.edu

This file contains the calculations for all statistical tests performed in this study:

Drug order-specific effects (Fig 3 of main text)
- Fig 3A (top) (PIP^R lineages)
- Fig 3A (middle) (TOB^R lineages)
- Fig 3A (bottom) (CIP^R lineages)
- Fig 3A (bottom) for Day 25 (CIP^R lineages)
- Fig 3B
- Fig 3C (top)
- Fig 3C (middle)
- Fig 3C (bottom)

Collateral sensitivity (S4 Fig)
- S4A Fig (left)
- S4A Fig (right)
- S4B Fig
- S4C Fig

Piperacillin-resistant clinical isolates (Fig 7 of main text)
- Clinical Isolate #1
- Clinical Isolate #2
- Clinical Isolate #3

Hocquet Isolates (Fig 8 of main text)
- A_{WT} vs. A_{PM}
- B_{WT} vs. B_{PM}
- C_{WT} vs. C_{PM}
- D_{WT} vs. D_{PM}

Drug order-specific effects (Fig 3 of main text)

Fig 3A (top) (PIP^R lineages)

$\log_2 MIC_{PIP}$ of:	Replicate 1	2	3	4
Day 1 PIP^R	2	3	3	2
Day 20 PIP^R	9	7	7	7
Day 40 PIP^RLB	8	6	5	6
Day 40 $PIP^R TOB^R$	8	7	6	5
Day 40 $PIP^R CIP^R$	7	0	4	4

A one-way ANOVA is performed (`anova1` in MATLAB) on the $\log_2 MIC_{PIP}$ values of these lineages:

ANOVA Table

```
Source       SS      df       MS         F      Prob>F
---------------------------------------------------------
Columns     69.7      4    17.425     6.79    0.0025
Error       38.5     15     2.5667
Total      108.2     19
```

We also performed the non-parametric Kruskal-Wallis test (in R):

```
> kruskal.test(value ~ lineage, data = Fig3AtopPIPR)

        Kruskal-Wallis rank sum test

data:  value by lineage
Kruskal-Wallis chi-squared = 12.063, df = 4, p-value = 0.01689
```

With an **ANOVA p-value of 0.0025**, and a **Kruskal-Wallis p-value of 0.01689,** the treatments are significantly different at the alpha=0.05 level, and we continue with multiple comparisons testing with the Tukey HSD test (`multcompare` in MATLAB), and the Dunn's test (`PMCMR` package in R).

$\log_2$ MIC_{PIP} of:		lower bound	difference between means	upper bound	Tukey HSD p-value	Dunn's Test p-value
Day 1 PIP^R	Day 20 PIP^R	-8.50	-5.00	-1.50	0.0039	0.0021
Day 1 PIP^R	Day 40 PIP^RLB	-7.25	-3.75	-0.25	0.0329	0.0344
Day 1 PIP^R	Day 40 $PIP^R TOB^R$	-7.50	-4.00	-0.50	0.0216	0.0184
**Day 1 PIP^R**	**Day 40 $PIP^R CIP^R$**	**-4.75**	**-1.25**	**2.25**	**0.8022**	0.3647
**Day 20 PIP^R**	**Day 40 PIP^RLB**	**-2.25**	**1.25**	**4.75**	**0.8022**	0.3337
**Day 20 PIP^R**	**Day 40 $PIP^R TOB^R$**	**-2.50**	**1.00**	**4.50**	**0.8989**	0.4684
**Day 20 PIP^R**	**Day 40 $PIP^R CIP^R$**	**0.25**	**3.75**	**7.25**	**0.0329**	0.0296
Day 40 PIP^RLB	Day 40 $PIP^R TOB^R$	-3.75	-0.25	3.25	0.9994	0.8090
Day 40 PIP^RLB	Day 40 $PIP^R CIP^R$	-1.00	2.50	6.00	0.2296	0.2269
Day 40 $PIP^R TOB^R$	Day 40 $PIP^R CIP^R$	-0.75	2.75	6.25	0.1612	0.1470

Fig 3A (middle) (TOB^R lineages)

$\log_2 MIC_{TOB}$ of:	Replicate 1	2	3	4
Day 1 TOB^R	1	1	1	1
Day 20 TOB^R	7	6	8	7
Day 40 TOB^RLB	4	3	3	4
Day 40 $TOB^R PIP^R$	5	6	4	6
Day 40 $TOB^R CIP^R$	3	3	3	4

A one-way ANOVA is performed (`anova1` in MATLAB) on the $\log_2 MIC_{TOB}$ values of these lineages:

```
                        ANOVA Table
Source      SS     df      MS          F         Prob>F
-----------------------------------------------------------
Columns    81.5     4    20.375     47.02    2.59048e-008
Error       6.5    15     0.4333
Total      88      19
```

We also performed the non-parametric Kruskal-Wallis test (in R):

```
> kruskal.test(value ~ lineage, data = Fig3AmiddleTOBR)

        Kruskal-Wallis rank sum test

data:  value by lineage
Kruskal-Wallis chi-squared = 17.303, df = 4, p-value = 0.001687
```

With an **ANOVA p-value of 2.6×10^{-8}** and a **Kruskal-Wallis p-value of 0.001687,** the treatments are significantly different at the alpha=0.05 level, and we continue with multiple comparisons testing with the Tukey HSD test (`multcompare` in MATLAB), and the Dunn's test (`PMCMR` package in R).

$\log_2 MIC_{TOB}$ of:		lower bound	difference between means	upper bound	Tukey HSD p-value	Dunn's Test p-value
Day 1 TOB^R	Day 20 TOB^R	-7.44	-6.00	-4.56	2.46E-08	0.00013
Day 1 TOB^R	Day 40 TOB^RLB	-3.94	-2.50	-1.06	0.0006	0.10068
Day 1 TOB^R	Day 40 $TOB^R PIP^R$	-5.69	-4.25	-2.81	1.44E-06	0.00388
Day 1 TOB^R	Day 40 $TOB^R CIP^R$	-3.69	-2.25	-0.81	0.0017	0.17132
**Day 20 TOB^R**	**Day 40 TOB^RLB**	**2.06**	**3.50**	**4.94**	**1.58E-05**	0.02861
**Day 20 TOB^R**	**Day 40 $TOB^R PIP^R$**	**0.31**	**1.75**	**3.19**	**0.0139**	0.34600
**Day 20 TOB^R**	**Day 40 $TOB^R CIP^R$**	**2.31**	**3.75**	**5.19**	**6.86E-06**	0.01380
**Day 40 TOB^RLB**	**Day 40 $TOB^R PIP^R$**	**-3.19**	**-1.75**	**-0.31**	**0.0139**	0.21263
**Day 40 TOB^RLB**	**Day 40 $TOB^R CIP^R$**	**-1.19**	**0.25**	**1.69**	**0.9819**	0.78440
**Day 40 $TOB^R PIP^R$**	**Day 40 $TOB^R CIP^R$**	**0.56**	**2.00**	**3.44**	**0.0049**	0.12852

Fig 3A (bottom) (CIP^R lineages)

log_2 MIC_{CIP} of:	Replicate 1	2	3	4
Day 1 CIP^R	-1	-2	-3	-1
Day 20 CIP^R	5	4	4	4
Day 40 CIP^RLB	3	4	-1	2
Day 40 CIP^RPIP^R	1	1	2	1
Day 40 CIP^RTOB^R	2	-1	2	-1

A one-way ANOVA is performed (`anova1` in MATLAB) on the log_2 MIC_{CIP} values of these lineages:

```
ANOVA Table

Source        SS      df      MS          F     Prob>F
-------------------------------------------------------
Columns     76.5       4    19.125     10.53    0.0003
Error       27.25     15     1.8167
Total      103.75     19
```

We also performed the non-parametric Kruskal-Wallis test (in R):

```
> kruskal.test(value ~ lineage, data = Fig3AbottomCIPR)

        Kruskal-Wallis rank sum test

data:  value by lineage
Kruskal-Wallis chi-squared = 13.966, df = 4, p-value = 0.007406
```

With an **ANOVA p-value of 0.0003** and a **Kruskal-Wallis p-value of 0.007406**, the treatments are significantly different at the alpha=0.05 level, and we continue with multiple comparisons testing with the Tukey HSD test (`multcompare` in MATLAB), and the Dunn's test (`PMCMR` package in R).

log_2 MIC_{CIP} of:		lower bound	difference between means	upper bound	Tukey HSD p-value	Dunn's Test p-value
Day 1 CIP^R	Day 20 CIP^R	-8.94	-6.00	-3.06	0.0001	0.0003
Day 1 CIP^R	Day 40 CIP^RLB	-6.69	-3.75	-0.81	0.0099	0.0245
Day 1 CIP^R	Day 40 CIP^RPIP^R	-5.94	-3.00	-0.06	0.0447	0.1073
Day 1 CIP^R	Day 40 CIP^RTOB^R	-5.19	-2.25	0.69	0.1799	0.1812
**Day 20 CIP^R**	**Day 40 CIP^RLB**	**-0.69**	**2.25**	**5.19**	**0.1799**	0.1715
**Day 20 CIP^R**	**Day 40 CIP^RPIP^R**	**0.06**	**3.00**	**5.94**	**0.0447**	0.0449
**Day 20 CIP^R**	**Day 40 CIP^RTOB^R**	**0.81**	**3.75**	**6.69**	**0.0099**	0.0227
**Day 40 CIP^RLB**	**Day 40 CIP^RPIP^R**	**-2.19**	**0.75**	**3.69**	**0.9305**	0.5234
**Day 40 CIP^RLB**	**Day 40 CIP^RTOB^R**	**-1.44**	**1.50**	**4.44**	**0.5345**	0.3620
Day 40 CIP^RPIP^R	Day 40 CIP^RTOB^R	-2.19	0.75	3.69	0.9305	0.7845

Fig 3A (bottom) for Day 25 (CIP^R lineages)

log$_2$ MIC$_{CIP}$ of:	Replicate			
	1	2	3	4
Day 1 CIP^R	-1	-2	-3	-1
Day 20 CIP^R	5	4	4	4
Day 25 CIP^{R}LB	2	5	2	3
Day 25 CIP^{R}PIP^R	0	-1	2	-1
Day 25 CIP^{R}TOB^R	6	3	4	4

A one-way ANOVA is performed (`anova1` in MATLAB) on the log$_2$ MIC$_{CIP}$ values of these lineages:

```
                         ANOVA Table
Source       SS      df      MS        F        Prob>F
--------------------------------------------------------
Columns    116.7      4    29.175    21.61    4.39215e-006
Error       20.25    15     1.35
Total      136.95    19
```

We also performed the non-parametric Kruskal-Wallis test (in R):

```
> kruskal.test(value ~ lineage, data = Fig3AbottomCIPR_day25)

        Kruskal-Wallis rank sum test

data:  value by lineage
Kruskal-Wallis chi-squared = 15.179, df = 4, p-value = 0.004345
```

With an **ANOVA p-value of 4.4x10^{-6}** and a **Kruskal-Wallis p-value of 0.004345**, the treatments are significantly different at the alpha=0.05 level, and we continue with multiple comparisons testing with the Tukey HSD test (`multcompare` in MATLAB), and the Dunn's test (`PMCMR` package in R).

log$_2$ MIC$_{CIP}$ of:		lower bound	difference between means	upper bound	Tukey HSD p-value	Dunn's Test p-value
Day 1 CIP^R	Day 20 CIP^R	-8.54	-6.00	-3.46	2.23E-05	0.0018
Day 1 CIP^R	Day 25 CIP^{R}LB	-7.29	-4.75	-2.21	0.0003	0.0292
Day 1 CIP^R	Day 25 CIP^{R}PIP^R	-4.29	-1.75	0.79	0.2581	0.4309
Day 1 CIP^R	Day 25 CIP^{R}TOB^R	-8.54	-6.00	-3.46	2.23E-05	0.0027
**Day 20 CIP^R**	**Day 25 CIP^{R}LB**	**-1.29**	**1.25**	**3.79**	**0.5651**	0.3477
**Day 20 CIP^R**	**Day 25 CIP^{R}PIP^R**	**1.71**	**4.25**	**6.79**	**0.0009**	0.0197
**Day 20 CIP^R**	**Day 25 CIP^{R}TOB^R**	**-2.54**	**0.00**	**2.54**	**1.0000**	0.9036
Day 25 CIP^{R}LB	Day 25 CIP^{R}PIP^R	0.46	3.00	5.54	0.0171	0.1635
Day 25 CIP^{R}LB	Day 25 CIP^{R}TOB^R	-3.79	-1.25	1.29	0.5651	0.4134
Day 25 CIP^{R}PIP^R	Day 25 CIP^{R}TOB^R	-6.79	-4.25	-1.71	9.19E-04	0.0270

Fig 3B

$\log_2$ MIC_{TOB} of:	Replicate			
	**1**	**2**	**3**	**4**
Day 20 PIP^R	1	1	2	3
Day 40 PIP^RTOB^R	4	3	4	7
Day 1 TOB^R	1	1	1	1
Day 20 TOB^R	7	6	8	7

A one-way ANOVA is performed (`anova1` in MATLAB) on the $\log_2$ MIC_{TOB} values of these lineages:

ANOVA Table

```
Source        SS       df      MS         F       Prob>F
-------------------------------------------------------------
Columns     90.188      3   30.0625    26.24   1.48008e-005
Error       13.75      12    1.1458
Total      103.938     15
```

We also performed the non-parametric Kruskal-Wallis test (in R):

```
> kruskal.test(value ~ lineage, data = Fig3B)

        Kruskal-Wallis rank sum test

data:  value by lineage
Kruskal-Wallis chi-squared = 12.987, df = 3, p-value = 0.004665
```

With an **ANOVA p-value of 1.48×10^{-5}** and a **Kruskal-Wallis p-value of 0.004665**, the treatments are significantly different at the alpha=0.05 level, and we continue with multiple comparisons testing with the Tukey HSD test (`multcompare` in MATLAB), and the Dunn's test (`PMCMR` package in R).

$\log_2$ MIC_{TOB} of:		lower bound	difference between means	upper bound	Tukey HSD p-value	Dunn's Test p-value
Day 20 PIP^R	Day 40 PIP^RTOB^R	-5.00	-2.75	-0.50	0.0157	0.1077
**Day 20 PIP^R**	**Day 1 TOB^R**	**-1.50**	**0.75**	**3.00**	**0.7573**	0.5149
Day 20 PIP^R	Day 20 TOB^R	-7.50	-5.25	-3.00	8.04E-05	0.0103
Day 40 PIP^RTOB^R	Day 1 TOB^R	1.25	3.50	5.75	0.0028	0.0238
**Day 40 PIP^RTOB^R**	**Day 20 TOB^R**	**-4.75**	**-2.50**	**-0.25**	**0.0280**	0.3383
Day 1 TOB^R	Day 20 TOB^R	-8.25	-6.00	-3.75	2.14E-05	0.0013

Fig 3C (top)

$\log_2$ MIC_{PIP} of:	Replicate			
	**1**	**2**	**3**	**4**
Day 1 PIP^R	2	3	3	2
Day 40 PIP^RCIP^R	7	0	4	4
Day 1 CIP^R	3	4	3	4
Day 40 CIP^RPIP^R	9	7	8	8

A one-way ANOVA is performed (`anova1` in MATLAB) on the $\log_2$ MIC_{PIP} values of these lineages:

ANOVA Table

```
Source        SS       df      MS        F     Prob>F
-----------------------------------------------------
Columns    71.1875     3    23.7292    9.9    0.0014
Error      28.75      12     2.3958
Total      99.9375    15
```

We also performed the non-parametric Kruskal-Wallis test (in R):

```
> kruskal.test(value ~ lineage, data = Fig3Ctop)

        Kruskal-Wallis rank sum test

data:  value by lineage
Kruskal-Wallis chi-squared = 10.211, df = 3, p-value = 0.01685
```

With an **ANOVA p-value of 0.0014** and a **Kruskal-Wallis p-value of 0.01685**, the treatments are significantly different at the alpha=0.05 level, and we continue with multiple comparisons testing with the Tukey HSD test (`multcompare` in MATLAB), and the Dunn's test (`PMCMR` package in R).

$\log_2$ MIC_{PIP} of:		lower bound	difference between means	upper bound	Tukey HSD p-value	Dunn's Test p-value
Day 1 PIP^R	Day 40 PIP^RCIP^R	-4.50	-1.25	2.00	0.6720	0.2126
**Day 1 PIP^R**	**Day 1 CIP^R**	**-4.25**	**-1.00**	**2.25**	**0.7981**	0.2902
Day 1 PIP^R	Day 40 CIP^RPIP^R	-8.75	-5.50	-2.25	0.0015	0.0017
Day 40 PIP^RCIP^R	Day 1 CIP^R	-3.00	0.25	3.50	0.9956	0.8502
**Day 40 PIP^RCIP^R**	**Day 40 CIP^RPIP^R**	**-7.50**	**-4.25**	**-1.00**	**0.0101**	0.0589
Day 1 CIP^R	Day 40 CIP^RPIP^R	-7.75	-4.50	-1.25	0.0068	0.0377

Fig 3C (middle)

$\log_2$ MIC$_{PIP}$ of:	Replicate			
	1	2	3	4
Day 1 TOB^R	3	3	2	3
Day 40 TOB^{R}CIP^R	1	2	1	2
Day 1 CIP^R	3	4	3	4
Day 40 CIP^{R}TOB^R	3	4	3	4

A one-way ANOVA is performed (`anova1` in MATLAB) on the $\log_2$ MIC$_{PIP}$ values of these lineages:

```
ANOVA Table
Source      SS        df     MS        F      Prob>F
----------------------------------------------------
Columns   10.6875      3   3.5625    11.4    0.0008
Error      3.75       12   0.3125
Total     14.4375     15
```

We also performed the non-parametric Kruskal-Wallis test (in R):

```
> kruskal.test(value ~ lineage, data = Fig3Cmiddle)

        Kruskal-Wallis rank sum test

data:  value by lineage
Kruskal-Wallis chi-squared = 10.788, df = 3, p-value = 0.01293
```

With an **ANOVA p-value of 0.0008** and a **Kruskal-Wallis p-value of 0.01293**, the treatments are significantly different at the alpha=0.05 level, and we continue with multiple comparisons testing with the Tukey HSD test (`multcompare` in MATLAB), and the Dunn's test (`PMCMR` package in R).

$\log_2$ MIC$_{PIP}$ of:		lower bound	difference between means	upper bound	Tukey HSD p-value	Dunn's Test p-value
Day 1 TOB^R	Day 40 TOB^{R}CIP^R	0.08	1.25	2.42	0.0357	0.1166
**Day 1 TOB^R**	**Day 1 CIP^R**	**-1.92**	**-0.75**	**0.42**	**0.2795**	0.2093
Day 1 TOB^R	Day 40 CIP^{R}TOB^R	-1.92	-0.75	0.42	0.2795	0.2093
Day 40 TOB^{R}CIP^R	Day 1 CIP^R	-3.17	-2.00	-0.83	0.0014	0.0047
**Day 40 TOB^{R}CIP^R**	**Day 40 CIP^{R}TOB^R**	**-3.17**	**-2.00**	**-0.83**	**0.0014**	0.0047
Day 1 CIP^R	Day 40 CIP^{R}TOB^R	-1.17	0.00	1.17	1.0000	1.000

Fig 3C (bottom)

**log_2 MIC_{CIP} of:**	**Replicate**			
	**1**	**2**	**3**	**4**
Day 1 TOB^R	-1	-2	-2	-2
Day 40 TOB^RCIP^R	4	3	3	3
Day 1 CIP^R	-1	-2	-3	-1
Day 40 CIP^RTOB^R	2	-1	2	-1

A one-way ANOVA is performed (`anova1` in MATLAB) on the log_2 MIC_{CIP} values of these lineages:

```
                                 ANOVA Table
Source       SS       df      MS          F          Prob>F
-------------------------------------------------------------
Columns    67.6875     3   22.5625      20.43     5.23348e-005
Error      13.25      12    1.1042
Total      80.9375    15
```

We also performed the non-parametric Kruskal-Wallis test (in R):

```
> kruskal.test(value ~ lineage, data = Fig3Cbottom)

        Kruskal-Wallis rank sum test

data:  value by lineage
Kruskal-Wallis chi-squared = 11.901, df = 3, p-value = 0.00773
```

With an **ANOVA p-value of 5.23×10^{-5}** and a **Kruskal-Wallis p-value of 0.00773**, the treatments are significantly different at the alpha=0.05 level, and we continue with multiple comparisons testing with the Tukey HSD test (`multcompare` in MATLAB), and the Dunn's test (`PMCMR` package in R).

log_2 MIC_{CIP} of:		lower bound	difference between means	upper bound	Tukey HSD p-value	Dunn's Test p-value
Day 1 TOB^R	Day 40 TOB^RCIP^R	-7.21	-5.00	-2.79	1.08E-04	0.0026
**Day 1 TOB^R**	**Day 1 CIP^R**	**-2.21**	**0.00**	**2.21**	**1.0000**	0.8788
Day 1 TOB^R	Day 40 CIP^RTOB^R	-4.46	-2.25	-0.04	0.0451	0.1180
Day 40 TOB^RCIP^R	Day 1 CIP^R	2.79	5.00	7.21	1.08E-04	0.0042
**Day 40 TOB^RCIP^R**	**Day 40 CIP^RTOB^R**	**0.54**	**2.75**	**4.96**	**0.0139**	0.1474
Day 1 CIP^R	Day 40 CIP^RTOB^R	-4.46	-2.25	-0.04	0.0451	0.1584

Collateral sensitivity (S4 Fig)

S4A Fig (left)

**$\log_2$ MIC_{TOB} of:**	**Replicate**			
	**1**	**2**	**3**	**4**
Day 1 CIP^R	2	3	3	2
Day 20 CIP^R	-1	-2	0	-2
Day 1 Control	1	1	1	1
Day 20 Control	1	1	2	2

A one-way ANOVA is performed (`anova1` in MATLAB) on the $\log_2$ MIC_{TOB} values of these lineages:

ANOVA Table

Source	SS	df	MS	F	Prob>F
Columns	30.1875	3	10.0625	25.42	1.74077e-005
Error	4.75	12	0.3958		
Total	34.9375	15			

We also performed the non-parametric Kruskal-Wallis test (in R):

```
> kruskal.test(value ~ lineage, data = FigS4Aleft)

        Kruskal-Wallis rank sum test

data:  value by lineage
Kruskal-Wallis chi-squared = 13.175, df = 3, p-value = 0.004272
```

With an **ANOVA p-value of 1.74×10^{-5}** and a **Kruskal-Wallis p-value of 0.004272**, the treatments are significantly different at the alpha=0.05 level, and we continue with multiple comparisons testing with the Tukey HSD test (`multcompare` in MATLAB), and the Dunn's test (`PMCMR` package in R).

$\log_2$ MIC_{TOB} of:		lower bound	difference between means	upper bound	Tukey HSD p-value	Dunn's Test p-value
**Day 1 CIP^R**	**Day 20 CIP^R**	**2.43**	**3.75**	**5.07**	**1.14E-05**	0.0004
Day 1 CIP^R	Day 1 Control	0.18	1.50	2.82	0.0248	0.0454
Day 1 CIP^R	Day 20 Control	-0.32	1.00	2.32	0.1656	0.2181
Day 20 CIP^R	Day 1 Control	-3.57	-2.25	-0.93	0.0014	0.1237
Day 20 CIP^R	Day 20 Control	-4.07	-2.75	-1.43	2.39E-04	0.0209
**Day 1 Control**	**Day 20 Control**	**-1.82**	**-0.50**	**0.82**	**0.6825**	0.4415

S4A Fig (right)

log$_2$ MIC$_{PIP}$ of:	Replicate			
	1	2	3	4
Day 1 CIP^R	3	4	3	4
Day 20 CIP^R	2	2	1	2
Day 1 Control	2	3	2	2
Day 20 Control	3	3	4	3

A one-way ANOVA is performed (`anova1` in MATLAB) on the log$_2$ MIC$_{PIP}$ values of these lineages:

```
                                  ANOVA Table
Source        SS       df      MS         F      Prob>F
--------------------------------------------------
Columns     8.1875      3   2.72917   10.08    0.0013
Error       3.25       12   0.27083
Total      11.4375     15
```

We also performed the non-parametric Kruskal-Wallis test (in R):

```
> kruskal.test(value ~ lineage, data = FigS4Aright)

        Kruskal-Wallis rank sum test

data:  value by lineage
Kruskal-Wallis chi-squared = 11.454, df = 3, p-value = 0.009507
```

With an **ANOVA p-value of 0.009507** and a **Kruskal-Wallis p-value of 0.009507**, the treatments are significantly different at the alpha=0.05 level, and we continue with multiple comparisons testing with the Tukey HSD test (`multcompare` in MATLAB), and the Dunn's test (`PMCMR` package in R).

log$_2$ MIC$_{PIP}$ of:		lower bound	difference between means	upper bound	Tukey HSD p-value	Dunn's Test p-value
**Day 1 CIP^R**	**Day 20 CIP^R**	**0.66**	**1.75**	**2.84**	**0.0023**	0.0041
Day 1 CIP^R	Day 1 Control	0.16	1.25	2.34	0.0237	0.0337
Day 1 CIP^R	Day 20 Control	-0.84	0.25	1.34	0.9029	0.7233
Day 20 CIP^R	Day 1 Control	-1.59	-0.50	0.59	0.5461	0.4549
Day 20 CIP^R	Day 20 Control	-2.59	-1.50	-0.41	0.0072	0.0118
**Day 1 Control**	**Day 20 Control**	**-2.09**	**-1.00**	**0.09**	**0.0769**	0.0767

S4B Fig

$\log_2$ MIC_{TOB} of:	Replicate			
	1	2	3	4
Day 1 Control	1	1	1	1
Day 20 PIP^R	1	1	2	3
Day 20 Control	1	1	2	2
Day 40 $PIP^R CIP^R$	0	0	0	0

A one-way ANOVA is performed (`anova1` in MATLAB) on the $\log_2$ MIC_{TOB} values of these lineages:

```
                                   ANOVA Table
Source       SS       df      MS         F      Prob>F
-------------------------------------------------
Columns     7.1875     3   2.39583   7.67    0.004
Error       3.75      12   0.3125
Total      10.9375    15
```

We also performed the non-parametric Kruskal-Wallis test (in R):

```
> kruskal.test(value ~ lineage, data = FigS4B)

        Kruskal-Wallis rank sum test

data:  value by lineage
Kruskal-Wallis chi-squared = 11.16, df = 3, p-value = 0.01089
```

With an **ANOVA p-value of 0.004** and a **Kruskal-Wallis p-value of 0.01089**, the treatments are significantly different at the alpha=0.05 level, and we continue with multiple comparisons testing with the Tukey HSD test (`multcompare` in MATLAB), and the Dunn's test (`PMCMR` package in R).

$\log_2$ MIC_{TOB} of:		lower bound	difference between means	upper bound	Tukey HSD p-value	Dunn's Test p-value
Day 1 Control	Day 20 PIP^R	-1.92	-0.75	0.42	0.2795	0.297
**Day 1 Control**	**Day 20 Control**	**-1.67**	**-0.50**	**0.67**	**0.6005**	0.377
Day 1 Control	Day 40 $PIP^R CIP^R$	-0.17	1.00	2.17	0.1052	0.054
Day 20 PIP^R	Day 20 Control	-0.92	0.25	1.42	0.9196	0.872
**Day 20 PIP^R**	**Day 40 $PIP^R CIP^R$**	**0.58**	**1.75**	**2.92**	**0.0040**	0.003
Day 20 Control	Day 40 $PIP^R CIP^R$	0.33	1.50	2.67	0.0118	0.005

S4C Fig

$\log_2 MIC_{PIP}$ of:	Replicate			
	1	2	3	4
Day 1 Control	2	3	2	2
Day 20 TOB^R	3	3	3	4
Day 20 Control	3	3	4	3
Day 40 TOB^{R}CIP^R	1	2	1	2

A one-way ANOVA is performed (`anova1` in MATLAB) on the $\log_2 MIC_{PIP}$ values of these lineages:

```
                                      ANOVA Table
Source        SS       df      MS         F     Prob>F
-------------------------------------------------
Columns     8.6875     3   2.89583   10.69   0.001
Error       3.25      12   0.27083
Total      11.9375    15
```

We also performed the non-parametric Kruskal-Wallis test (in R):

```
> kruskal.test(value ~ lineage, data = FigS4C)

        Kruskal-Wallis rank sum test

data:  value by lineage
Kruskal-Wallis chi-squared = 11.53, df = 3, p-value = 0.009178
```

With an **ANOVA p-value of 0.001** and a **Kruskal-Wallis p-value of 0.009178**, the treatments are significantly different at the alpha=0.05 level, and we continue with multiple comparisons testing with the Tukey HSD test (`multcompare` in MATLAB), and the Dunn's test (`PMCMR` package in R).

$\log_2 MIC_{PIP}$ of:		lower bound	difference between means	upper bound	Tukey HSD p-value	Dunn's Test p-value
Day 1 Control	Day 20 TOB^R	-2.09	-1.00	0.09	0.0769	0.0758
**Day 1 Control**	**Day 20 Control**	**-2.09**	**-1.00**	**0.09**	**0.0769**	0.0758
Day 1 Control	Day 40 TOB^{R}CIP^R	-0.34	0.75	1.84	0.2280	0.3049
Day 20 TOB^R	Day 20 Control	-1.09	0.00	1.09	1.0000	1.000
**Day 20 TOB^R**	**Day 40 TOB^{R}CIP^R**	**0.66**	**1.75**	**2.84**	**0.0023**	0.0051
Day 20 Control	Day 40 TOB^{R}CIP^R	0.66	1.75	2.84	0.0023	0.0051

Piperacillin-resistant clinical isolates (Fig 7 of main text)

Clinical Isolate #1

	Raw values								
	log₂(MIC Piperacillin) (μg/ml)								
	Clinical Isolate 1								
	Evolved to tobramycin			Evolved to ciprofloxacin			Evolved to LB		
	1	2	3	1	2	3	1	2	3
Day									
1	6	7	5	6	5	6	7	7	7
10	2	7	5	5	6	6	8	8	8

	Normalized values								
	log₂(MIC Piperacillin) (μg/ml)								
	Clinical Isolate 1								
	Evolved to tobramycin			Evolved to ciprofloxacin			Evolved to LB		
	1	2	3	1	2	3	1	2	3
Day									
1	0.00	1.00	-1.00	0.33	-0.67	0.33	0.00	0.00	0.00
10	-4.00	1.00	-1.00	-0.67	0.33	0.33	1.00	1.00	1.00

The normalized values are calculated by subtracting the average of the Day 1 $\log_2$ MIC_{PIP} values from the raw values. More explicitly,

Normalized $\log_2$ MIC_{PIP} of Day 1 Clinical Isolate #1 evolved to tobramycin:
`[6 7 5] - mean([6 7 5]) = [0 1 -1]`

Normalized $\log_2$ MIC_{PIP} of Day 10 Clinical Isolate #1 evolved to tobramycin:
`[2 7 5] - mean([6 7 5]) = [-4 1 -1]`

Normalized $\log_2$ MIC_{PIP} of Day 1 Clinical Isolate #1 evolved to ciprofloxacin:
`[6 5 6] - mean([6 5 6]) = [0.33 -0.67 0.33]`

Normalized $\log_2$ MIC_{PIP} of Day 10 Clinical Isolate #1 evolved to ciprofloxacin:
`[5 6 6] - mean([6 5 6]) = [-0.67 0.33 0.33]`

Normalized $\log_2$ MIC_{PIP} of Day 1 Clinical Isolate #1 evolved to LB:
`[7 7 7] - mean([7 7 7]) = [0 0 0]`

Normalized $\log_2$ MIC_{PIP} of Day 10 Clinical Isolate #1 evolved to LB:

```
[8 8 8] - mean([7 7 7]) = [1 1 1]
```

Similar calculations are done for Clinical Isolates #2 and #3.

Next, a one-way ANOVA is performed (`anova1` in MATLAB) on the normalized Day 10 $\log_2$ MIC_{PIP} values:

ANOVA Table

```
Source        SS      df      MS        F      Prob>F
-------------------------------------------------
Columns     8.2222    2    4.11111    1.85    0.2367
Error      13.3333    6    2.22222
Total      21.5556    8
```

We also performed the non-parametric Kruskal-Wallis test (in R):

```
> kruskal.test(value ~ lineage, data = PIPRCI1)

        Kruskal-Wallis rank sum test

data:  value by lineage
Kruskal-Wallis chi-squared = 4.1835, df = 2, p-value = 0.1235
```

With an **ANOVA p-value of 0.2367** and a **Kruskal-Wallis p-value of 0.1235**, the treatments are not significantly different at the alpha=0.05 level, and we do not continue with multiple comparisons testing.

Clinical Isolate #2

	Raw values								
	$\log_2$(MIC Piperacillin) (μg/ml)								
	Clinical Isolate 2								
	Evolved to tobramycin			Evolved to ciprofloxacin			Evolved to LB		
	1	2	3	1	2	3	1	2	3
Day									
1	5	5	5	5	5	5	8	7	7
10	7	6	7	2	4	4	8	8	8

	Normalized values								
	$\log_2$(MIC Piperacillin) (μg/ml)								
	Clinical Isolate 2								
	Evolved to tobramycin			Evolved to ciprofloxacin			Evolved to LB		
	1	2	3	1	2	3	1	2	3
Day									
1	0.00	0.00	0.00	0.00	0.00	0.00	0.67	-0.33	-0.33
10	2.00	1.00	2.00	-3.00	-1.00	-1.00	0.67	0.67	0.67

A one-way ANOVA is performed (`anova1` in MATLAB) on the normalized Day 10 $\log_2$ MIC_{PIP} values:

ANOVA Table

```
Source        SS       df      MS         F     Prob>F
-------------------------------------------------------
Columns    17.5556     2     8.77778    15.8    0.0041
Error       3.3333     6     0.55556
Total      20.8889     8
```

We also performed the non-parametric Kruskal-Wallis test (in R):

```
> kruskal.test(value ~ lineage, data = PIPRCI2)

        Kruskal-Wallis rank sum test

data:  value by lineage
Kruskal-Wallis chi-squared = 7.5789, df = 2, p-value = 0.02261
```

With an **ANOVA p-value of 0.0041** and a **Kruskal-Wallis p-value of 0.02261**, the treatments are significantly different at the alpha=0.05 level, and we continue with multiple comparisons testing with the Tukey HSD test (`multcompare` in MATLAB), and the Dunn's test (`PMCMR` package in R).

Day 10 $\log_2$ MIC_{PIP} of Clinical Isolate #2		lower bound	difference between means	upper bound	Tukey HSD p-value	Dunn's Test p-value
Evolved to tobramycin	Evolved to ciprofloxacin	1.47	3.33	5.20	0.0037	0.0059
Evolved to tobramycin	Evolved to LB	-0.87	1.00	2.87	0.3000	0.1687
**Evolved to ciprofloxacin**	**Evolved to LB**	**-4.20**	**-2.33**	**-0.47**	**0.0202**	0.1687

Clinical Isolate #3

Raw values								
**$\log_2$(MIC Piperacillin) (µg/ml)**								
Clinical Isolate 3								
Evolved to tobramycin			Evolved to ciprofloxacin			Evolved to LB		
1	2	3	1	2	3	1	2	3
7	7	7	8	7	7	9	9	9
7	8	9	5	5	5	12	11	11

Normalized values								
**$\log_2$(MIC Piperacillin) (µg/ml)**								
Clinical Isolate 3								
Evolved to tobramycin			Evolved to ciprofloxacin			Evolved to LB		
1	2	3	1	2	3	1	2	3
0.00	0.00	0.00	0.67	-0.33	-0.33	0.00	0.00	0.00
0.00	1.00	2.00	-2.33	-2.33	-2.33	3.00	2.00	2.00

A one-way ANOVA is performed (`anova1` in MATLAB) on the normalized Day 10 $\log_2$ MIC_{PIP} values:

ANOVA Table

```
Source        SS      df      MS       F     Prob>F
---------------------------------------------------
Columns    34.6667    2    17.3333    39    0.0004
Error       2.6667    6     0.4444
Total      37.3333    8
```

We also performed the non-parametric Kruskal-Wallis test (in R):

```
> kruskal.test(value ~ lineage, data = PIPRCI3)

        Kruskal-Wallis rank sum test

data:  value by lineage
Kruskal-Wallis chi-squared = 6.9524, df = 2, p-value = 0.03092
```

With an **ANOVA p-value of 0.0004** and a **Kruskal-Wallis p-value of 0.03092** and a **Kruskal-Wallis p-value of 0.03092**, the treatments are significantly different at the alpha=0.05 level, and we continue with multiple comparisons testing with the Tukey HSD test (`multcompare` in MATLAB).

Day 10 $\log_2$ MIC_{PIP} of Clinical Isolate #3		lower bound	difference between means	upper bound	Tukey HSD p-value	Dunn's Test p-value
Evolved to tobramycin	Evolved to ciprofloxacin	1.66	3.33	5.00	0.0021	0.1228
Evolved to tobramycin	Evolved to LB	-3.00	-1.33	0.34	0.1089	0.2801
**Evolved to ciprofloxacin**	**Evolved to LB**	**-6.34**	**-4.67**	**-3.00**	**0.0003**	0.0087

Hocquet Isolates (Fig 8 of main text)

A_{WT} vs. A_{PM}

Raw values

	$\log_2$(MIC Tobramycin) (µg/ml)					
	A_{WT}			A_{PM}		
	1	2	3	1	2	3
Day						
1	0	2	3	1	1	1
15	6	6	5	3	2	3

Normalized values

$\log_2$(MIC Tobramycin) (µg/ml)					
A_{WT}			A_{PM}		
1	2	3	1	2	3
-1.67	0.33	1.33	0.00	0.00	0.00
4.33	4.33	3.33	2.00	1.00	2.00

The normalized values are calculated by subtracting the average of the Day 1 $\log_2$ MIC_{TOB} values from the raw values. More explicitly,

Normalized $\log_2$ MIC_{TOB} of Day 1 A_{WT}:
`[0 2 3] - mean([0 2 3]) = [-1.67 0.33 1.33]`

Normalized $\log_2$ MIC_{TOB} of Day 1 A_{PM}:
`[1 1 1] - mean([1 1 1]) = [0 0 0]`

Normalized $\log_2$ MIC_{TOB} of Day 15 A_{WT}:
`[6 6 5] - mean([0 2 3]) = [4.33 4.33 3.33]`

Normalized $\log_2$ MIC_{TOB} of Day 15 A_{PM}:
`[3 2 3] - mean([1 1 1]) = [2 1 2]`

A two-sample t-test is (`ttest2` in MATLAB) then performed on the normalized $\log_2$ MIC_{TOB} values of Day 15 A_{WT} vs. Day 15 A_{PM} and yields **p= 0.0078.**

A Wilcoxon rank sum test (`ranksum` in MATLAB) was also performed on the normalized $\log_2$ MIC_{TOB} values of Day 15 A_{WT} vs. Day 15 A_{PM} and yielded **p= 0.1.** Note that the smallest p-value that can be detected with a two-sided Wilcoxon rank sum test with three samples in both groups is 0.1.

Similar calculations are done for the B, C, and D pairs of clinical isolates.

B_{WT} vs. B_{PM}

Raw values

	log₂(MIC Tobramycin) (µg/ml)					
	B_{WT}			B_{PM}		
	1	2	3	1	2	3
Day						
1	1	2	1	-2		-2
15	6	6	7	-1		1

Normalized values

	$\log_2$(MIC Tobramycin) (µg/ml)					
	B_{WT}			B_{PM}		
	1	2	3	1	2	3
Day						
1	-0.33	0.67	-0.33	0.00		0.00
15	4.67	4.67	5.67	1.00		3.00

Two-sample t-test of normalized Day 15 B_{WT} vs. Day 15 B_{PM} yields: **p = 0.040**

A Wilcoxon rank sum test (`ranksum` in MATLAB) was also performed on the normalized $\log_2$ MIC_{TOB} values of Day 15 B_{WT} vs. Day 15 B_{PM} and yielded **p= 0.1.** Note that the smallest p-value that can be detected with a two-sided Wilcoxon rank sum test with three samples in both groups is 0.1.

C_{WT} vs. C_{PM}

Raw values

	log₂(MIC Tobramycin) (µg/ml)					
	C_{WT}			C_{PM}		
	1	2	3	1	2	3
Day						
1	1	2	2	1	1	1
15	5	6	6	2	3	4

Normalized values

	log₂(MIC Tobramycin) (µg/ml)					
	C_{WT}			C_{PM}		
	1	2	3	1	2	3
Day						
1	-0.67	0.33	0.33	0.00	0.00	0.00
15	3.33	4.33	4.33	1.00	2.00	3.00

Two-sample t-test of normalized Day 15 C_{WT} vs. Day 15 C_{PM} yields: **p = 0.040**

A Wilcoxon rank sum test (`ranksum` in MATLAB) was also performed on the normalized $\log_2$ MIC_{TOB} values of Day 15 C_{WT} vs. Day 15 C_{PM} and yielded **p= 0.1.** Note that the smallest p-value that can be detected with a two-sided Wilcoxon rank sum test with three samples in both groups is 0.1.

D_{WT} vs. D_{PM}

	Raw values						Normalized values					
	**log_2(MIC Tobramycin) (µg/ml)**						**log_2(MIC Tobramycin) (µg/ml)**					
	D_{WT}			D_{PM}			D_{WT}			D_{PM}		
	1	2	3	1	2	3	1	2	3	1	2	3
Day												
1	1	1	2	1	1	1	-0.33	-0.33	0.67	0.00	0.00	0.00
15	4	4	4	4	3	4	2.67	2.67	2.67	3.00	2.00	3.00

Two-sample t-test of normalized Day 15 D_{WT} vs. Day 15 D_{PM} yields: **p = 1**

A Wilcoxon rank sum test (`ranksum` in MATLAB) was also performed on the normalized log_2 MIC_{TOB} values of Day 15 D_{WT} vs. Day 15 D_{PM} and yielded **p= 0.4.**
