# Supplementary figures and images for: History of antibiotic adaptation influences microbial evolutionary dynamics during subsequent treatment
Source: PLoS Biol. 2017 Aug 8;15(8):e2001586. doi: 10.1371/journal.pbio.2001586 (PMC5549691; doi:10.1371/journal.pbio.2001586)

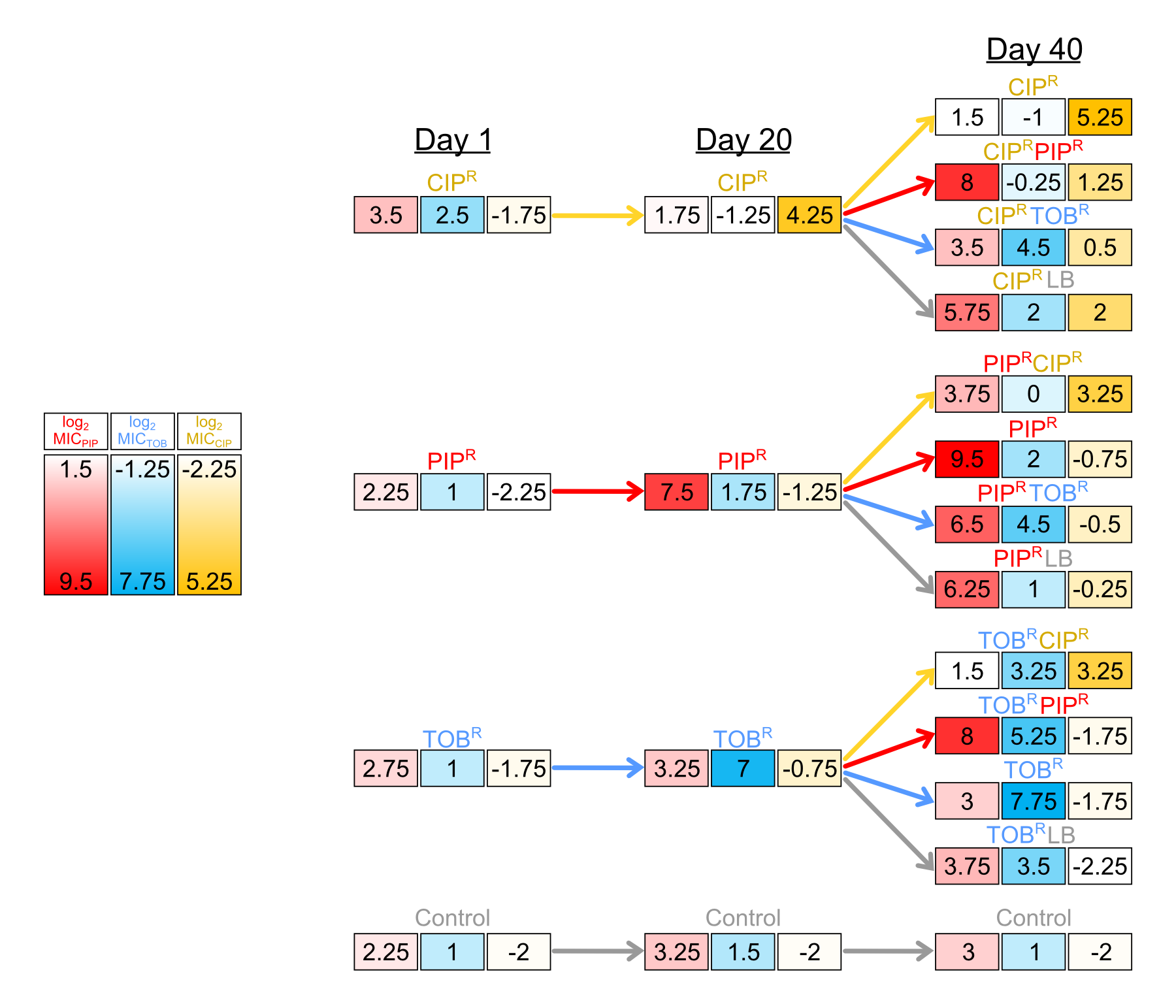

Supplement: S2 Fig — This figure summarizes the data presented in Fig 2 of the main text. The Day 1, Day 20, and Day 40 log2 MIC values (μg/ml) of piperacillin (PIP), tobramycin (TOB), and ciprofloxacin (CIP) are shown for all the evolved lineages of the main adaptive evolution experiment. The values are the average of 4 replicates per lineage (S3 Data). For each lineage, the left, middle, and right boxes denote the MICPIP, MICTOB, and MICCIP, respectively. The color intensity is normalized by the minimum and maximum MIC of each drug across all the lineages. For example, for log2 MICPIP, the lowest value is 1.5, which is seen in Day 40 CIPR, and the highest log2 MICPIP is 9.5, which is seen in Day 40 PIPR. The color of the arrow denotes the treatment. (TIF) [file pbio.2001586.s004.tif]

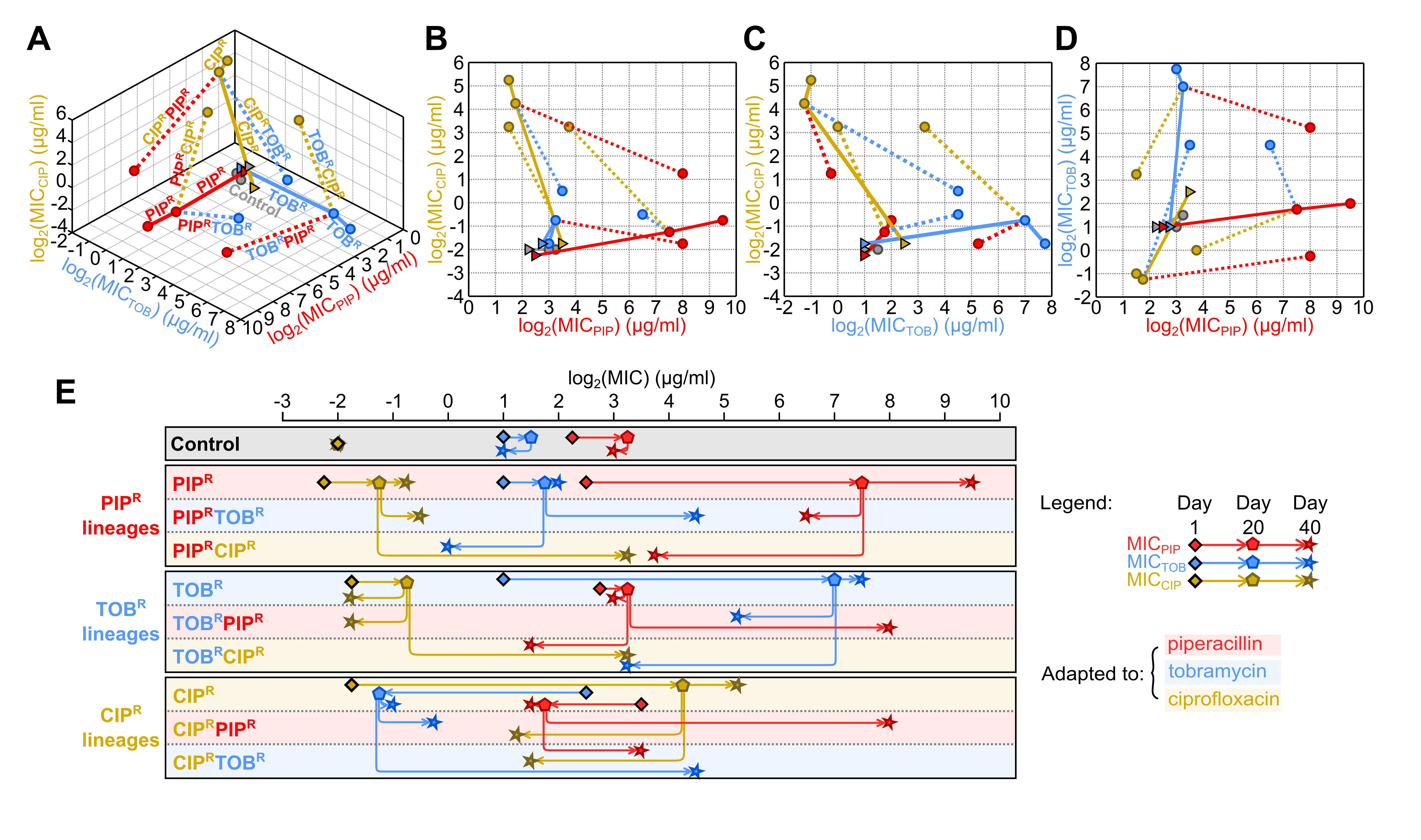

Supplement: S3 Fig — All values shown are the averages of 4 replicates (S3 Data). (A) The MICs of the 3 drugs for Days 1, 20, and 40 for all treatments are plotted in 3D MIC space to show how the MIC profiles change over the course of adaptation. Day 1 MICs are denoted by the triangles. A “nonright angle” indicates a change in resistance to 1 (or more) of the other drug(s). The color/style of the line indicates the treatment and is labeled as such. (B to D) 2D projections of (A). Labels for the lines carry over from (A). (E) Changes in average MICs for all drugs for all treatments are plotted on a single axis to better facilitate quantitative comparison. Here, red, blue, and yellow lines denote MICs to piperacillin (PIP), tobramycin (TOB), and ciprofloxacin (CIP), respectively. (TIF) [file pbio.2001586.s005.tif]

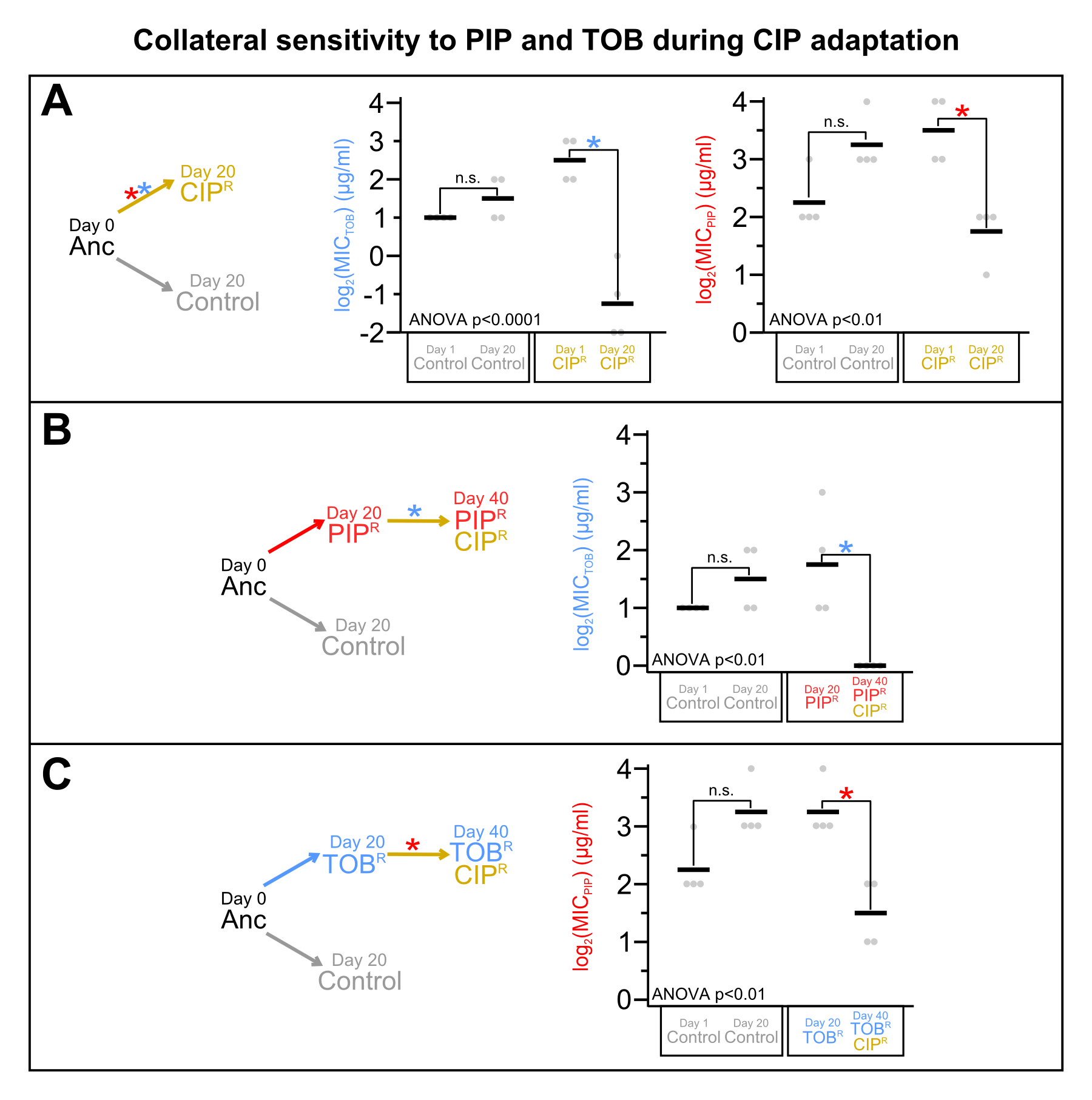

Supplement: S4 Fig — (A) Collateral sensitivities to tobramycin (left) and piperacillin (right) were observed during the evolution starting from Day 0 Ancestor to ciprofloxacin. While there were no statistically significant changes in MICTOB and MICPIP after 20 days of evolution to LB in the Control, there were significant decreases after 20 days of evolution to ciprofloxacin. Similarly, (B) there was a significant decrease in MICTOB when Day 20 PIPR was subsequently adapted to ciprofloxacin, (C) and in MICPIP when Day 20 TOBR was subsequently adapted to ciprofloxacin. For all 3 panels, the asterisks denote p < 0.05 (Tukey’s HSD test), n.s. denotes p > 0.05, and the color of the asterisks denotes which drug minimum inhibitory concentration (MIC) is being compared. In the plots, for each lineage being shown, the black bar denotes the mean of the 4 individual replicate values (gray dots). See S2 Text for the calculations of the statistical tests and S3 Data for the raw MIC data. (TIF) [file pbio.2001586.s006.tif]

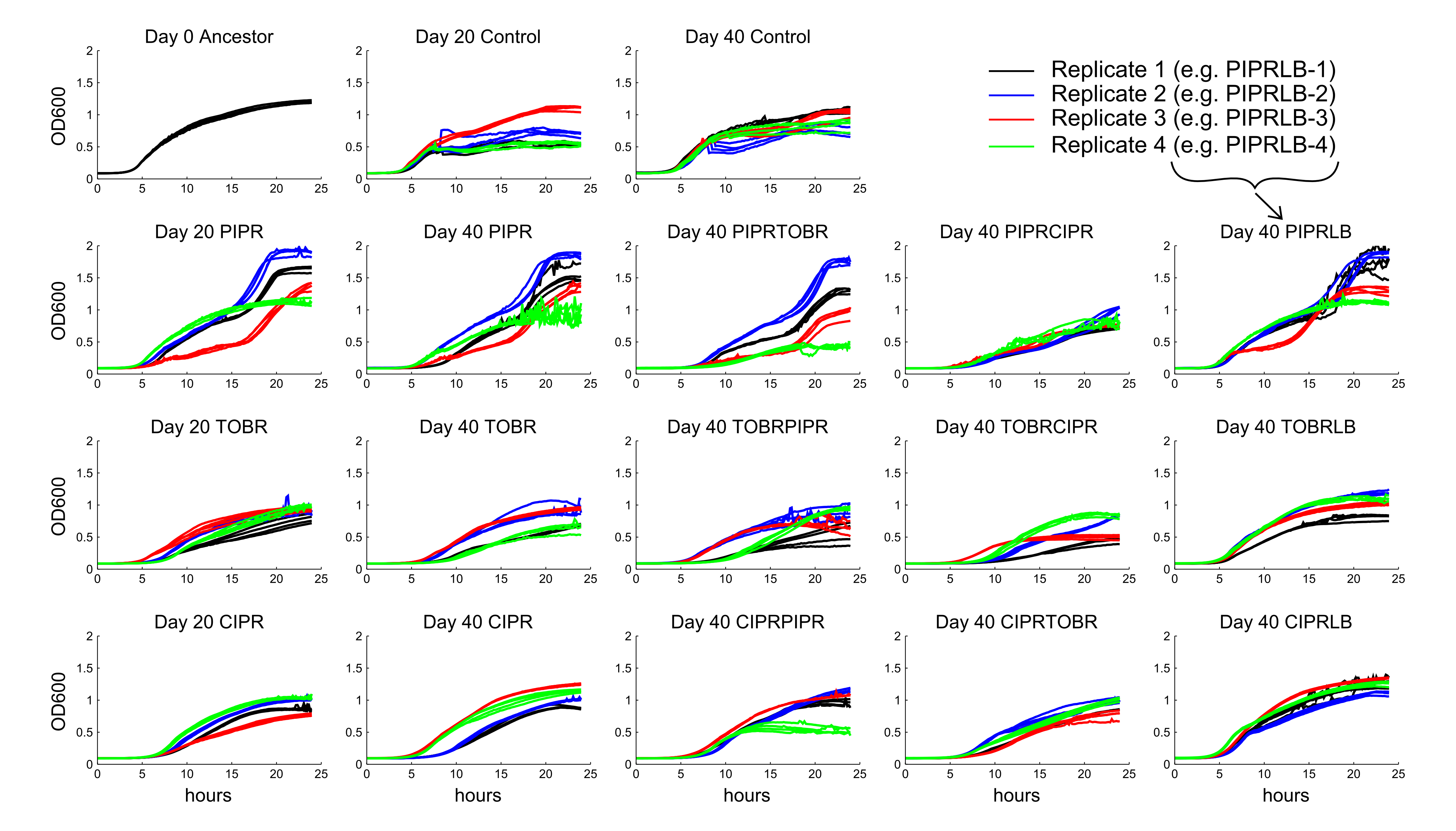

Supplement: S5 Fig — The OD600 was measured over the course of 24 hours for the 68 evolved replicate lineages (17 lineages of 4 biological replicates each) as well as the Day 0 Ancestor in quadruplicates grown in lysogeny broth (LB). Note that because of the pyomelanin hyperproduction, replicates 1, 2, and 3 of the PIPR and PIPR-derived lineages reach higher final OD600’s than the replicate 4 lineages as is apparent in the curves above (as discussed in the main text). (TIF) [file pbio.2001586.s007.tif]

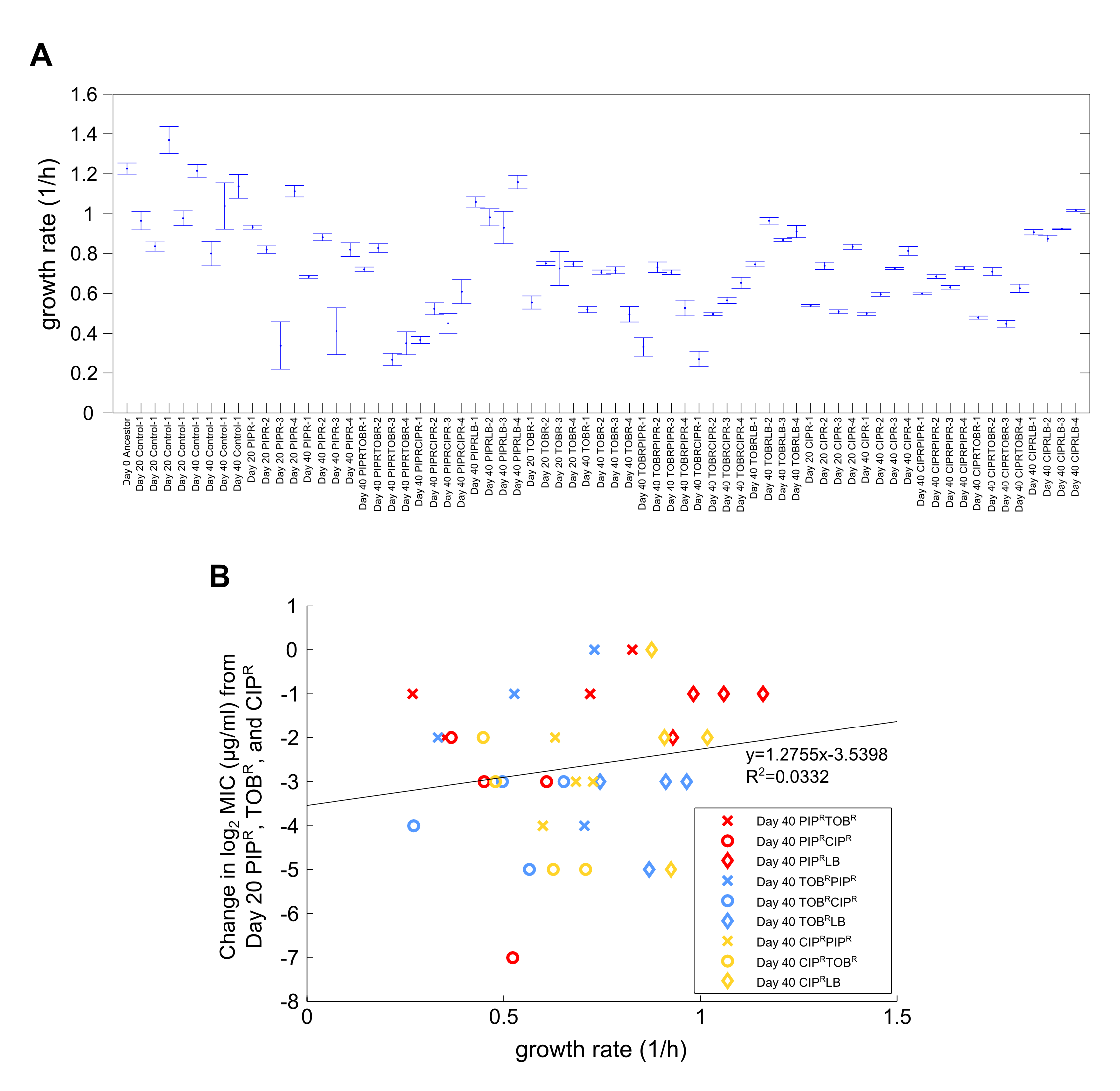

Supplement: S6 Fig — (A) The growth rates were calculated from the growth curves presented in S5 Fig. The means and standard deviations of the 4 replicates for each of the replicate lineages are shown. (B) The correlation between the growth rates of the Day 40 lineages (x-axis) and the change in minimum inhibitory concentration (MIC) of the corresponding Day 20 lineages (y-axis) was calculated. The data suggest no correlation between growth rate and the degree to which the MIC changes from Day 20 to Day 40. For example, the 4 red crosses show the growth rates of Day 40 PIPRTOBR-1, -2, -3, and -4 on the x-axis plotted against the log2 MICPIP of Day 20 PIPR-1, -2, -3, and -4 minus the log2 MICPIP of Day 40 PIPRTOBR-1, -2, -3, and -4, respectively on the y-axis. See S4 Data for the growth rate data. (TIF) [file pbio.2001586.s008.tif]

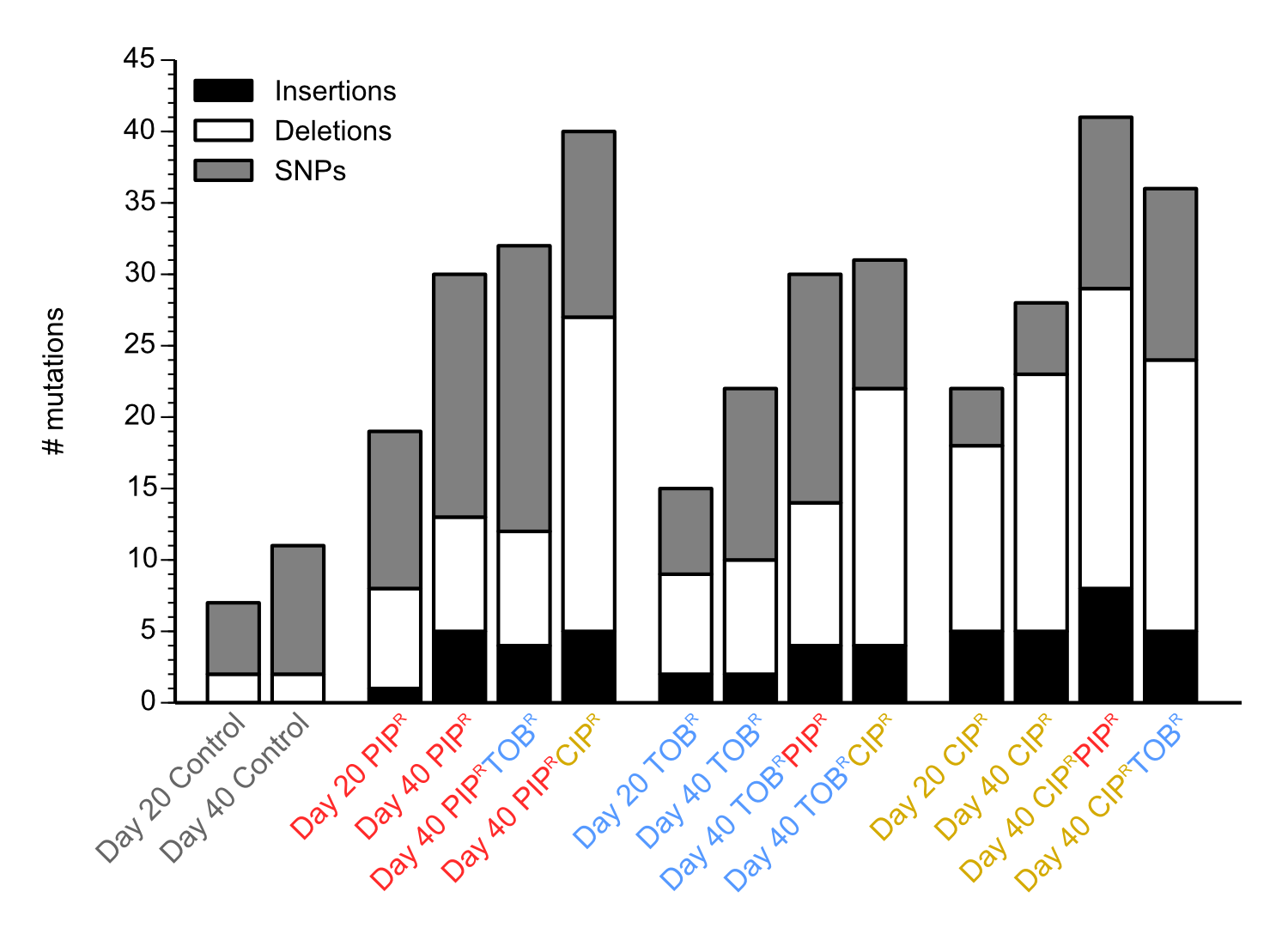

Supplement: S7 Fig — Histogram of the number of mutations shows that overall, lineages that were evolved to ciprofloxacin accumulated the most mutations and had comparably more deletion mutations. See S1 Table for the complete list of mutations. (TIF) [file pbio.2001586.s009.tif]

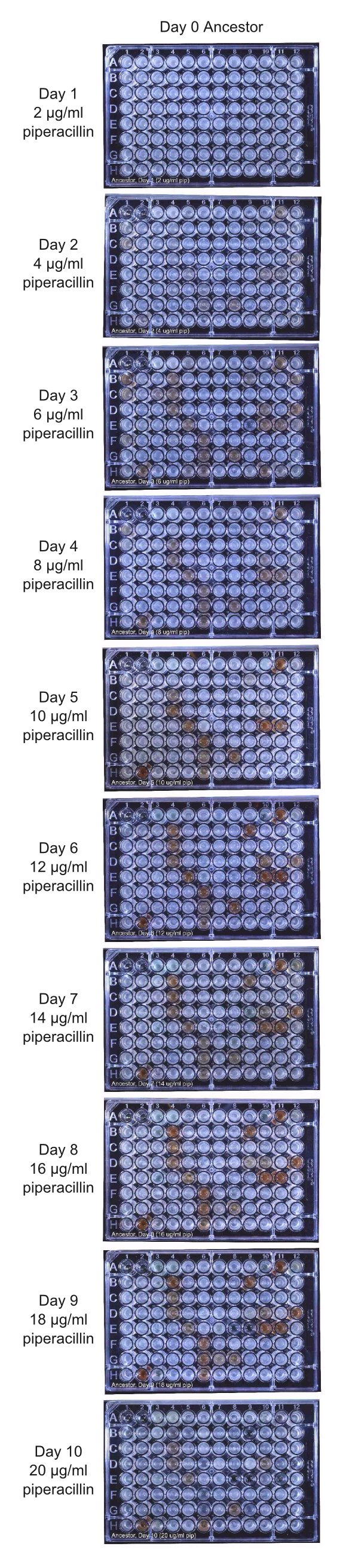

Supplement: S8 Fig — Ninety-two replicates of Day 0 Ancestor were serially passaged with a replicator tool for 10 days to increasing concentrations of piperacillin. (TIF) [file pbio.2001586.s010.tif]

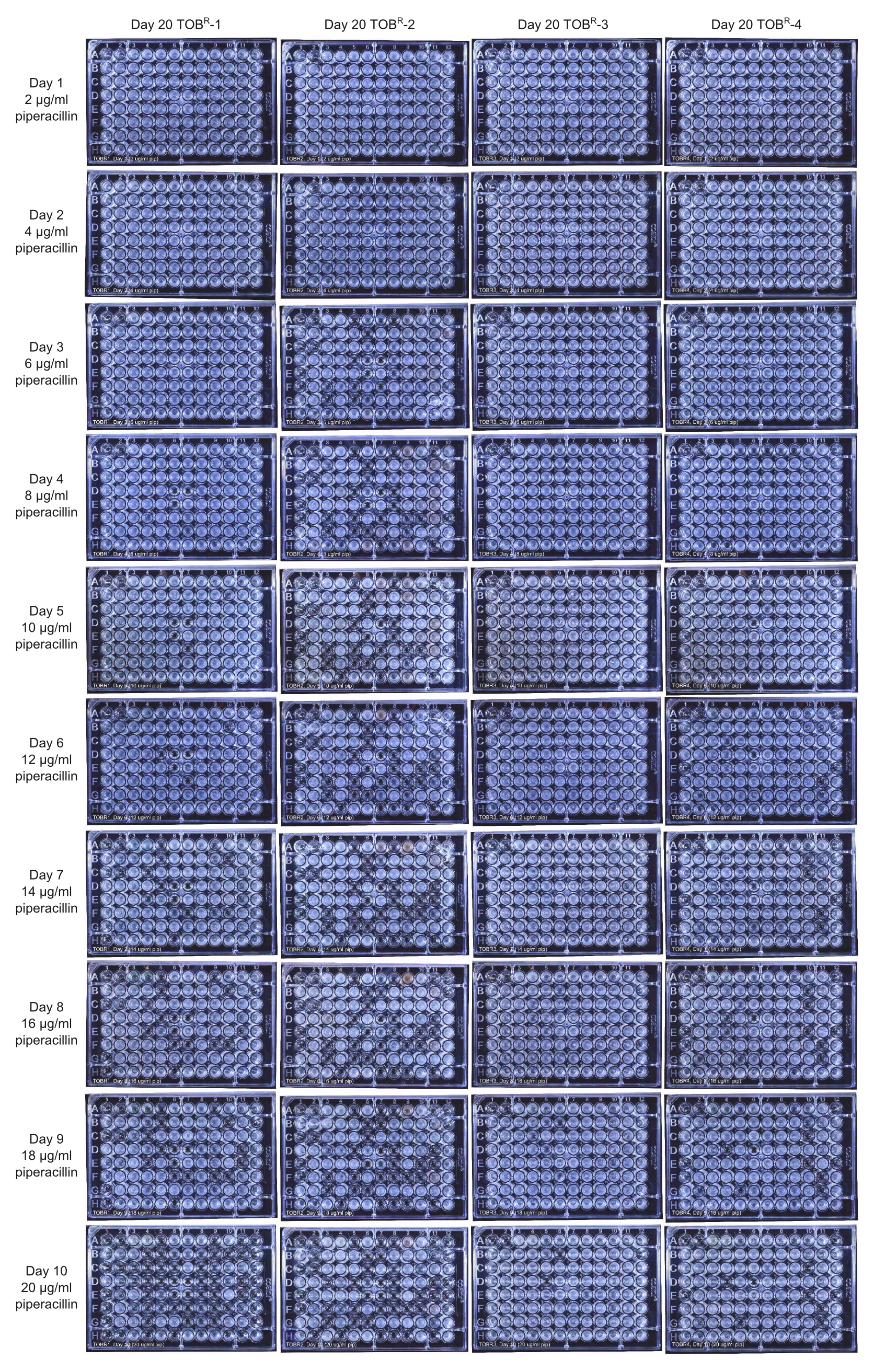

Supplement: S9 Fig — Ninety-two replicates of Day 20 TOBR-1, -2, -3, and -4 were serially passaged with a replicator tool for 10 days to increasing concentrations of piperacillin. (TIF) [file pbio.2001586.s011.tif]

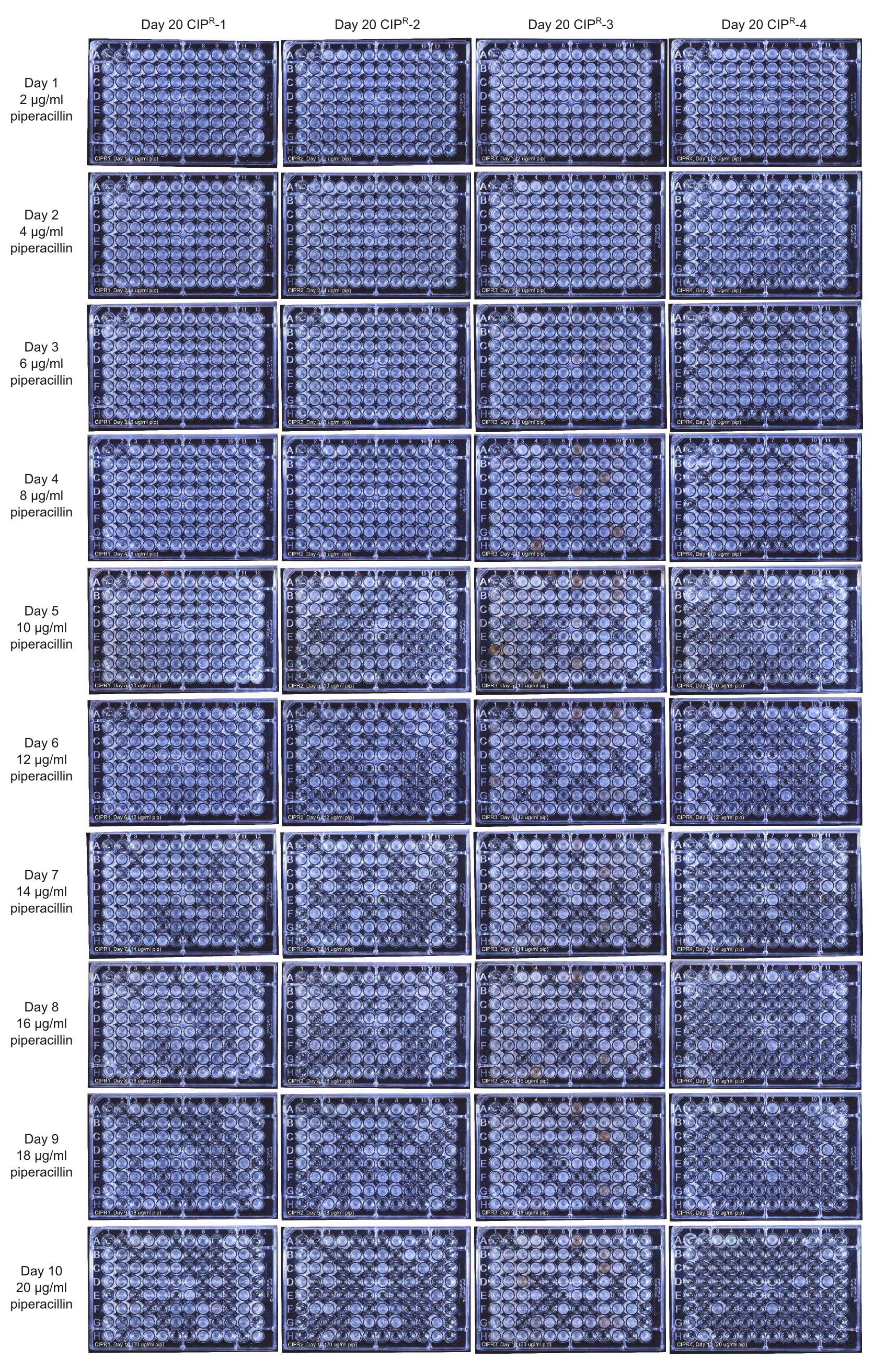

Supplement: S10 Fig — Ninety-two replicates of Day 20 CIPR-1, -2, -3, and -4 were serially passaged with a replicator tool for 10 days to increasing concentrations of piperacillin. (TIF) [file pbio.2001586.s012.tif]

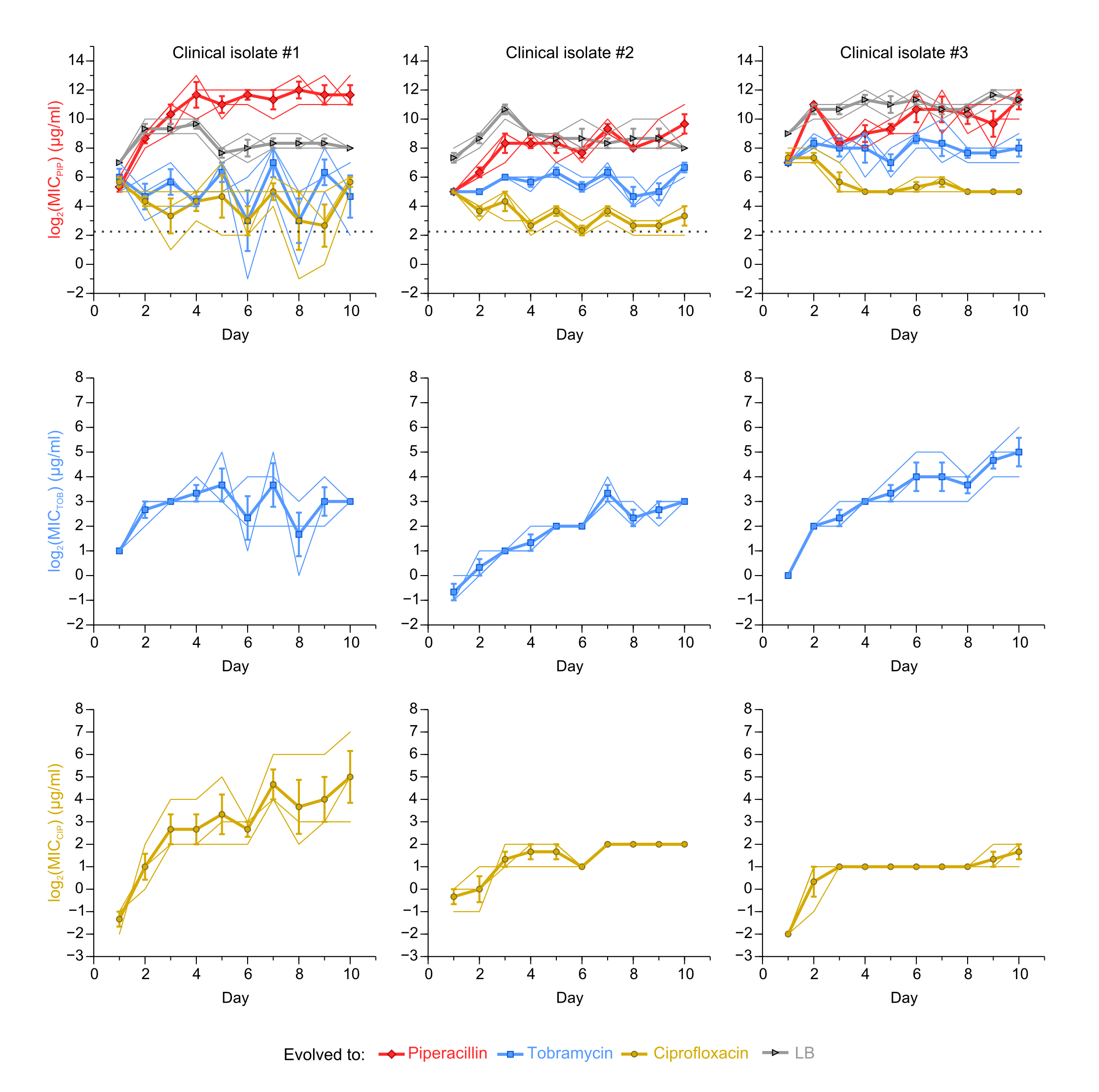

Supplement: S11 Fig — Three clinical isolates of P. aeruginosa with high piperacillin resistance were evolved to piperacillin, tobramycin, and ciprofloxacin to test if we could recapitulate the evolutionary dynamics seen in MICPIP of PIPR, whereby evolution to ciprofloxacin would cause MICPIP to decrease, while evolution to tobramycin would not. We were able to see this result recapitulated in isolate #2 and isolate #3, but not in isolate #1 (see main text). Interestingly, isolate #1 was able to be evolved to higher levels of piperacillin resistance and ciprofloxacin resistance compared to the other 2. Thin lines show the individual time courses of 3 replicates per treatment, and bold lines show their averages. The dotted line in the first row shows the mean MICPIP of Day 1 Control to emphasize that the clinical isolates are resistant to piperacillin at Day 1. Error bars show SEM for the 3 replicates for each lineage. See S3 Data for the raw numerical data. (TIF) [file pbio.2001586.s013.tif]

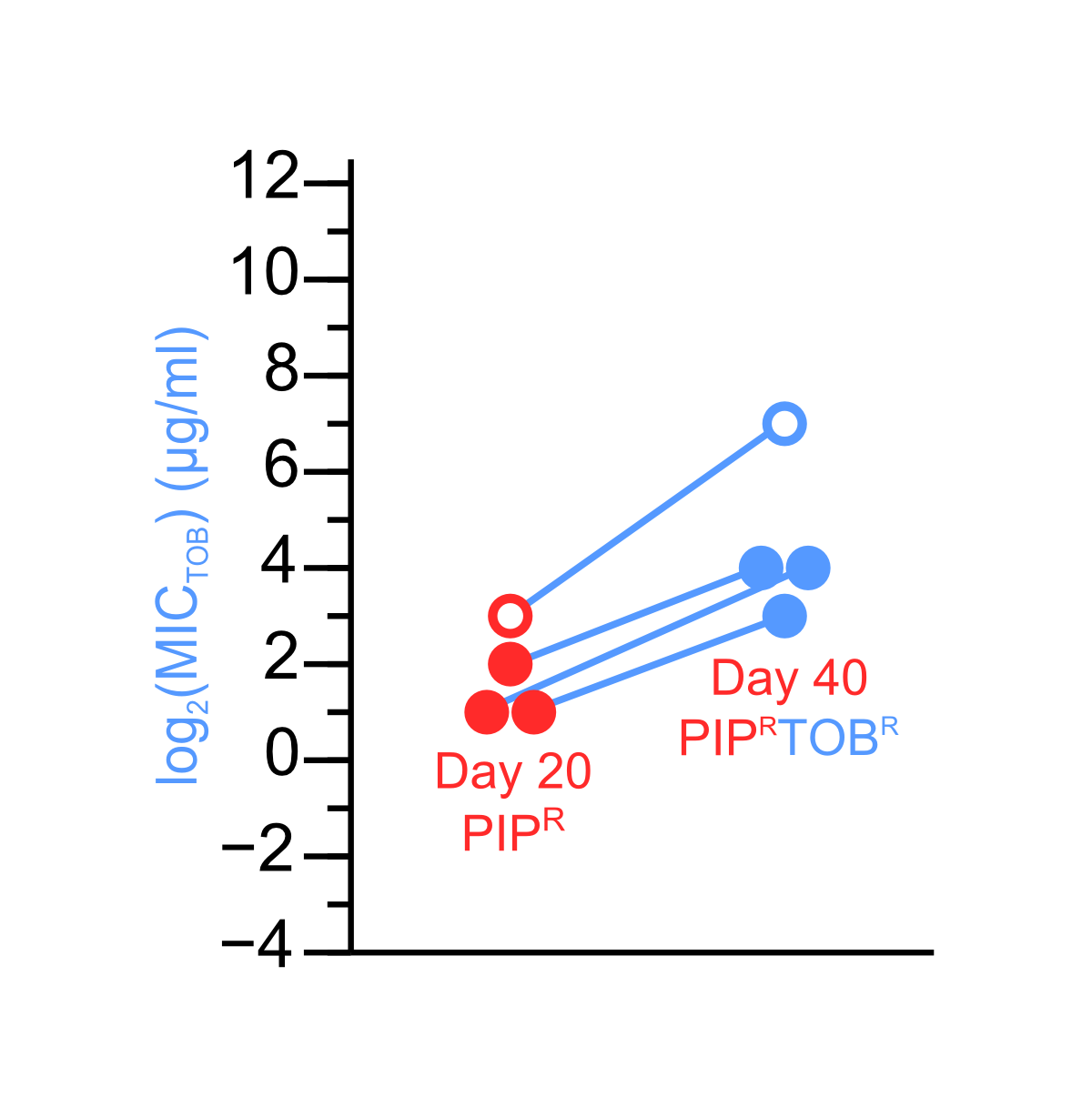

Supplement: S12 Fig — The resistance levels to tobramycin for individual replicates are plotted for Day 20 PIPR and Day 40 PIPRTOBR. The replicates denoted with the filled-in circles have large deletions in their genome, while the replicate denoted by the open circle does not. We see that the replicates of Day 20 PIPR with the large chromosomal deletions develop less resistance to tobramycin than the replicate that does not have the deletion. See S3 Data for the raw numerical data. (TIF) [file pbio.2001586.s014.tif]

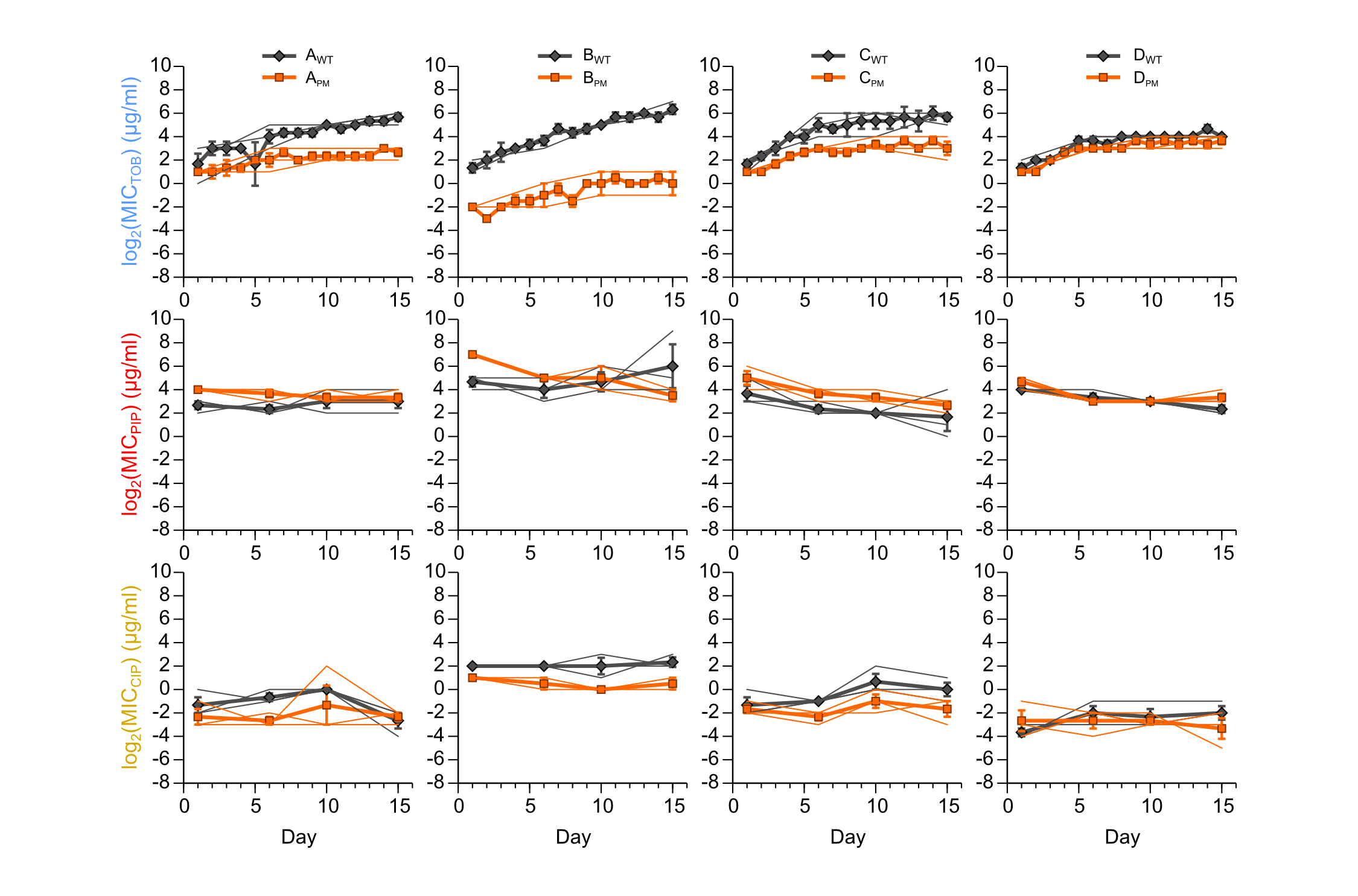

Supplement: S13 Fig — Four pairs of clinical isolates of P. aeruginosa were evolved to tobramycin. Each pair of isolates (columns) consists of a pyomelanogenic isolate (PM) that has a large deletion, and a parental isolate from which the PM isolate is derived (wild-type [WT]). In each pair, the only genetic difference is the presence of a large chromosomal deletion in the PM isolate. The top, middle, and bottom rows show the minimum inhibitory concentrations (MICs) of the isolates to tobramycin, piperacillin, and ciprofloxacin, respectively, as they adapt to tobramycin. Thin lines show the individual time courses of 3 replicates per treatment (with the exception of BPM, which has 2 replicates), and bold lines show their averages. Error bars show SEM for the 3 replicates (2 for BPM) for each lineage. See S3 Data for the raw numerical data. (TIF) [file pbio.2001586.s015.tif]
