# Supplementary material for: History of antibiotic adaptation influences microbial evolutionary dynamics during subsequent treatment
Source: PLoS Biol. 2017 Aug 8;15(8):e2001586. doi: 10.1371/journal.pbio.2001586 (PMC5549691; doi:10.1371/journal.pbio.2001586)
Supplement: S2 Table — (DOCX) [file pbio.2001586.s022.docx]

Supplementary Materials (S2 Table) for

History of Antibiotic Adaptation Influences Microbial Evolutionary Dynamics During Subsequent Treatment

Phillip Yen and Jason Papin*

*Corresponding author. E-mail: papin@virginia.edu

| Gene | Locus tag | Functional class | Description |
| --- | --- | --- | --- |
| [*aldG*]–[*acsA*] | [PA14_33890]-[PA14_38690] | Large deletions | 380 genes |
| [*ccoP*]-[*ccoP*] | [PA14_44360]-[PA14_44400] | Energy | 5 genes |
| [*flgJ*]–[*flgI*] | [PA14_50380]-[PA14_50410] | Flagella | [*flgJ*], [*flgI*] |
| [*glgX*]–[*nhaB*] | [PA14_36630]-[PA14_41000] | Large deletions | 341 genes |
| [*nfxB*] | [PA14_60860] | MexCD-OprJ | [*nfxB*] |
| [PA14_12210] | [PA14_12210] | Membrane | [PA14_12210] |
| [PA14_37690]–[PA14_39660] | [PA14_37690]–[PA14_39660] | Large deletions | 151 genes |
| *aceA* | PA14_66290 | Metabolism | pyruvate dehydrogenase, E1 component |
| *algC* | PA14_70270 | Membrane | phosphomannomutase AlgC |
| *ampR* | PA14_10800 | Beta-lactamases | transcriptional regulator AmpR |
| *amrB* | PA14_38410 | MexXY-OprM | RND multidrug efflux transporter |
| *aotJ* | PA14_52790 | Membrane | arginine/ornithine binding protein AotJ |
| *aprX*/PA14_48150 | PA14_48140/PA14_48150 | Hypothetical | conserved hypothetical protein/hypothetical protein |
| *aroB* | PA14_66600 | Metabolism | 3-dehydroquinate synthase |
| *atpC* | PA14_73230 | Energy | ATP synthase epsilon chain |
| *atpC/atpD* | PA14_73230/PA14_73240 | Energy | ATP synthase epsilon chain/ATP synthase beta chain |
| *cheB* | PA14_45580 | Flagella | putative chemotaxis methylesterase |
| *chpA* | PA14_05390 | Chemotaxis | ChpA |
| *clpA* | PA14_30230 | Metabolism | ATP-dependent clp protease, ATP-binding subunit ClpA |
| *clpS* | PA14_30210 | Metabolism | ATP-dependent Clp protease adaptor protein clpS |
| *cpxR* | PA14_22760 | Two-component sensor | putative transcriptional regulator in 2-component system |
| *cycB/pauR* | PA14_69970/PA14_69980 | Energy | cytochrome c5/putative transcriptional regulator |
| *dacB* | PA14_24690 | Beta-lactamases | putative D-alanyl-D-alanine carboxypeptidase |
| *dacC* | PA14_12100 | Cell wall | D-ala-D-ala-carboxypeptidase |
| *dadA* | PA14_70040 | Metabolism | D-amino acid dehydrogenase, small subunit |
| *envZ* | PA14_68680 | Two-component sensor | two-component sensor EnvZ |
| *erfK* | PA14_27180 | Hypothetical | putative ErfK/YbiS/YcfS/YnhG family protein |
| *fixI* | PA14_44440 | Membrane | putative cation-transporting P-type ATPase |
| *fleN* | PA14_45640 | Flagella | flagellar synthesis regulator FleN |
| *flgF* | PA14_50440 | Flagella | flagellar basal-body rod protein FlgF |
| *flgG* | PA14_50430 | Flagella | flagellar basal-body rod protein FlgG |
| *flgK* | PA14_50360 | Flagella | flagellar hook-associated protein 1 FlgK |
| *fliA* | PA14_45630 | Flagella | motility sigma factor FliA |
| *fliP* | PA14_45770 | Flagella | flagellar biosynthetic protein FliP |
| *fusA1* | PA14_08820 | Ribosome | elongation factor G |
| *gcdH* | PA14_05840 | Metabolism | glutaryl-CoA dehydrogenase |
| *gcvP2* | PA14_33000 | Metabolism | glycine cleavage system protein P2 |
| *gltA* | PA14_44070 | Metabolism | citrate synthase |
| *gyrA* | PA14_23260 | DNA/RNA synthesis | DNA gyrase subunit A |
| *gyrB* | PA14_00050 | DNA/RNA synthesis | DNA gyrase subunit B |
| *intT*–PA14_49030 | PA14_48880–PA14_49030 | Large deletions | 16 genes |
| *iscR* | PA14_14710 | Transcriptional regulation | putative Rrf2 family protein |
| *lhpE* | PA14_47860 | Metabolism | putative oxidoreductase |
| *mexA* | PA14_05530 | MexAB-OprM | RND multidrug efflux membrane fusion protein MexA precursor |
| *mexC* | PA14_60850 | MexCD-OprJ | multidrug efflux RND membrane fusion protein |
| *mexC/nfxB* | PA14_60850/PA14_60860 | MexCD-OprJ | multidrug efflux RND membrane fusion protein/transcriptional regulatory protein NfxB |
| *mexD* | PA14_60830 | MexCD-OprJ | multidrug efflux RND transporter MexD |
| *mexF* | PA14_32390 | MexEF-OprN | RND multidrug efflux transporter MexF |
| *mexR* | PA14_05520 | MexAB-OprM | multidrug resistance operon repressor MexR |
| *mexR/mexA* | PA14_05520/PA14_05530 | MexAB-OprM | multidrug resistance operon repressor MexR/RND multidrug efflux membrane fusion protein MexA precursor |
| *mexS* | PA14_32420 | MexEF-OprN | putative Zn-dependent oxidoreductase |
| *mexT* | PA14_32410 | MexEF-OprN | transcriptional regulator MexT |
| *miaA* | PA14_65320 | Ribosome | delta 2-isopentenylpyrophosphate transferase |
| *minC* | PA14_22040 | Cell division | cell division inhibitor MinC |
| *morA* | PA14_60870 | Flagella | motility regulator |
| *mpl* | PA14_11845 | Cell wall | UDP-N-acetylmuramate:L-alanyl-gamma-D-glutamyl- meso-diaminopimelate ligase |
| *mucB* | PA14_54410 | Transcriptional regulation | negative regulator for alginate biosynthesis MucB |
| *muxA* | PA14_31870 | MuxABC | putative RND efflux membrane fusion protein precursor |
| *mvfR* | PA14_51340 | Transcriptional regulation | Transcriptional regulator MvfR |
| *nalC* | PA14_16280 | MexAB-OprM | putative transcriptional regulator |
| *nalC*/PA14_16290 | PA14_16280/PA14_16290 | MexAB-OprM | putative transcriptional regulator/conserved hypothetical protein |
| *nalD* | PA14_18080 | MexAB-OprM | putative transcriptional regulator, TetR family |
| *nfxB* | PA14_60860 | MexCD-OprJ | transcriptional regulatory protein NfxB |
| *np20* | PA14_72560 | Transcriptional regulation | transcriptional regulator np20 |
| *nppA1* | PA14_41110 | Membrane | putative solute-binding protein |
| *nuoB* | PA14_30010 | NADH dehydrogenase | NADH dehydrogenase I chain B |
| *nuoG* | PA14_29940 | NADH dehydrogenase | NADH dehydrogenase I chain G |
| *nuoL* | PA14_29880 | NADH dehydrogenase | NADH dehydrogenase I chain L |
| *nuoM* | PA14_29860 | NADH dehydrogenase | NADH dehydrogenase I chain M |
| *orfH* | PA14_23380 | Flagella | UDP-N-acetyl-D-mannosaminuronate dehydrogenase |
| *orfJ* | PA14_23410 | Flagella | putative glycosyl transferase |
| *orfN* | PA14_23460 | Flagella | putative group 4 glycosyl transferase |
| PA14_09960 | PA14_09960 | Transcriptional regulation | putative transcriptional regulator |
| PA14_12140 | PA14_12140 | Transcriptional regulation | putative transcriptional regulator |
| PA14_20960 | PA14_20960 | Metabolism | putative isomerase |
| PA14_21820 | PA14_21820 | Metabolism | putative peptidyl-prolyl cis-trans isomerase, FkbP-type |
| PA14_22730 | PA14_22730 | Two-component sensor | putative two component sensor histidine kinase protein |
| PA14_25490 | PA14_25490 | Membrane | putative tolQ-type transport protein |
| PA14_27360/*deaD* | PA14_27360/PA14_27370 | Metabolism | putative enoyl-CoA hydratase/putative ATP-dependent RNA helicase, DEAD box family |
| PA14_27940 | PA14_27940 | Two-component sensor | putative two-component response regulator |
| PA14_30540/*ssuA* | PA14_30540/PA14_30550 | Membrane | putative periplasmic aliphatic sulfonate-binding protein/putative periplasmic aliphatic sulfonate-binding protein |
| PA14_31100/PA14_31110 | PA14_31100/PA14_31110 | DNA | putative plasmid partitioning protein/putative replication initiator and transcriptional repressor protein |
| PA14_34500 | PA14_34500 | Membrane | putative ATP-binding component of ABC transporter |
| PA14_35210 | PA14_35210 | Transcriptional regulation | putative transcriptional regulator, TetR family |
| PA14_35720–[PA14_40040] | PA14_35720–[PA14_40040] | Large deletions | 343 genes |
| PA14_37170/*ada* | PA14_37170/PA14_37190 | Transcriptional regulation | conserved hypothetical protein/O6-methylguanine-DNA methyltransferase |
| PA14_38500 | PA14_38500 | Transcriptional regulation | putative transcriptional regulator, IclR family |
| PA14_39360 | PA14_39360 | Transcriptional regulation | putative sigma-54 dependent transcriptional regulator |
| PA14_41710 | PA14_41710 | Membrane | putative membrane protein |
| PA14_41730 | PA14_41730 | Hypothetical | conserved hypothetical protein |
| PA14_44990 | PA14_44990 | Hypothetical | conserved hypothetical protein |
| PA14_48800 | PA14_48800 | Membrane | putative lipoprotein |
| PA14_49300 | PA14_49300 | Metabolism | probable lipoxygenase |
| PA14_51910 | PA14_51910 | Hypothetical | hypothetical protein |
| PA14_57470 | PA14_57470 | Metabolism | putative methyltransferases |
| PA14_57540 | PA14_57540 | Energy | putative cytochrome c1 precursor |
| PA14_57570 | PA14_57570 | Energy | putative cytochrome c reductase, iron-sulfur subunit |
| PA14_57850 | PA14_57850 | Hypothetical | conserved hypothetical protein |
| PA14_57880 | PA14_57880 | Membrane | putative toluene tolerance ABC efflux transporter |
| PA14_65570 | PA14_65570 | Hypothetical | conserved hypothetical protein |
| PA14_66170 | PA14_66170 | Metabolism | putative carbamoyltransferase |
| PA14_69250 | PA14_69250 | Hypothetical | putative membrane-associated protein |
| *parS* | PA14_41270 | MexEF-OprN | putative two-component sensor |
| *pauR* | PA14_69980 | Transcriptional regulation | putative transcriptional regulator |
| *pckA* | PA14_68580 | Energy | phosphoenolpyruvate carboxykinase |
| *pepA* | PA14_14470 | Metabolism | leucine aminopeptidase |
| *pmrB* | PA14_63160 | Two-component sensor | two-component sensor |
| *prs* | PA14_61770 | Metabolism | ribose-phosphate pyrophosphokinase |
| *ptsP* | PA14_04410 | Quorum sensing | phosphoenolpyruvate-protein phosphotransferase |
| *rne* | PA14_25560 | DNA/RNA synthesis | ribonuclease E |
| *rne/rluC* | PA14_25560/PA14_25580 | Ribosome | ribonuclease E/ribosomal large subunit pseudouridine synthase C |
| *rnk* | PA14_69630 | Transcriptional regulation | nucleoside diphosphate kinase regulator |
| *rplF* | PA14_09000 | Ribosome | 50S ribosomal protein L6 |
| *rplJ* | PA14_08740 | Ribosome | 50S ribosomal protein L10 |
| *rplL* | PA14_08750 | Ribosome | 50S ribosomal protein L7 / L12 |
| *rpoC* | PA14_08780 | DNA/RNA synthesis | DNA-directed RNA polymerase beta* chain |
| *rpoN* | PA14_57940 | DNA/RNA synthesis | RNA polymerase sigma-54 factor |
| *rpsL* | PA14_08790 | Ribosome | 30S ribosomal protein S12 |
| *sahH* | PA14_05620 | Metabolism | S-adenosyl-L-homocysteine hydrolase |
| *secA* | PA14_57220 | Membrane | preprotein translocase, SecA subunit |
| *spoT* | PA14_70470 | Stringent response | guanosine-3',5'-bis(diphosphate) 3'-pyrophosphohydrolase |
| *sucD* | PA14_43940 | Energy | succinyl-CoA synthetase alpha chain |
| *topA* | PA14_25110 | DNA/RNA synthesis | DNA topoisomerase I |
| tRNA-Thr/tufB | PA14_08670/PA14_08680 | Ribosome | tRNA-Thr/elongation factor Tu |
| tRNA-Val | PA14_28190 | DNA/RNA synthesis | tRNA-Val |
| *ttg2D* | PA14_57840 | Hypothetical | putative toluene tolerance protein |
| *wbpM* | PA14_23470 | Membrane | nucleotide sugar epimerase/dehydratase WbpM |
| *wspA* | PA14_16430 | Flagella | putative methyl-accepting chemotaxis transducer |
| *ycjJ* | PA14_17740 | Membrane | putative amino acid/amine transport protein |
| *zipA* | PA14_44670 | Cell division | cell division protein ZipA |

Brackets (e.g. [*gene*]) denote deletion of more than a few base pairs within a gene. Forward slashes (e.g. *gene1*/*gene2*) denote mutations in the intergenic region between the two genes. Hyphens (e.g. *gene1*—*gene2*) denote deletions spanning multiple genes.
