# Supplementary material for: History of antibiotic adaptation influences microbial evolutionary dynamics during subsequent treatment
Source: PLoS Biol. 2017 Aug 8;15(8):e2001586. doi: 10.1371/journal.pbio.2001586 (PMC5549691; doi:10.1371/journal.pbio.2001586)
Supplement: S3 Table — (DOCX) [file pbio.2001586.s023.docx]

Supplementary Materials (S3 Table) for

History of Antibiotic Adaptation Influences Microbial Evolutionary Dynamics During Subsequent Treatment

Phillip Yen and Jason Papin*

*Corresponding author. E-mail: papin@virginia.edu

| Name | Lineage | Forward Primer Sequence (5'-3') | Reverse Primer Sequence (5'-3') | Position | Mutation |
| --- | --- | --- | --- | --- | --- |
| Paeru16SrDNA*^a^* | *P. aeruginosa* species | GGGGGATCTTCGGACCTCA | TCCTTAGAGTGCCCACCCG | - | - |
| PA_hmgA*^b^* | PIP^R^-1, -2, -3 | GCTGCCATCCACTCAAATTACG | GGGTTGGCTGGTTCATGG | 3435343 | ~ Δ 400kbp |
| P1_dacC | PIP^R^-1 | AACGCTTGTCACTGCTTGTCC | AGCGGAAGCCATAGGTCAGC | 1046490 | C→T |
| P2_orfJ | PIP^R^-2 | TCTGATAAAGATGGGCGAGACC | GACCTTCTCTGGCTGTTGACG | 2033788 | (G)7→6 |
| P3_mexR | PIP^R^-3 | TTCGCCAGTAAGCGGATACC | TTCGTTGCATAGCGTTGTCC | 486113 | T→G |
| P4_mucB | PIP^R^-4 | AGGCTCAGGTCGCTCAACG | ATCCTTCCCAACTGGCTTCC | 4824640 | G→A |
| T1_25490 | TOB^R^-1 | TGCCGATCATTCTGAGTTCG | CCACCGAGAGTTCCAGTTGC | 2229086 | T→C |
| T1_fusA1 | TOB^R^-1 | TCGATCGAGAAGCCGATCAT | AACATCTCACCCAGCGGAAC | 757307 | T→C |
| T2_fusA1 | TOB^R^-2 | CGCTGGTCGAAGTGAAGTCC | CAGGCGCTTCTTGATCTGC | 755747 | A→G |
| T3_rpsL | TOB^R^-3 | CGGGGCTTTGTCTTGACG | TGGCATCGAGAGCTTTTTCG | 754922 | A→G |
| T4_nuoL | TOB^R^-4 | TGAATTGCAGGGTCCATTCC | ACCTTCCGCCTGATCTTCG | 2587299 | Δ1 bp |
| F1_aotJ | CIP^R^-1 | TGGCCAGGAGCATGGAAAGC | GAGTTCGACGGCCTGATCCC | 4678735 | Δ1 bp |
| F2_aroB | CIP^R^-2 | ACGGTTCGTCGCAAATGAAACC | CTTGTTGCAGAAGCCCAACCC | 5946304 | +G |
| F3_sucD | CIP^R^-3 | CGGTCTGCGGATCTTCCTGG | CATCGTGCGTTGCGACATGA | 3912045 | T→G |
| F4_aroB | CIP^R^-4 | GCGTCCAAGATCTCACGGGG | GGCATGACCGCAAGACTACCC | 5945811 | G→T |

*^a^*Amplifies a portion of the 16S rDNA specific to *P. aeruginosa* species. Presented as the PA-SS primers in Spilker *et al* [1].

*^b^*Amplifies a portion of *hmgA* in *P. aeruginosa* PA14. Failure of amplification is used as a proxy for confirming large chromosomal deletion, since *hmgA* is consistently encompassed in all large deletions.

All primers were optimized to amplify DNA with an annealing temperature of 57°C with One*Taq* polymerase (New England Biolabs, M0483).

**References**

1. Spilker T, Coenye T, Vandamme P, LiPuma JJ. PCR-Based Assay for Differentiation of Pseudomonas aeruginosa from Other Pseudomonas Species Recovered from Cystic Fibrosis Patients. J Clin Microbiol. 2004;42: 2074–2079. doi:10.1128/JCM.42.5.2074-2079.2004
