# Supplementary material for: History of antibiotic adaptation influences microbial evolutionary dynamics during subsequent treatment
Source: PLoS Biol. 2017 Aug 8;15(8):e2001586. doi: 10.1371/journal.pbio.2001586 (PMC5549691; doi:10.1371/journal.pbio.2001586)
Supplement: S4 Table — Values denote the number of different lineages that had mutations in the specified gene for the given treatment. Values are not double counted if passed on from Day 20 to Day 40, e.g., a mutation that occurred in Day 20 PIPR that carried over to Day 40 PIPR, PIPRTOBR, and PIPRCIPR is counted as 1 lineage. (DOCX) [file pbio.2001586.s024.docx]

Supplementary Materials (S4 Table) for

History of Antibiotic Adaptation Influences Microbial Evolutionary Dynamics During Subsequent Treatment

Phillip Yen and Jason Papin*

*Corresponding author. E-mail: papin@virginia.edu

| Category | Locus tag | Gene | Description | # of lineages that have mutations in gene | | | |
| --- | --- | --- | --- | --- | --- | --- | --- |
|  |  |  |  | No drug | PIP | TOB | CIP |
| Multidrug efflux pumps | PA14_60850 | *mexC* | multidrug efflux RND membrane fusion protein |  | 3 | 2 |  |
|  | PA14_05520 | *mexR* | multidrug resistance operon repressor MexR |  | 4 | 1 |  |
|  | PA14_32420 | *mexS* | putative Zn-dependent oxidoreductase |  |  |  | 5 |
|  | PA14_16280 | *nalC* | putative transcriptional regulator |  | 4 |  |  |
|  | PA14_18080 | *nalD* | putative transcriptional regulator, TetR family |  | 4 |  | 1 |
|  | PA14_60860 | *nfxB* | transcriptional regulatory protein NfxB |  |  | 2 | 8 |
|  | PA14_41270 | *parS* | putative two-component sensor |  |  | 2 | 1 |
| Ribosome | PA14_08820 | *fusA1* | elongation factor G |  |  | 11 |  |
|  | PA14_08740 | *rplJ* | 50S ribosomal protein L10 |  |  | 1 |  |
|  | PA14_08750 | *rplL* | 50S ribosomal protein L7 / L12 |  |  | 1 |  |
|  | PA14_08790 | *rpsL* | 30S ribosomal protein S12 |  |  | 1 |  |
|  | PA14_09000 | *rplF* | 50S ribosomal protein L6 |  |  | 2 |  |
| NADH dehydro-genase | PA14_30010 | *nuoB* | NADH dehydrogenase I chain B |  |  | 1 |  |
|  | PA14_29940 | *nuoG* | NADH dehydrogenase I chain G |  |  | 1 |  |
|  | PA14_29880 | *nuoL* | NADH dehydrogenase I chain L |  |  | 1 |  |
|  | PA14_29860 | *nuoM* | NADH dehydrogenase I chain M |  |  | 2 | 1 |
| DNA and RNA | PA14_23260 | *gyrA* | DNA gyrase subunit A |  |  |  | 1 |
|  | PA14_00050 | *gyrB* | DNA gyrase subunit B |  |  |  | 2 |
|  | PA14_08780 | *rpoC* | DNA-directed RNA polymerase beta* chain |  |  |  | 1 |
|  | PA14_57940 | *rpoN* | RNA polymerase sigma-54 factor |  |  |  | 1 |
| Flagella | PA14_23380 | *orfH* | UDP-N-acetyl-D-mannosaminuronate dehydrogenase | 3 |  |  |  |
|  | PA14_23460 | *orfN* | putative group 4 glycosyl transferase |  |  | 1 | 3 |
|  | PA14_16430 | *wspA* | putative methyl-accepting chemotaxis transducer | 3 |  |  |  |
|  | PA14_25490 |  | putative tolQ-type transport protein |  |  | 2 |  |
|  | PA14_50440 | *flgF* | flagellar basal-body rod protein FlgF |  |  |  | 1 |
|  | PA14_50430 | *flgG* | flagellar basal-body rod protein FlgG |  |  |  | 1 |
|  | PA14_50360 | *flgK* | flagellar hook-associated protein 1 FlgK |  |  |  | 1 |
|  | PA14_45630 | *fliA* | motility sigma factor FliA |  |  |  | 1 |
|  | PA14_45770 | *fliP* | flagellar biosynthetic protein FliP |  |  |  | 1 |
| Cell wall | PA14_12100 | *dacC* | D-ala-D-ala-carboxypeptidase |  | 5 |  |  |
|  | PA14_11845 | *mpl* | UDP-N-acetylmuramate:L-alanyl-gamma-D-glutamyl- meso-diaminopimelate ligase |  | 4 |  |  |
|  | PA14_10800 | *ampR* | transcriptional regulator AmpR |  | 2 |  |  |
| Other | PA14_38510 | *hmgA* | Homogentisate 1,2-dioxygenase |  | 3 |  | 1 |
|  | PA14_09960 |  | putative transcriptional regulator |  |  |  | 3 |
|  | PA14_14470 | *pepA* | leucine aminopeptidase |  | 3 |  |  |
|  | PA14_04410 | *ptsP* | phosphoenolpyruvate-protein phosphotransferase |  |  | 2 | 1 |
|  | PA14_70470 | *spoT* | guanosine-3',5'-bis(diphosphate) 3'-pyrophosphohydrolase |  | 2 |  | 1 |
|  | PA14_66290 | *aceA* | pyruvate dehydrogenase, E1 component |  |  |  | 2 |
